# New York State Climate Impacts Assessment Chapter 03: Agriculture

**DOI:** 10.1111/nyas.15192

**Published:** 2024-12-09

**Authors:** Deborah Aller, Allison M. Chatrchyan, Alejandro Calixto, Jaime Cummings, Ariel Ortiz‐Bobea, Gregory Peck, Junior Schouten, Benjamin Weikert, Elizabeth Wolters, Amanda Stevens

**Affiliations:** ^1^ School of Integrative Plant Science—Soil and Crop Sciences Section Cornell University Ithaca New York USA; ^2^ New York State Integrated Pest Management Cornell University Geneva New York USA; ^3^ Syngenta Trumansburg New York USA; ^4^ Charles H. Dyson School of Applied Economics and Management Cornell University Ithaca New York USA; ^5^ School of Integrative Plant Science—Horticulture Section Cornell University Ithaca New York USA; ^6^ Big Apple Edibles New York New York USA; ^7^ Animal Science The State University of New York Cobleskill Cobleskill New York USA; ^8^ New York Farm Bureau Albany, New York, USA [now with New York State Department of Agriculture and Markets]; ^9^ New York State Energy Research and Development Authority Albany New York USA

**Keywords:** adaptation, climate change, crops, farmworkers, harvest, impacts, resilience, vulnerability

## Abstract

Agriculture is a vital industry in New York State, which ranks among the top‐producing states for dairy, fruits, and several other commodities. As agriculture depends on the weather and specific climatic conditions, this sector faces extraordinary challenges as New York's climate changes. This chapter explores the many impacts of a changing climate on agriculture, the ways these impacts interact with other challenges that New York farmers and farmworkers face, and opportunities for the agriculture industry to adapt and build resilience.

## 1 TECHNICAL WORKGROUP KEY FINDINGS

Agriculture is a vital industry in New York State, which ranks among the top‐producing states for dairy, fruits, and several other commodities. As agriculture depends on the weather and specific climatic conditions, this sector faces extraordinary challenges as New York's climate changes. This chapter explores the many impacts of a changing climate on agriculture, the ways these impacts interact with other challenges that New York farmers and farmworkers face, and opportunities for the agriculture industry to adapt and build resilience.

**Key Finding 1: The most severe impacts of climate change to the agriculture sector are associated with extreme precipitation, short‐term drought, heat stress, warmer winters, late spring freezes, increased pest pressures, and increased production costs**. Extreme precipitation damages crops, fields, and farm infrastructure; short‐term drought reduces crop yields and causes water shortages; heat stress affects livestock, crops, farmers, and farmworkers; late spring freezes after bloom cause losses in perennial fruit crops; and increased weed, disease, and insect pressures cause crop damage. Projected increases in temperature and precipitation extremes will cause these impacts to become more severe over time.

**Key Finding 2: Climate change is a threat multiplier for agriculture in New York State**. Farmers already face many stressors such as tight profit margins and labor shortages. Climate change exacerbates these stressors by producing more weather extremes, causing damage that requires unanticipated expenditures, and shortening operational windows. These stressors are further compounded in economically stressed, often rural communities and among historically underserved and vulnerable populations. Opportunities exist to address the negative effects, by both adapting to the direct climate impacts and managing the existing non‐climate stressors.

**Key Finding 3: Farmers and other agricultural stakeholders show awareness and acknowledgment of climate change impacts on agriculture**. Farmers and other agricultural stakeholders (e.g., extension agents, technical service providers, consultants) in New York are reporting increases in extreme weather events, variability, and uncertainty, which have disrupted common operations. Providing more information on anticipated changes, impacts, and solutions will help farmers plan, adapt, and remain profitable.

**Key Finding 4: Farmers are implementing and investing in practices that make their farm businesses more resilient to climate extremes**. Adaptation strategies depend on farm location and size, observed climate impacts, commodities produced, and costs. Many of these strategies, such as improving soil health, are beneficial for farms to adopt regardless of climate change and can also provide the cobenefits of reducing greenhouse gas emissions. While these adaptations are unlikely to fully alleviate the future climate impacts projected for New York, they are key to making the state's farms more resilient.

**Key Finding 5: Enhanced technical support, financial assistance, and research are crucial to increase the adaptive capacity of farms across New York State**. Farms will face greater risk of physical, social, and economic losses due to climate change without more support to implement adaptation measures. Active engagement between policymakers, farmers, and other agriculture stakeholders can help shape climate and agricultural policies and programs that are realistic for farm businesses.

BOX 1 **Developments since the 2011 ClimAID assessment**

The 2011 ClimAID assessment documented the challenges that climate change posed for the agriculture sector, the adaptation strategies used at that time, and new opportunities for the sector. The findings presented in the 2011 assessment remain accurate as of this report. Confidence in the extent of these climate impacts has increased, as has the associated need for adaptation strategies to address the impacts.

The 2011 assessment emphasized temperature, precipitation, and sea level rise as the main climate hazards, with factors such as the frequency and intensity of extreme events given a low certainty level. However, this level of certainty has now increased. For pests, the 2011 assessment highlighted Stewart's wilt and late blight as two pathogens that would increase in severity in New York State with the anticipated temperature and precipitation changes. This report discusses new invasive pests, such as the spotted lanternfly and soybean cyst nematode. It further supports the need for safer and more sustainable pesticide alternatives as climate change is changing pests’ distribution, seasonality, and susceptibility to pesticides.

The aquaculture industry has grown in parts of New York over the past decade and is a new focus here. Issues of climate equity and justice have gained attention and are presented in greater detail here than in the 2011 assessment. Urban agriculture continues to grow in New York State, and a case study highlights some of the challenges and adaptation strategies specific to agriculture production in urban areas.

Additionally, this chapter highlights how interconnected agriculture is with all other sectors (e.g., transportation, human health, buildings). It particularly emphasizes the need for a stronger local food system by exploring what was learned about the fragility of the food supply chain during the COVID‐19 pandemic. The cascading and compounding impacts of climate change are discussed in greater detail here than in the 2011 assessment. New policies and programs, at both the state and federal levels, are presented to reinforce the fact that climate change is occurring, and farmers and all agriculture stakeholders need greater support.

Overall, many of the issues highlighted in the 2011 ClimAID assessment remain as critical issues for agriculture across the state today. These are discussed throughout the remainder of this chapter. New challenges and opportunities to build resilience and adapt to climate change are also presented.

## 2 INTRODUCTION AND BACKGROUND

Climate change is becoming more acute and is increasingly affecting agriculture in New York and across the Northeast.^1^, ^2^, ^3^, ^4^ New York includes a complex diversity of landscapes and ecosystems and produces a wide range of agricultural goods, including dairy and other livestock, field crops, vegetables, fruit, maple products, specialty crops, and aquaculture products. This diversity makes a one‐size‐fits‐all approach to addressing the impacts of climate change nearly impossible. Agricultural producers in New York already face myriad challenges that make it difficult to farm profitably and sustainably, and these challenges are compounded by the changing climate and increases in extreme weather events.

It is also important to acknowledge that agriculture, like all sectors of the economy, contributes to climate change. The agriculture sector produces 6% of New York State's greenhouse gas emissions.^5^ Farmers can play a critical role in reducing greenhouse gas emissions (mitigation) while simultaneously implementing adaptation practices on their farms to build resilience to climate impacts. The concept commonly referred to as Climate‐Smart Agriculture (CSA) focuses on three pillars—mitigation, adaptation, and sustainable production—all of which are critical to responding to climate change in the agriculture sector.^6^, ^7^ Section 5.1 provides more detail on CSA. Examples throughout this chapter highlight synergies between these goals, whereby adaptation practices can also contribute to sustainable production and/or mitigation of greenhouse gases in the agriculture sector.

1 Towards the end of June, I found myself looking up at the sky and quietly praying for rain. Our fields were dry and our crops were struggling. The long dusty days were almost excruciating as we scoured the sky for hints and hopes of rain. And then, on the very last day of June, the skies opened, rain poured onto our parched fields, and our land came back to life. And now, we sit at the middle/end of July and I find myself looking up at the sky and quietly praying for a stretch of drier weather; we have seen nearly 14 inches of rain in the last three weeks. Where we flirted with drought this spring, now I half expect to see Noah ride by in his ark one evening.

—Sarah Ficken, dairy farmer, Madison County, New York (July 2021)

The primary climate‐related challenges New York farmers face include too much or too little precipitation, extreme weather events, shifts in growing season length, increasing variability and uncertainty (including warmer spring temperatures followed by hard freezes), heat stress, and increasing pressure from pests (insects, mites, plant diseases, weeds, and wildlife), and changes in phenology.^8^, ^9^ Large portions of the Northeast, including New York State, have experienced substantial crop losses due to both overly wet spring conditions and overly dry summer soil conditions,^8^ but every year varies.

These climate impacts exacerbate economic and labor challenges. Conversely, New York State's farmers could benefit from projected increases in overall water availability and longer growing seasons, if they can adapt and increase their resilience.

For agriculture in New York State and across the Northeast to remain sustainable, producers will need to increasingly adapt to a changing climate, building resilience and reducing risks to their operations.^3^, ^10^ Making these changes will require substantial financial investments. Farmers in New York and in the Northeast report that although they understand there are climate impacts and weather‐related threats to their farms, many lack the skills, technical knowledge, and financial capacity to address these impacts.^9^, ^11^ They will need greater access to technical knowledge and support to adapt. Adaptation to the challenges discussed in this chapter will help to ensure the future resilience and success of New York State agriculture.

The remainder of this background section provides an overview of the agriculture sector in New York State, along with an introduction to the climate hazards affecting agriculture and their social and economic impacts. The other sections of this chapter provide a more detailed look at impacts and adaptation in New York's agriculture sector:

- **Section** **3** provides an overview of the current and projected impacts of climate change on key agricultural commodities in New York State, specifically field crops, perennial fruit crops, vegetable and horticulture crops, dairy and livestock, maple syrup, and aquaculture. This section also describes cross‐commodity and cross‐sector impacts. In addition to impacts, Section 3 discusses specific adaptation strategies producers are already implementing, as well as practices and technologies that farmers could expand to cope with the uncertainties of a changing climate.
- **Section** **4** discusses climate justice considerations and disproportionate impacts on vulnerable communities, including Indigenous Peoples, undocumented farmworkers, urban farmers, and other underserved populations. It also discusses the many nonclimate challenges that intersect with climate change to make agriculture particularly vulnerable to the changes projected for New York's climate.
- **Section** **5** broadens the discussion of adaptation to include overarching strategies, financial and other resources available to support adaptation, and an assessment of how well these efforts have worked to date.
- **Section** **6** concludes the chapter by looking at emerging research needs and opportunities that lie ahead for New York's agriculture sector. The section also provides a conclusion, summarizing the major findings and recommendations presented in the chapter.
- The **Traceable Accounts** appendix examines each key finding in depth. It provides citations that support each assertion, and it presents the authors’ assessment of confidence in each finding.
- **Case studies** highlight the climate impacts farmers and communities are experiencing firsthand as well as the adaptation and resilience strategies they are employing to minimize the effects on their livelihoods. These case studies are not included in the chapter proper but are available through links provided in the chapter.

### 2.1 Sector scope and context

The U.S. Department of Agriculture (USDA) Economic Research Service defines a farm as any place that produces and sells, or normally would produce and sell, at least $1000 of agricultural products each year.^12^ New York State is home to 33,400 farms on 6.9 million acres of land, with the average farm occupying 207 acres. The state's agriculture sector contributes over $5.3 billion to New York's economy.^13^, ^14^

New York State's agriculture is diverse and includes dairy, vegetables, grapes, fruits, aquaculture, livestock, row crops, and nursery and ornamental crops. Table 3-1 summarizes the most recent statewide agriculture statistics.^14^ Section 3.2 presents important regional differences that influence agriculture across the state. More detailed information on some key New York products is provided in commodity‐specific sections later in the chapter.

**TABLE 3-1:** New York State agriculture product rankings and values for 2017.^a^

Product	Number of farms	Sales ($1000)	NYS rank by sales	% total sales	U.S. state rank
Milk from cows	3984	2,528,282	1	47.1	3
Grains, oilseeds, dry beans, and dry peas	6213	571,706	2	10.6	28
Cattle and calves	10,197	426,026	3	7.9	31
Fruits, tree nuts, and berries	3083	399,803	4	7.4	7
Nursery, greenhouse, floriculture, and sod	2118	385,792	5	7.2	10
Vegetables, melons, potatoes, and sweet potatoes	3588	378,658	6	7.1	12
Other crops and hay	13,670	362,905	7	6.8	12
Poultry and eggs	4146	194,747	8	3.6	29
Horses, ponies, mules, burros, and donkeys	1591	33,727	9	0.6	9
Hogs and pigs	1835	24,920	10	0.5	30
Other animals and other animal products	1609	22,761	11	0.4	20
Sheep, goats, wool, mohair, and milk	2235	17,575	12	0.3	15
Aquaculture	105	13,187	13	0.2	24
Christmas trees and short rotation woody crops	763	9122	14	0.2	8
Total	33,438^b^	5,369,212		100.0	27^c^

*Note*: Data from USDA, National Agricultural Statistics Service (2019).^14^

^a^ The last published USDA Census of Agriculture is from 2017; the 2022 Census of Agriculture data will not be published by USDA's National Agricultural Statistics Service until 2024.

^b^ Total number of farms in New York State as of the 2017 Census, not the sum of the product tallies in the “Number of farms” column.

^c^ New York's state ranking for total sales of agricultural products.

#### 2.1.1 Demographics

According to the 2017 USDA Census of Agriculture, the overwhelming majority of farmers captured in the census were white. The demographic breakdown of farmers in the state is as follows: 57,155 farmers identify as white; 606 identify as Hispanic, Latino, or Spanish origin; and 139 identify as Black or African American. The majority of farmers are men, with the Census reporting 35,985 males, compared with 21,880 females.^14^ Although not reported in the Census, there are farms in New York operated by Indigenous Peoples, including by farmers who are citizens of the Seneca, Onondaga, Cayuga, Oneida, Tuscarora, and Shinnecock Nations. Half of all farmworkers in New York State are undocumented immigrants.^15^ While urban agriculture has grown in the state, farms continue to be primarily located in more rural areas.

### 2.2 Key climate hazards

Agricultural production is highly dependent on stable weather conditions and highly sensitive to changes in the climate.^16^ Climate change already poses a real threat to agriculture and is predicted to cause a reduction in global agricultural productivity of up to 17% by 2050.^17^ Critical climate factors affecting agriculture include changes in temperature (and increases in the number of extreme heat events); changes in the amount and duration of precipitation (and increases in extreme events); sea‐level rise (and the related issue of saltwater intrusion into agricultural fields and groundwater sources used for irrigation); changes in pest and disease pressure; and increasing variability in many of the specific hazards listed here.^16^ Chapter 2, New York State's Changing Climate, provides an in‐depth discussion of many of these hazards. In addition, changes in temperature and precipitation lead to a host of other changes that affect agricultural productivity and agricultural management decisions, including changes in soil moisture, timing of frost (and freeze risk), growing season length, health impacts to farmers and farmworkers, and numerous other climate‐related variables.^2^, ^16^

These climate hazards contribute to a variety of impacts to agricultural operations, including:

- **Crop and livestock impacts**. Climate‐related stressors impair growth and reproduction in both plants and animals and create the need for interventions to adapt. Such stressors include animal heat stress, crop‐growing region migration, and changes in leaf wetness duration. Importantly, climate change can also provide new opportunities in regions that formerly had climate conditions that were incompatible with the production of certain crops.^16^
- **Biological impacts**. Growers will need to consider the establishment, spread, and impacts of pest species within agricultural systems. Biological impacts include changes in weed range and infestation intensity, crop damage by insects and other invertebrates, and crop diseases.^18^ These changes, in turn, will influence the use of pesticides to control these pests.^16^
- **Phenological impacts**. Phenology focuses on the timing of phenomena related to the seasonal cycles of plant and animal life and can include a broad range of events, such as plant leaf‐out and bloom, animal hibernation, and insect emergence and life cycles.^19^ The influence of climate change on phenology is revealed by changes in winter chill units, disease vectors in livestock, pollinator availability, and the timing of budbreak in perennial fruit crops.^19^
- **Socioeconomic impacts**. There is growing recognition that the impacts of climate change on agricultural production extend beyond the biophysical issues discussed. Climate change affects the health and well‐being of farmers and farmworkers, as well as operational costs, food storage and transportation, commodity prices, food prices paid by consumers, and food security.^20^ Because climate change may be just one of many factors affecting socioeconomic conditions, it can be difficult to quantify the exact causation or the magnitude of effects caused by climate change alone; there is often a lack of available or accurate data about these socioeconomic impacts.^16^ Tracking crop insurance payments, total factor productivity (TFP), and heat‐related mortality of agricultural workers can provide useful data about climate change impacts to the agriculture sector.^16^

These climate‐related changes affect the short‐ and long‐term decisions that farmers need to make. As noted in the USDA report Climate Indicators for Agriculture: “An example of a near‐term decision that requires reliable climate information might be when to plant or harvest; a mid‐term decision might be what variety of seed to plant for the following growing season; and a long‐term decision might involve whether to make capital investments, such as irrigation infrastructure, installing subsurface drainage tile, or planting trees in an agroforestry system.”^16^

Section 3 of this chapter describes many of these agriculture‐specific climate impacts in greater detail.

### 2.3 Nonclimate factors

Farming is, by nature, a risky business due to weather fluctuations and other factors. As a result, farmers in New York State face many challenges that are not directly related to climate change. Indeed, when asked about the challenges they face, farmers make it clear that climate change is rarely their top issue of concern; rather, it is a factor that exacerbates other challenges.^21^ Section 4.4 describes in detail how climate change can add to the many other nonclimate stressors that farmers currently face, including labor cost and supply challenges, land acquisition, other competing land uses, broadband access, supply chain disruptions, structural changes in markets, food retailer consolidation, and state and federal regulations. All these factors, from labor to the COVID‐19 pandemic to regulations, are interconnected issues that affect agricultural production in New York State, and climate change is a compounding factor that can exacerbate social, economic, and political issues.^22^

### 2.4 Equity and climate justice

There is growing recognition that climate change disproportionately affects vulnerable and overburdened populations, including in the agriculture sector. These populations are often situated in more vulnerable areas with greater exposure to climate impacts, and they have less capacity to respond to climate change impacts, which in turn further amplifies inequity and injustice.^23^ As discussed in the Assessment Introduction, climate equity is the principle that all residents have a fair and just opportunity to live, learn, work, and play in a safe, healthy, resilient, and sustainable environment, even as the climate changes. Climate justice is the promotion of individual and collective capacity to prepare for, respond to, and recover from climate events, as well as fair treatment, meaningful involvement, and absence of discrimination in the creation of policies, programs, and projects that both address the disparate impacts of climate change and the transition to a net zero emissions economy. Climate justice requires that inequities be addressed head‐on through long‐term adaptation^24^ and that adaptation practices do not make inequality worse.^25^ The focus on climate justice means that there should be an equitable division, sharing, and distribution of the burdens of climate change impacts and solutions to address them, and there should also be an equitable division, sharing, and distribution of any potential benefits.^26^ Climate justice acknowledges that those who are least responsible for climate change are affected most acutely by its impacts.^27^

In the agriculture sector, vulnerable communities include:

- Farmers (especially new and older farmers) and farmworkers in general, whose physical and mental health can be affected by climate change impacts such as heat stress and the increase in extreme precipitation events on their farms.
- Low‐income farmers or those from historically overburdened groups (e.g., farmers of color), who may have fewer resources to purchase high‐quality land for farming or respond to extreme events that threaten their farm businesses.
- Low‐income populations and communities more generally that are affected by disruptions in the supply chain and rising food costs due to climate impacts on crop yields.

In 2020, the Climate Justice Working Group was created in New York State to help identify and advise on the needs of disadvantaged frontline communities in key industries across the state, including agriculture. This chapter highlights key climate equity and justice considerations in the agriculture sector and how efforts to build resilience can acknowledge and help to address longstanding inequities.^28^

### 2.5 Indigenous communities

Indigenous Peoples rely on the land for food, medicine, and traditions, which puts them on the front line in experiencing climate change impacts and finding ways to adapt. Tribal Nations experience greater exposure to climate‐related risks because the historical colonization of large swaths of their traditional territories, taking of productive agricultural lands, and forced migration left them to farm lower‐quality lands.^29^ Nine federally recognized or state‐recognized Tribal Nations are located in New York State, and several other communities of Indigenous Peoples maintain ties to the state and live in surrounding states.^30^

As of 2017, 78 farms in New York State were designated as “Institutional, Research, Experimental or American Indian Reservation Farms,” but there are no specific data available on the number of farms on present‐day Tribal lands. Only 125 farmers in New York identified as American Indian/Alaska Native in the most recent Census of Agriculture,^14^ but the actual number is likely higher because the official USDA definition of a farm does not fully reflect certain cultural cultivation practices and traditions of Indigenous Peoples in New York. Therefore, it leaves out many Native American New Yorkers who are practicing farmers.

Indigenous Peoples across the United States, including in New York, are experiencing change through loss of native species of cultural importance, and loss of productive lands due to erosion and flooding.^31^ Climate change threatens some key species that are important for food and other purposes among Indigenous Peoples. One example is the hard clam (northern quahog), which is essential to the Shinnecock Nation for both sustenance and wampum‐making. In the face of these changes, Indigenous Peoples are resilient and innovative when it comes to adapting to the resource base that remains. Refer to the Shinnecock Nation Marine and Land Farming Adaptations case study for more information on the importance of shellfish and adaptation strategies.

## 3 OBSERVED AND PROJECTED IMPACTS AND ADAPTATIONS

This section provides a detailed discussion of observed and projected climate impacts for New York State's agriculture sector. It begins with a summary of the climate hazards or physical changes that affect agricultural activities. Next, the section reviews commodity‐specific impacts and highlights the specific adaptations available to address these impacts, as there is no “one‐size‐fits‐all” approach to agricultural adaptation. The section also discusses the impacts and adaptation measures that span multiple commodities. Table 3-2 provides a summary of potential adaptation strategies to help address the specific climate impacts that affect each key commodity sector in New York. While the table may not cover all possible adaptation strategies, the goal is to introduce some of the adaptation strategies, practices, and opportunities available to farmers to increase the resilience and sustainability of their farm operations.

**TABLE 3-2:** Summary of climate change impacts and examples of potential adaptation strategies by commodity.

**Field crops**	
Heat stress	• Shift planting dates earlier when possible. • Use heat‐tolerant varieties and crops. • Plant shorter‐season crop varieties.
Drought stress	• Improve soil health—reduced or no tillage, plant cover crops, use soil amendments. • Use drought‐tolerant crops and varieties. • Reduce row spacing to shade soil.
Warmer winters	• Plant winter wheat and barley varieties that are less susceptible to freeze/thaw damage.
Extreme precipitation	• Improve drainage (e.g., install tile drainage and dig drainage ditches). • Create buffer zones around waterways to reduce erosion and runoff. • Invest in practices to build soil health—reduced or no tillage, plant cover crops, use soil amendments, use double cropping.
Pests (invertebrates, weeds, plant diseases)	• Use integrated pest management—cultural, biological, mechanical, genetic, and chemical control practices with scouting and thresholds. • Consult online pest forecast systems. • Plant varieties with improved pest and disease resistance. • Plant varieties with more biotechnology traits for herbicide resistance. • Use double cropping and intercropping.
**Perennial fruit crops**	
Warmer winters	• Plant new varieties and rootstocks. • Avoid planting on marginal sites.
Heat stress and drought	• Expand irrigation, especially for new plantings. • Use evaporative cooling with over‐the‐row sprinklers. • Use protective covers. • Conduct labor‐intensive activities (such as fruit thinning and harvest) during the night by using lights and mechanical platforms.
Extreme rainfall and hail	• Use modified greenhouse approaches and controlled‐environment agriculture, such as high‐tunnels for more valuable crops or whole‐orchard fabric covering systems. • Install tile drainage for new plantings.
Pest invasions	• Prioritize early detection and rapid response. • Use integrated pest management strategies and increase use of biological control tools.
Freeze risk	• Use techniques that minimize frost damage, including wind machines, over‐the‐row sprinklers, whole‐orchard covering systems, and generating heat by burning oil in smudge pots.
Management challenges	• Consult online pest forecast systems. • Engage in adaptation planning. • Make financial investments (e.g., in infrastructure and equipment) against multiple extremes, such as too much or too little precipitation, or winters that are too warm (causing early budbreak) or too cold (causing winter injury). • Use sites that are conducive to predicted climate extremes.
**Vegetables and horticulture crops**	
Warmer winters	• Experiment with new varieties; breed new cultivars. • Consider winter production in protected environments (high tunnels).
Warmer summers, heat stress, and droughts	• Expand irrigation and invest in micro or variable‐rate systems. • Expand use of high tunnels and controlled‐environment agriculture. • Shift planting dates. • Invest in soil health practices. • Use grafted plants with more vigorous root stock. • Use heat‐tolerant varieties.
Extreme precipitation and water stress	• Shift production zones away from flood‐ and frost‐prone areas. • Expand storage and drainage (e.g., install tile drainage and dig drainage ditches). • Invest in soil health practices—reduced or no tillage, plant cover crops, use soil amendments, practice double cropping. • Use raised beds. • Protect critical infrastructure.
Pests (invertebrates, weeds, plant diseases)	• Use integrated pest management—cultural, biological, mechanical, genetic, and chemical control practices with scouting and thresholds. • Plant cover crops and trap crops. • Consult online pest forecasts and use decision‐support tools. • Use pesticides only when needed, based on thresholds. • Remove plants that are hosts to invasive pests.
Sea level rise	• Move planting areas away from flood zones or coastal floodplains. • Plant salt‐tolerant crops (inherent or genetically improved). • Establish salt‐tolerant plant buffers between fields and water. • Grow value‐added, alternative crops.
**Dairy, livestock, and livestock products**	
Heat stress	• Upgrade or expand cost‐effective housing ventilation and cooling systems. • Provide more shade in pastures. • Adjust feeding management. • Increase water intake. • Breed for specific heat tolerance. • Consider using silvopasture.
Drought conditions	• Closely manage pasture and reduce stocking rate. • Practice rotational grazing. • Consider using silvopasture.
Pests (invertebrates, weeds, plant diseases)	• Use integrated pest management—cultural, biological, mechanical, genetic, and chemical control practices with scouting and thresholds. • Improve disease monitoring and ability to quarantine. • Use only the amount of pesticides directed, at the time and under the conditions specified, following all safety guidelines.
Extreme precipitation and flooding, and impacts to water quality	• Closely manage pasture. • Alter design and/or location of animal housing, feed storage, and manure management structures to avoid flood damage. • Improve manure management, including through increases in manure storage capacity and use of flood‐resistant practices. • Use manure management tools to time spreading based on weather forecasts. • Adopt best management practices (e.g., erosion controls, riparian buffers) to protect water quality.
Animal diseases and parasites	• Improve disease monitoring and ability to quarantine. • Identify and improve breeds and hybrids that have greater disease resistance. • Adjust feed nutrient utilization and intake. • Expand housing sanitation, ventilation, and cooling systems. • Increase water intake. • Increase surveillance of parasites and pests that affect livestock productivity and health.
**Agroforestry and maple products**	
Warmer winters	• Shifts in season—divide tasks and labor. • Implement tap hole sanitation techniques to prevent reduction in sap flow due to microbial plugging. • Use vacuum pump technology. • Use plastic tubing, check valve spouts, replace droplines as necessary. • Use a reverse osmosis system. • Diversify production of agroforestry products and crops.
Increased wildfire	• Develop forest wildfire management plans and perform controlled burns.
Pests (invertebrates, weeds, plant diseases)	• Increase crop diversity. • Provide habitat for beneficial insects.
Extreme temperatures and drought	• Provide shading and shelter opportunities for animals. • Use windbreaks to reduce windspeed and reduce evapotranspiration. • Create microclimates to buffer crops from extreme temperatures.
**Aquaculture**	
Warmer water temperatures	• Improve monitoring of species populations, disease, and ecosystem health. • Use selective breeding to identify genetic strains that are more resilient to temperature variation. • Relocate infrastructure, if feasible.
Ocean acidification	• Apply sediment amendments. • Diversify products and business models. • Use integrated aquaculture.
Pests	• Improve monitoring of species, populations, disease, and ecosystem health. • Identify disease‐resistant species.
**Crosscutting impacts and adaptation strategies**	
Uncertainty	• Use precision farming apps and weather and climate tools to make more informed decisions.
Management challenges	• Develop an adaptation plan for the farm. • Maximize fuel efficiency and decrease labor and time constraints. • Hire custom operators.
Risk	• Diversify agricultural production, products, and cropping systems. • Consider controlled‐environment agriculture, if applicable. • Renovate or build new energy‐efficient farm buildings to better withstand extreme weather. • Consider purchasing crop insurance to reduce economic risks.
Pests	• Use integrated pest management—cultural, biological, mechanical, genetic, and chemical control practices with scouting and thresholds. • Use alternatives to chemicals, such as electronic weed zappers, disease resistant varieties, row covers, mulches, and so on. • Grow alternative species that are not hosts for pests. • Breed for improved disease resistance.
Pollinators	• Plant native species and a diversity of plants. • Reduce/eliminate pesticide use if possible; use only the amount of pesticides directed, at the time and under the conditions specified, following all safety guidelines. • Install bat boxes. • Establish pollinator gardens and provide habitat through agroforestry practices.

*Note*: Table incorporates information from Tobin et al.^1^ and Climate Smart Farming Program, Cornell University (2022).^32^

### 3.1 Climate hazards

This section discusses climate hazards or physical changes that affect agricultural activities in New York State.

#### 3.1.1 Temperature change

Average air temperature statewide has increased by almost 2.6°F from 1901 to 2022, and the warmest 10‐year periods in recorded history have occurred since 2000.^33^ As detailed in this assessment's Chapter 2, New York State's Changing Climate, temperatures are projected to continue to increase across the state. Projections developed for this assessment show a likely increase in mean temperature of 2.5–4.4°F by the 2030s, 3.8–6.7°F by the 2050s, and 5.1–10.9°F by the 2080s, relative to a 1981–2010 baseline.^33^ Warming of this magnitude will lengthen the growing season but could also threaten the health and productivity of livestock and some types of crops, as described in Section 3.3.

Seasonal changes in temperature matter to agriculture, too. As detailed in New York State's Changing Climate, observed temperature increases have been largest in the winter, followed by spring and fall, all of which leads to a lengthening of the growing season. Warmer winters will increase survival and spring populations of some insects^34^ and other pests that currently overwinter in the state on a limited scale. Warmer winters could also affect the suitability of various perennial crops, as well as yields and quality.^35^ However, warmer temperatures and longer growing seasons could increase yields and expand market opportunities for some crops.^36^

#### 3.1.2 Nighttime air temperature

New York State has experienced an increase in the number of warm nights (where the minimum temperature is 70°F or higher), and a decrease in the number of very cold nights.^33^ Both of these changes have implications for agriculture. Cattle, for example, are at greater risk of heat stress when nighttime temperatures remain warm (above 73°F for one night, or above 70°F for three consecutive nights).^34^ Stressful conditions over several consecutive days and nights can be detrimental to production and, in some cases, harmful to animal health. As Section 3.3 discusses in depth, environmental stress has many negative physiological impacts and is detrimental to animals’ immune and other bodily systems.

#### 3.1.3 Heat waves and humidity

Heat waves and humidity are important factors for the health of both livestock and farmworkers. The number of very hot days is projected to increase in the coming decades.^33^ The frequency and duration of heat waves, defined as three or more consecutive days with maximum temperatures at or above 90°F, are also expected to increase. By the 2080s, all regions of the state are projected to have at least three heat waves each year.^33^

Humidity levels also influence heat stress in both humans and livestock. Increases in surface temperatures can raise the humidity, as warmer air can hold more water vapor, and warming seas and land surfaces send more water into the atmosphere through evaporation. As a result of global increases in both temperature and humidity, researchers project heat stress will intensify in the future.^37^

Heat and humidity could cause increased stress on livestock, reduced yields, and increases in heat‐related illness or death among agricultural workers. Heat‐related illness or death can occur in any worker (including agricultural workers) exposed to conditions of high temperatures and humidity where the human body cannot cool itself sufficiently through sweating.^38^ (This topic is discussed further in Section 4.1.)

#### 3.1.4 Freeze risk

Cold temperatures are endemic to New York State, and they are vital to some agricultural commodities such as fruit trees that require winter chilling and maple syrup production that depends on freeze‐thaw cycles. While the number of days below freezing and the number of days with extreme cold (here defined as a temperature at or below 0°F)^39^ are both expected to decrease statewide during the remainder of this century, all regions of the state will continue to experience freezing temperatures.^33^

1 Frost dates have changed both in the spring [earlier] and fall [later], the growing season has definitely gotten longer, but we are hesitant to make changes in our planting decisions and take advantage of this because they [frost dates] are just so variable and unpredictable.

—Michael Glos, organic vegetable and livestock farmer, Tioga County, New York (October 2022)

Of arguably greater concern to agriculture are changes in the timing of freeze events and the increasing variance in temperature. An increase in temperature variance of less than 5% leads to increased freeze risk, despite warming temperatures.^40^ As milder winter temperatures lead to earlier leaf emergence and bloom, crops will become more vulnerable to frost and freeze events that occur later in the spring at critical stages of fruit development.^40^, ^41^ Late‐spring frosts—those that occur after germination of herbaceous plants and budburst of woody plants—will increase in severity of damage due to climate change.^41^ Apples and other crops in the state have experienced increasing damage in recent years due to late freeze events,^40^ as discussed in Section 3.3.

#### 3.1.5 Growing season length

Another climate factor important to agriculture is the length of the growing season, which in simple terms refers to the number of days in which plant growth takes place. Changes in growing season length can have both positive and negative effects on yields and farm profitability, depending on the crop.

When calculated as the length of time between the last spring frost and the first fall frost, New York's growing season increased by 11.5 days over the period from 1895 to 2020.^42^ With temperatures projected to warm in all seasons during the 21st century,^33^ growing seasons can reasonably be expected to continue to lengthen.

#### 3.1.6 Precipitation changes

The water demand of crops varies over the course of the growing season, is crop‐dependent, and is influenced by farm management practices and current environmental conditions. Rising carbon dioxide concentrations, higher temperatures, changing precipitation patterns, and more variable humidity levels are four climate‐related hazards that will affect plant water use.^43^

Total annual precipitation has increased in New York State by 10−20% since 1901.^44^ However, the timing of precipitation matters, especially for agriculture. Unlike other regions of the United States that face perennial droughts, the Northeast often faces a “double‐edged sword”—experiencing an excess of rainfall, and then not enough water, often in the same season.

New York is expected to experience an increase in spring rainfall,^33^ making it difficult for farmers to get onto fields to plant their crops. Too much water can also lead to saturated soil conditions, increasing the risk that water will pond on the surface or run off from farm fields, which can have negative consequences for soil erosion and water quality (e.g., harmful algal blooms [HABs]). In contrast, precipitation projections are inconclusive for summer,^33^ when crops need water to grow, but there is some evidence of recent decreases.^45^ The combination of insufficient rainfall and higher summer temperatures increases the risk of short‐term droughts, which in turn can increase the quantity of water needed for irrigation. In 2016, for example, New York state's agricultural regions were affected by a drought. In response to a statewide survey, farmers estimated crop losses of 41% for forage crops, 42% for pasture, 33% for soybeans, 31% for field corn, and 17% for small grains when averaged across the state.^46^

Changes in the timing and intensity of individual rainfall events are the most important precipitation changes impacting the agriculture sector. Climate projections developed for this assessment predict that precipitation across the state will increase by approximately 2−12% by the 2050s, and 6−17% by the 2080s, relative to a 1981–2010 baseline.^33^ These scenarios seem to suggest the possibility of decreasing drought risk, but for several reasons, this is far from certain. First, projected changes in the distribution of precipitation toward larger rainfall events could lead to longer dry spells, which could result in short‐term drought in some parts of the state, especially during the growing season, when farmers need the rain. Second, higher mean temperatures during the summer could lead to large increases in evapotranspiration, which would mean that more precipitation or irrigation is required to maintain soil moisture levels. Third, reductions in snow cover associated with warming could increase flood risk in the winter and spring while also increasing drought risk in the summer as soils dry out earlier with higher temperatures.^33^

#### 3.1.7 Extreme precipitation

The number of extreme precipitation events (defined as 2 inches or more of precipitation within a 24‐h period) has increased in New York State over the past several decades.^39^ However, the frequency and intensity of these events differs between the coastal and inland regions of the state.^33^

According to the New York Farm Service Agency, “the most common cause of loss for crops in the past ten years has been from excessive moisture, especially for fruit and vegetables, followed by freeze events that affect fruits, and drought. Normally the highest number of payments are triggered in the fall for our Non‐Insured Crop Disaster Assistance program. Most crops affected have harvest completion in the fall and payments are made after, when we know if a farmer had enough yield losses to trigger a payment” (New York State Farm Service Agency [2022, July, Personal communication]).

These trends are expected to continue. Climate models project an increase in total annual precipitation, as well as increases in the frequency and intensity of extreme precipitation events through the 21st century.^33^ However, projecting future precipitation extremes remains challenging.

#### 3.1.8 Changes in soil moisture

Soil moisture refers to the water stored in the soil. Changes in precipitation, temperature, soil properties, soil evaporation, plant transpiration, solar radiation, vapor pressure, and wind all affect soil moisture.^47^ Because soil moisture is spatially heterogeneous, it is difficult to model on global and even regional scales.^48^ Researchers use soil moisture levels to assess drought conditions; numerous studies support the hypothesis that soil moisture will decline under future climate scenarios.^49^, ^50^ Increasing short‐term drought, especially during the summer months, can lead to a decrease in soil water availability for rainfed crops, increasing crop stress and disrupting overall function and growth. Sustained soil moisture deficits (drought) can lead to reduced crop yields and potentially major crop losses, depending on length and severity. Collecting soil moisture data as temperatures rise and precipitation patterns shift statewide is increasingly important.

1 Last year [2021], we literally would get ½ inch of rain in five minutes, and we have a Mesonet [weather] station so we know this…So we have gone to protected culture for 95% of our crops, mostly plastic covered high tunnels and exclusion netting for our blueberries, which helps diffuse wind and rain in addition to providing pest control. This cost us about $200,000, but now we get essentially no weather‐related losses.

—Dale‐Ila Riggs, vegetable and berry farmer, Rensselaer County, New York (April 2022)

#### 3.1.9 Sea level rise, saltwater intrusion, and ocean acidification

Sea level has risen by about 13 inches relative to the New York State coastline since 1880, much more than the global average rise of about 7–8 inches since 1900.^33^, ^39^ Sea level is projected to rise along the state's coastline by up to 1 foot by the 2030s, about 2–3 feet by the 2080s, and more than 4 feet by 2150, relative to a 1995–2014 baseline, as discussed in more detail in New York State's Changing Climate.^33^ Under a rapid ice‐melt scenario that involves more rapid loss of ice from the large ice sheets of Greenland and Antarctica, the high‐end estimate for sea level rise increases further.

As sea level rises, the risks of higher tides and storm surge, coastal flooding, and saltwater intrusion increase. Many farms on Long Island have already experienced these effects, with more than 800 acres of farmland flooded with saltwater during Superstorm Sandy in 2012.^51^ Saltwater intrusion is affecting farms and the groundwater wells used for irrigation further inland.^52^ The most common causes of saltwater intrusion include: (1) drought that reduces the amount of natural recharge to the groundwater system; (2) pumping that extracts too much water, or pumping from a well that is too close to the freshwater/saltwater interface; and (3) sea level rise that causes a rise in the water table. Unfortunately, once saltwater enters the freshwater system, it is difficult to reverse.^52^ The U.S. Geological Survey is working to map the freshwater/saltwater interface on Long Island to evaluate the overall sustainability of the aquifer system and allow for more accurate placement and depth of wells.^53^

In addition to causing direct harm to crops, saltwater flooding and intrusion accelerate the release of nutrients such as phosphorus from the soil into water bodies, which has important implications for surface water quality.^54^, ^55^ Saltwater intrusion also changes the redox cycling dynamics of coastal soil ecosystems by increasing the mineralization of soil organic carbon and contributing to carbon dioxide production. Thus, carbon dioxide emissions from coastal systems are expected to increase as seawater levels rise.^56^

Sea level rise and flooding can also damage roads, bridges, and other infrastructure, making it more difficult for farmers to get their products to markets.^39^

While ocean acidification due to climate change is a global problem, local factors such as upwelling, riverine discharge, and eutrophication exacerbate the acidification of the state's coastal ecosystems.^57^ Warmer waters and excess nutrients, attributed to many sources including residential, industrial, and agricultural sources, lead to increased microbial respiration by aquatic organisms. This eutrophication‐enhanced microbial respiration further increases carbon dioxide levels in coastal waters.^58^, ^59^, ^60^ Elevated carbon dioxide levels are known to negatively impact calcifying organisms by slowing shell and tissue growth, and more recent research suggests that increased carbon dioxide levels can also increase the growth of macroalgae.^57^, ^61^, ^62^ All these changes have potential implications for the aquaculture industry in New York State (Section 3.3.6).

A discussion of the broader impacts of sea level rise, saltwater intrusion, and ocean acidification, beyond the agriculture sector, is provided in the Ecosystems chapter.

### 3.2 Regional differences

Each region of New York State will experience the impacts of climate change differently due to variations in agricultural operations, regional microclimates, plant hardiness zones, and soil diversity.

Food, fiber, nursery, and seafood production occur statewide. In densely populated urban environments like New York City, where land for agriculture is limited, agricultural activity takes place largely in community gardens, rooftop farms, and indoor vertical farms.^63^ Large farms—some thousands of acres—that produce grains, vegetables, and fruits operate in more rural parts of the state. Animal production includes longstanding industries, such as dairy and poultry (including eggs), but also increasingly diversified operations that focus on value‐added products, such as yogurt and cheese. Aquaculture is a growing component of New York's agriculture landscape, with seaweed farming and fish and shellfish production occurring in the waters around Long Island and in indoor facilities in other parts of the state.^64^

Most of New York State is defined as having a humid continental climate.^65^ However, the maritime ecosystem in the southeastern part of the state and the many lakes and river valleys found throughout the state afford a wide range of microclimates for growing specialty crops, such as apples and grapes. This diversity of climatic features means that the state's weather varies considerably; thus, climate change impacts and adaptation needs also vary. Projections developed for this assessment show that temperatures will warm most rapidly in the northern part of the state, where many dairy farms operate.^33^ The projections also show that heavy precipitation increases will be greatest in the southeastern part of the state, which could increase flooding for farms.^33^

New York's plant hardiness zones, which are based on the average annual extreme minimum winter temperature, currently range from Zone 7 on Long Island and New York City (0°F to 10°F) to Zone 3 in the Adirondacks and St. Lawrence Valley regions (−40°F to −30°F). Plant hardiness zones aid in determining the type of agricultural production that can occur in a region because they are used to evaluate the cold hardiness of plants. Importantly, these zones are shifting in response to a changing climate^66^ and are expected to continue to change, with minimum winter temperatures increasing across the state.^67^ This shift in plant hardiness zones will negatively affect the production of some crops while allowing for the introduction of others.^8^

Figure 3-1 compares the plant hardiness zones that existed from 1980 to 2009 with those projected for the period 2070−2099 under the very high emissions scenario (SSP5‐8.5) used elsewhere in this assessment and described in New York State's Changing Climate.

**FIGURE 3-1:**
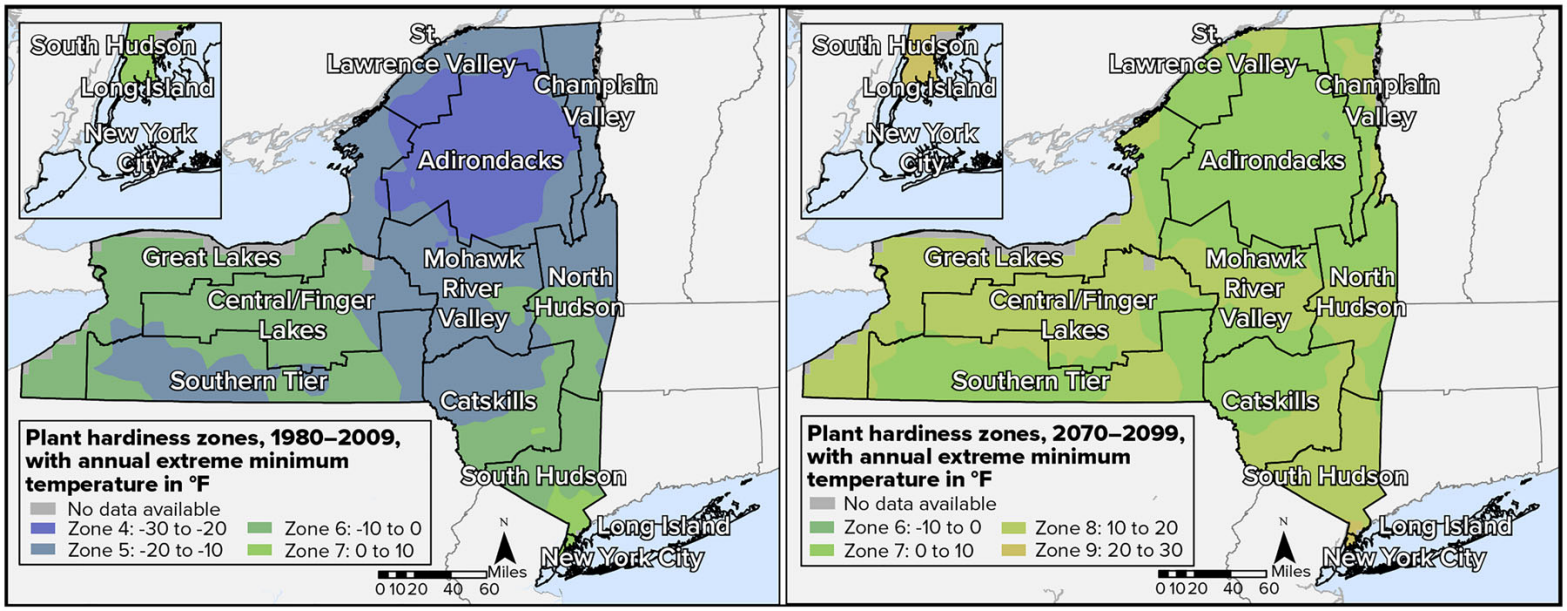
Projected changes in plant hardiness zones, 1980–2009 to 2070–2099. These projections use the SSP5‐8.5 greenhouse gas emissions scenario. Note that this is not the current official USDA Plant Hardiness Zone Map, which was updated in 2023 to reflect a newer baseline period and does not include projections. Adapted from a separate supplemental analysis by USDA, Office of Sustainability and Climate (2022).^67^ Data from U.S. Forest Service (2018).^69^

Soils are also highly diverse across the state due in part to the differences in climate, geology, and topography.^68^ Sandy coarse‐textured soils dominate certain growing regions, while clayey fine‐textured soils are prevalent in other parts. This soil diversity also has implications for the storage of soil carbon, both for climate mitigation strategies (e.g., carbon sequestration) and adaptation strategies (e.g., building organic matter for greater water retention). Further, these distinct soil types require different management strategies, such as the need for tile drainage versus irrigation, with the health of agricultural soils directly related to crop productivity, farm sustainability, and the future adaptation potential of farms as agricultural regions shift because of climate change.^68^

### 3.3 Observed and projected impacts and adaptation strategies by commodity

The physical hazards discussed above will affect different commodities in different ways. Specifically, changes in temperature and precipitation will affect crop and livestock production differently across the United States.^70^ Understanding the commodity‐specific impacts, and potential adaptations to address those impacts, will help raise awareness about the steps needed to ensure that New York's agricultural operations become more resilient. Further, the adaptation practices and strategies implemented on an individual farm are not going to be “one‐size‐fits‐all” and must be tailored to specific farm production systems.^71^, ^72^, ^73^ The sections below highlight the impacts of climate change on key agricultural commodities produced in New York State: field crops, perennial fruit crops, vegetables and horticulture crops, dairy and livestock, agroforestry and maple products, and aquaculture. They also discuss practices and strategies that farmers can use to adapt to climate change in each commodity.

#### 3.3.1 Field crops

Field crops, also referred to as row or agronomic crops, include corn, soybeans, wheat, hay, and forage. These crops are particularly vulnerable to drought, extreme precipitation, and increases in weed, disease, and insect pressures because they are typically produced on tight margins with narrow profitability. They cover most of New York's agricultural acreage, mainly to support the state's dairy industry. Climate hazards that result in an increased need for management efforts, such as the use of pesticide inputs to manage emerging pests, can have negative impacts on the sustainability and cost of field crop production, and farmers may pass on cost increases to consumers.^74^, ^75^, ^76^, ^77^, ^78^

##### 3.3.1.1 Observed and projected impacts

Specific climate impacts to field crops include those described below:

- **Pests**. The pending threats from existing newly invasive pests are concerning and are already measurable in this sector. Herbicide‐resistant weeds are not a new problem, but a new spectrum of weed species that have developed resistance to many commonly used herbicides has recently been identified for the first time in New York State. Of particular concern was the 2019 identification of Palmer amaranth, considered the most destructive and difficult‐to‐manage weed in all field crop production in the United States.^79^ As regional climate changes facilitate the expansion and reproduction of invasive and resistant weed species, farmers will struggle from a lack of management tools, potentially leading to greater herbicide use. This, in turn, might contribute to the development of greater herbicide resistance.^80^
- **Outbreaks and epidemics**. Field crop diseases have become more prevalent and impactful to yield in recent years. Many corn farmers have long struggled with major outbreaks of foliar fungal diseases, including gray leaf spot, northern corn leaf blight, and common rust. They have also struggled with ear and stalk rot diseases that produce mycotoxins, which threaten the safety and marketability of forage and grain crops.^81^, ^82^ As the climate becomes warmer and more humid, the state's corn production could become more susceptible to disease epidemics, yield losses, and mycotoxin risks. Similar diseases and resulting mycotoxins threaten wheat and small grain production, and exceeding safe mycotoxin thresholds would make crops unmarketable.^83^ Soybean production has increased dramatically over the past 40 years in New York.^84^ As production has expanded, so has the number of associated pests and diseases, most of which were not known to exist in this cold northern region until recent years. Between 2013 and 2018, experts confirmed three major diseases and a nematode for the first time in New York State; the nematode is considered the most significant pest of soybeans globally.^85^, ^86^, ^87^, ^88^ According to Cornell University's soybean disease survey results for the years 2012−2018, these diseases and nematodes have expanded throughout the state.^89^
- **Excess soil moisture**. A higher frequency of surface ponding on fields due to the predicted increase in major storms means that field crop farmers will struggle to access flooded or waterlogged fields for timely planting, fertilizer and pesticide applications, manure spreading, and harvest. Farmers are often forced to choose between driving on soils that are too wet (which can cause rutting and compaction) or waiting to do their work when the timing is less than ideal (which can result in vast acreage going unplanted in spring or unharvested in fall). Weather fluctuations in recent years have illustrated these challenges. For example, in the spring of 2019, thousands of acres of productive farmland across the state could not be planted due to surface ponding or flooding. That excessively wet year was followed by an excessively dry spring of 2020, when many acres of corn experienced insufficient weed control due to a lack of minimum precipitation necessary to activate critical herbicide applications (Field Crops Expert [2022, June, Personal communication]). Those back‐to‐back years of conflicting water stress scenarios placed significant stress on New York's field crop farmers. It is important to note that unlike high‐value specialty crops, such as fruits and vegetables, field crops such as corn, soybeans, wheat, and hay are generally not economical to irrigate in New York. When drought scenarios occur, field crop farmers are at the mercy of the weather to produce a viable crop, just as they are when their fields flood from untimely or excessive rainfall events.
- **Heat stress**. Increases in soil and air temperatures beyond a threshold level can cause permanent harm to plant growth and rates of development, which can affect crop yields.^90^ Elevated nighttime temperatures that increase plant respiration rates reduce plant carbon fixation and biomass accumulation.^91^ While crop sensitivity to heat stress varies depending on duration, crop type, and temperature,^92^ heat stress can cause detrimental effects to field crops in several ways.^93^ Rising temperatures and heat stress can cause changes in the morphology, physiology, and growing periods of crops, resulting in lower yields.^92^ High heat can reduce pollination and grain yield in corn and other crops, which will also result in significantly lower yields.^94^
- **Shifts in growing seasons and regions**. The growing regions of crops in the United States are shifting in response to climate change.^95^ Studies suggest that changing climatic conditions will substantially impact several field crops, including corn. Forecast models developed for the Northeast project that corn will experience a faster rate of growing degree‐day accumulation with a reduction in time required to reach maturity as a result of fewer spring and fall freezes, as well as an increased frequency of days with maximum temperatures meeting or exceeding 35°C during key growth stages, while at the same time experiencing greater water deficit during reproductive stages.^96^ Data on rainfed corn are widely available, and projections suggest that some parts of the United States will experience an increase in yield of 5% to greater than 25%, while other areas will experience a decrease of 5−25%.^97^, ^98^

##### 3.3.1.2 Adaptation strategies

The following adaptation strategies can help field crop farmers address climate impacts:

- **Improved soil drainage**. Farmers can reduce flooding and excessive soil moisture due to extreme weather events by improving soil drainage. This may involve expensive installation of tile drainage (subsurface) systems to physically transport water from fields. This practice occurs in parts of the state that have finer clay‐textured soils with inherently poor drainage. One team of researchers developed a model that shows that the impacts of climate change (specifically an increase in precipitation) differ on farmland that has been tile drained versus not tile drained.^99^ The model supports the idea that the value of these land types differs because farmland that has not been tile drained (e.g., sandy soils) could benefit from increased precipitation (e.g., water availability to crops). There has been an increase in the number of acres using tile drainage across the state, from 780,996 acres in 2012 to 861,265 acres in 2017.^14^
- **Improved soil health**. Another adaptation strategy for New York State field crop growers is improving soil health via the adoption of sustainable soil management practices, which also provide many other environmental benefits. Some soil management practices include reducing tillage, planting cover crops, leaving crop residue on the field, applying manure and other organic amendments to build organic matter, and avoiding activities that limit water infiltration.^100^ Reducing tillage and planting cover crops can also enhance moisture retention in soils during periods of drought, improve water infiltration during heavy rain events, alleviate compaction, and improve crop yields over the long term.^101^ These practices will contribute to improved access to fields for timely planting and harvest. Although there are no economically feasible management practices for heat stress in field crops, moisture management contributes to alleviating drought stress.^102^, ^103^ Section 5.3 provides additional information about soil health and its multitude of benefits.
- **Improved crop varieties**. Breeding programs can select for crop varieties that are more heat‐tolerant, with improved drought resistance, and that could be adapted to production in New York State.^104^ Farmers might also shift to growing longer‐season varieties in response to a lengthening growing season.
- **Integrated pest management (IPM)**. The difficulty of managing weeds, insects, and diseases under climate change conditions is quickly becoming a top concern for field crop growers.^105^ An increase in the frequency and severity of outbreaks, or in the number of generations per year, will challenge growers and increase production costs.^106^ As a result, it is important for growers to have a sound IPM program in place. Demand will increase for crop varieties that offer protection via improved resistance, and the need for other IPM tools will also increase. Models (such as those offered by the Network for Environment and Weather Applications) and pest/disease management calendars that growers have long relied upon to predict outbreaks will need to be updated to align with changing weather patterns.^107^
- **On‐farm ecology**. Practices that rely on sound on‐farm ecological principles can also help reduce the impacts of climate change and dependence on synthetic fertilizers and pesticides. For example, the practices of double cropping and intercropping are a way to expand production, diversify the agricultural landscape to increase crop efficiency and resilience against pests, enhance farm biodiversity, improve soil health, and provide biological pest control.^108^ Diversifying crops across temporal and spatial scales on the landscape and rotating crops can help foster greater biodiversity, preventing pest populations from building up and ensuring production stability.^100^ Farmers will require additional resources and training on updated management practices for new and expanding pest and disease threats. Crop consultants and extension specialists will need to be diligent in monitoring efforts and changes to management recommendations.^107^ It will be critical that farmers have access to an arsenal of tools to manage the increased threats from pests and diseases, including cultural, mechanical, technological, biological, and chemical options.

#### 3.3.2 Perennial fruit crops

New York's fruit crops include apples, grapes, peaches, and cane or bush berries. The state is well known for many of these crops, and it ranks seventh nationwide in the production of fruits, tree nuts, and berries.^14^ Vineyards, wineries, hops farms, farm breweries, and hard cider production are an increasingly important part of the agricultural economy in New York State, even if not fully accounted for in the last USDA Census of Agriculture. According to the New York Wine and Grape Foundation, there are approximately 35,000 vineyard acres and 471 wineries throughout the state; the wine industry generates $6.65 billion in economic benefits annually.^109^ In 2022, New York ranked third nationally for most wine produced, and fourth (behind California, Texas, and Florida) in terms of economic impact.^110^ With 126 cideries operating, New York has more hard cider (i.e., fermented apple juice) producers than any other state. Hard cider producers generate $1.7 billion in total economic benefits to the state, contribute $378 million in taxes, and employ nearly 6150 people, who are paid $520 million in wages.^111^ Many of these cider producers operate under a farm cider license, which requires the exclusive use of New York–grown apples. Hop production (included in this section because of its perennial nature) has also made a resurgence in the state over the last decade to support the booming craft beer industry,^112^ as the number of breweries in the state grew from 95 in 2012 to 504 in 2022, ranking New York third in the nation for the production of craft beer, with an economic impact of approximately $5.4 billion.^113^, ^114^ New York passed a farm brewing law in 2012 that requires any beer labeled as a New York State beer to have 60% of its hops and other ingredients grown or produced in the state; this percentage increased to 90% in January 2024.^113^ New York now ranks approximately sixth among states in growing hops,^115^ with 145 farm operations growing 322 acres of hops in 2017.^14^

Many of New York's fruit crops are long‐lived perennials. For example, apple trees typically last 20 or more years, grape vines 25 or more years, and cane berry plants 10 or more years. Perennial fruit crop plantings also require significant capital resources. For example, high‐density apple orchards and vineyards with trellis systems, irrigation, subsurface drainage, and plants cost up to $25,000 per acre.^116^, ^117^

The majority of the perennial fruit crops grown in New York State are spring‐blooming deciduous plants, including apples, grapes, peaches, and cane berries. These species evolved a strategy to stay dormant during the winter when cold temperatures and low light levels make growth difficult. Emerging from winter dormancy into spring growth requires a two‐step process of first accumulating sufficient winter chill to alleviate the plant from endodormancy, and then accumulating sufficient heat to alleviate the plant from ecodormancy and start the process of bud break.^118^ Ecophysiologists have created several models to calculate how long plants need to stay in endodormancy before they can start growing. The North Carolina model is most commonly used in New York State and the region.^119^ This model calculates chill units as the number of hours between 32°F and 45°F. Once the plant has obtained sufficient chill units, it moves into a state of ecodormancy. For the plant to end ecodormancy and start growing requires the accumulation of heat units, called growing degree‐days. Both endo‐ and ecodormancy requirements vary by species and cultivar.

These attributes create challenges. For example, while annual crop producers can change cultivars and even species from one year to the next, perennial fruit crop producers manage the same planting for many years. Adding to the challenge of managing fruit crops in a changing climate is that some crops, such as apples and grapes, have established markets for specific cultivars (e.g., Gala apple). Thus, changing to a new cultivar would require significant consumer marketing. For example, hybrid grapes might be more resilient to climate change because they have genes from North American species, but consumers have been reluctant to purchase wines made from hybrid grapes over well‐known *Vitis vinifera* cultivars (e.g., Chardonnay).^120^, ^121^

Value‐added craft beverage industries will undoubtedly feel the effects of climate change, with reduced crop yields and compromised quality. There is limited information about how climate change might affect hop production in the region, though, so this potentially high‐value specialty crop will not be discussed in detail.

##### 3.3.2.1 Observed and projected impacts

Impacts specific to fruit crops include the following:

- **Earlier budbreak**. Current climate projections suggest that meeting endodormancy requirements for most of the commercially important apple cultivars will not be problematic in New York State through the end of the century.^40^ However, warmer spring temperatures are projected to cause earlier budbreak. Spring frosts make early budbreak a major concern for perennial fruit crops. These crops develop their flower buds the previous summer and only produce one crop per year. When there is an early budbreak in the spring, late spring frost events can result in damage to the flowers and thus crop failure. In fact, this has already occurred several times since 2010 in New York—for example, in 2012, when a week of temperatures above 70°F in early March led to budbreak 3−4 weeks earlier than the historical average.^122^, ^123^, ^124^ This early budbreak was then followed by several frost events. In 2012, 40% of New York's Concord grape vineyards had freeze injury, which resulted in a 30% crop loss and economic losses of about $45–60 million.^125^ Similarly, New York orchards lost about half the apple crop to the spring frosts in 2012, costing growers millions of dollars.^8^ Individual apple growers reported economic losses of up to 80%, with one grower in Onondaga County losing between $500,000 and $1 million due to the frosts.^123^ The 2012 spring frosts affected peaches, cherries, nectarines, and other fruit crops as well.^123^ Additionally, when fruit plants bloom too early, their flowering becomes less synchronized, meaning that cross‐pollination, which is required by many species for fruit set, might not occur. Another concern related to early budbreak is that insect pollinators, such as European honeybees, are less likely to be active in early spring, which could result in poor pollination and low fruit set.^126^
- **Physiological disorders**. During the growing season, many fruit crops are susceptible to physiological disorders such as bitter pit and sunscald from elevated heat.^127^, ^128^ Warmer temperatures will also result in greater plant transpiration, potentially leading to increased irrigation needs. These needs will be especially pronounced for most of the established apple plantings (i.e., trees planted in the past 20 years),^128^ which use dwarfing rootstocks. These trees have sparse root systems and are less efficient at exploring deeper into the soil profile for water and nutrients and may experience more pronounced physiological disorders.
- **Pests**. Fruit crop growers regularly manage large complexes of diseases and arthropod pests, both endemic and invasive. For example, the bacterial pathogen fire blight (*Erwinia amylovora*) is a constant threat in orchards across the Northeast, including in New York.^129^ However, these pests could benefit from a changing climate (refer to Section 3.4.2). Some insect pests will produce more generations per year, thus resulting in more damage to plants and/or necessitating more pesticide applications, including applications closer to harvest.^130^ The goal of IPM over the past 50 years has been to reduce pesticide use,^131^ particularly chemistries with the greatest negative impacts on human health and the ecosystem. Climate change will potentially cause fruit growers to reverse this trend because they will need to apply more pesticides.
- **Harvest‐related concerns and disorders**. As daytime temperatures increase, harvesting fruit will be more physically detrimental for farmworkers.^132^ Fruit will also have more field heat at harvest, which will require the use of precooling systems to ensure adequate shelf‐life.^133^ Many specialty crops are perishable, with short postharvest storage lives. Fruits that can be stored (such as apples and pears) require energy‐intensive refrigeration, which will get more expensive with warmer temperatures and rising energy costs. Also, physiological storage disorders (such as scald and browning) are exacerbated by warm temperatures in the weeks prior to harvest and during harvest. Heavy rain events are increasingly problematic for soft fruit like berries, grapes, and cherries.

##### 3.3.2.2 Adaptation strategies

Perennial fruit growers can take several steps to adapt to a changing climate. Examples include:

- **New cultivars**. To some extent, growers can experiment with new plant cultivars that are more resilient, but this must also be accompanied by consumer acceptance.^134^ Switching cultivars on a perennial fruit farm is much more challenging than for annual crops, given the long timeframes and expenses required.^116^, ^117^ Growers are exploring alternative fruit crops, such as paw paws and persimmons, but currently, these fruits have very small niche markets.^135^ Additionally, changing to later‐blooming cultivars is possible but can potentially be problematic for controlling diseases such as *E. amylovora*, the bacteria that causes fire blight, a devastating and often lethal disease of apple and pear trees. Some northern berry fruit like haskaps and saskatoons are being grown farther south, as they have great winter hardiness, but they often break bud too early in the season and are prone to spring frost damage.
- **Cooling and air mixing**. To reduce physiological disorders, growers in warmer climates already use netting, high and low tunnels, and overhead evaporative cooling systems to reduce heat stress–related impacts on fruit crops. New York growers might need to follow suit. Growers can also take steps to reduce frost damage resulting from early season budbreak. Strategies include air mixing with wind machines (large propeller blades mounted on tall shafts), the deployment of helicopters, and fabric orchard covers that cost thousands of dollars per acre. Climate‐smart decision‐support tools, such as an Apple Stage/Freeze Damage Probability tool and Grape Hardiness and Freeze Risk tool, are available to assist growers in mitigating weather risks.^136^ Spun‐bonded row covering is used regularly for frost protection in strawberries, along with overhead irrigation. Adding water to crops in the spring exacerbates soil‐borne disease pressure, so alternatives to overhead irrigation are increasingly sought.
- **Water management**. Increasingly, fruit growers are installing tile drainage to address flooding in years of extreme precipitation, as well as irrigation for when conditions turn dry. Fruit growers in New York have not historically used either of these practices, but many now recognize these approaches as essential for successful fruit production.^46^ Increasing irrigation capacity requires the use of surface water (e.g., lakes, streams, ponds) or groundwater, which can tax hydrological systems and the organisms they support (refer to the Water Resources chapter). Some fruit growers use municipal water, but this option can be expensive, and in drought years, water providers may need to prioritize domestic and nonagricultural commercial uses. Protective rain‐shedding materials also help with water management.

Refer to the Apple Production Threats and Adaptation case study for more information on adaptation options for apple growers.

#### 3.3.3 Vegetables and annual horticulture crops

New York boasts a tremendous diversity of vegetable and annual specialty crop (e.g., floriculture, sod, and greenhouse) production. Vegetable production includes crops grown both for fresh market and processing across the state. The state's production of cabbage, squash, peas, peppers, and pumpkins ranks within the top 10 nationally. Vegetable, melon, and potato sales contributed nearly $379 million to the state's economy, according to the last Census of Agriculture.^14^ New York ranked ninth nationally in 2021 in terms of floriculture sales, with $181 million in sales from 600 operations.^137^

The climate vulnerabilities affecting vegetable and horticulture crop production in New York State are both biotic and abiotic. These vulnerabilities relate to changes in precipitation, higher and more variable temperatures, and increasing pest (insect, mite, weed, and disease) pressures. While higher temperatures will extend growing seasons and be beneficial for some vegetable crops, others will suffer.

##### 3.3.3.1 Observed and projected impacts

Water is one of the most important determinants of yield in crop production.^138^ If, as projected, New York experiences more variable precipitation and longer periods of drought during the peak growing season (summer), the result will be increased water stress for producers.^1^, ^139^ While many of the state's vegetable producers have irrigation infrastructure, not all will have the capacity to maintain adequate water supplies during extended periods of drought.^36^ Conversely, excessive rainfall can also reduce yields, physically damage crops, cause flooding of fields, increase losses due to disease and weed competition, and delay planting and harvesting. More frequent flooding has already forced farmers to rethink where and what types of crops to plant in certain fields.^140^ Higher temperatures will further exacerbate the impacts of more variable precipitation due to the altered evaporative demand of crops.^141^

Most vegetable crops are susceptible to heat stress at particular stages of growth and development during the growing season, and this stress eventually can lead to a reduction in yield.^142^ A crop's temperature requirements for optimum growth determine whether it is categorized as a hot‐season crop (77–80.6°F), a warm‐season crop (68–77°F), or a cold‐season crop (64.4–77°F).^143^ Early‐season warm periods followed by cold spells can damage young seedlings and cause cold‐season crops to set heads when they are too young to support them. Under these conditions, plants are stressed because spring plantings are not acclimated to heat, and warm‐season crops are not acclimated to sudden cold night temperatures. Unusually warm weather during portions of the growing season that are normally cool to mild can increase plant stress and reduce crop cold tolerance while also promoting the development of diseases typically associated with warmer months. The associated unpredictability of when certain diseases will be active in their fields, along with abnormal temporal overlaps in disease presence, can leave growers struggling to manage their crops.^144^ Higher average temperatures during the growing season typically cause plants to use more energy for respiration (i.e., maintenance) and less for growth. This will not only impact yield, but also reduce the marketability of fruits and vegetables. As more energy gets allocated to vegetative growth instead of to the harvested product,^93^ color, size, and flavor can all be affected. This effect is expected to worsen as temperatures rise, particularly if heat stress occurs during fruit maturation.^145^ Some heat‐ and weather‐induced impacts observed in vegetable crops include bract development (i.e., development of small green leaves in the head), uneven head development, stunting, discoloration, bolting (i.e., flowering), reduced quality (e.g., bitter flavor), fruit drop, cracking, and withering.^142^ Growing common vegetables, such as potatoes, cabbage, and snap beans, will be more difficult as climate conditions change because of heat‐related stress and prolonged periods of high temperatures.^9^, ^142^ However, an extended growing season might benefit the production of some longer‐season crops such as watermelon, tomato, pepper, and cantaloupe.^1^ Some growers could, therefore, change what they grow.^9^

Crops weakened by the impacts of water and temperature stresses will become more vulnerable to indirect stresses and more attractive to pests (weeds, diseases, and insects).^145^ For example, sweet corn is expected to become increasingly vulnerable to corn earworm and Stewart's wilt, a bacterial disease caused by flea beetles.^1^, ^36^ Pests such as the potato leafhopper, which feeds on a wide variety of crops including potatoes, green beans, and flowers, are also appearing sooner in the growing season^146^ and shifting into regions outside the distribution of their natural enemies. The effectiveness of biocontrols and other IPM strategies could decrease as new species invade, and pesticide use could increase. The influence higher temperatures and greater precipitation have on plant physiology is also expected to impact crop and weed competition.^147^

##### 3.3.3.2 Adaptation strategies

Vegetable and annual specialty crop growers can take several steps to adapt to a changing climate. Examples include:

- **Improved water management**. For vegetables and horticultural crops, both too much and too little water can negatively affect productivity. Increased irrigation requires access to surface water or groundwater, requires energy to pump this water, and imposes an environmental cost by removing water from natural systems. However, irrigation technologies and best management practices have evolved over the past several decades. Today, best management practices focus on overall water management and include improved conveyance, irrigation scheduling, and application methods.^148^ Proper water management promotes maximum plant growth while minimizing erosion and pollution and optimizing water supplies. Shifting from overhead irrigation methods to drip, subsurface, or microirrigation maximizes the amount of water entering the crop root zone and helps offset some of the negative impacts of prolonged dry periods while not stressing water resources. These methods also reduce the risk of disease by not wetting crop leaves, because leaf wetness duration is an important factor in the extent of plant diseases.^16^ However, shifting to such methods is not possible on all farms, especially for larger operations. Other adaptation strategies for water management include installation of tile drainage (where applicable), erosion‐control measures, and capture of surface water runoff and tile drain output following large rain events into water retention or infiltration infrastructure. With climate models predicting more extreme weather events, improving drainage and increasing irrigation will become more necessary. Additionally, installing technologies such as soil moisture sensors and crop water‐use sensors where possible can improve monitoring and enable site‐specific management.^149^
- **Shifting planting dates**. Vegetable farmers might consider shifting planting dates to alleviate climate‐related stressors such as heat stress during critical growth periods. However, ideal planting dates each season are unpredictable.^36^ Farmers in some parts of the state might need to stop growing certain crops or switch to more resilient crops (e.g., shift from vegetable production to field crops and small grains). There are many useful online decision‐support tools that growers can use to make informed decisions about their production systems at the field level based on location‐specific climate data, weather forecasts, and future outlooks. For example, the water deficit calculator estimates soil water content within a crop's root zone based on the soil and crop type, growth stage, and irrigation parameters. It helps inform farmers and irrigation managers about current and forecasted water deficits and helps them determine the optimal frequency and duration of watering to avoid plant stress.
- **Diverse cropping systems**. Planting several different crop species or varieties in different spatial scales (i.e., areas on a farm) and time scales (e.g., spring vs. fall) can help producers better cope with potential crop losses.^141^ However, implementing diverse cropping systems on farms is difficult due to economic pressures, the need to rotate crops to control diseases and pests, and the need to supply a consistent amount of product to retain market share (Buck E, Cornell Cooperative Extension Vegetable Program [2022, July, Personal communication]).
- **New varieties**. Vegetable breeding programs are developing new varieties that have greater heat and drought tolerance and water‐use efficiency compared with currently grown varieties.^142^ Field trials are ongoing in New York to evaluate the success of these breeding efforts.^150^
- **Soil health practices**. Implementing practices that build soil health will continue to be critical for both small and large farms to increase resilience to drought, excess rainfall, and pest pressure. Using IPM strategies will be key to helping farmers manage intensified pest, weed, and disease pressures.^36^ Investing in soil health will also help farmers withstand climate change impacts overall.
- **Protective and controlled‐environment equipment**. Many vegetable farmers have turned to using protective structures, such as hoop houses and high tunnels, as a strategy to cope with extreme weather and pest invasions.^151^ Additionally, both urban and rural specialty crop growers are increasingly using vertical farming and controlled‐environment agriculture.^152^ Through this approach, not only are plants grown in protective structures, but growers also control the environmental conditions, including temperature, humidity, and light. Controlled‐environment agriculture has already been widely adopted among both urban farmers and peri‐urban farmers (i.e., those working land on the edge of cities) because it offers the opportunity to grow quick‐turnover fresh produce crops in places where land is limited, rent is high, and food is not typically grown in large quantities (e.g., New York City). These controlled systems can increase food security by providing access and availability while minimizing the effects of climate change–induced erratic weather. Also, because controlled systems reduce the risk that crops will be contaminated with pathogens, vegetable growers can select crop varieties bred for flavor as opposed to heat tolerance, disease resistance, and long‐distance transport. In addition, in a controlled environment, producers can grow more food with less water, fertilizer, and pesticides.^152^ It is important to note that there is an environmental and greenhouse gas cost to building and operating these climate‐controlled systems. Not all crops are suitable to grow in these environments, and yields may not be equivalent to field‐grown crops. Another approach includes growing crops under solar panels (agrivoltaics); studies have demonstrated that crops grown under solar panels are protected from excessive heat, light, and evaporation.^153^, ^154^

#### 3.3.4 Dairy, livestock, and livestock products

Dairy farming is a major component of New York State agriculture. The dairy industry is the state's largest agriculture sector and accounts for almost half of the state's total income from agriculture.^155^ The state ranked fifth nationally in milk production in 2021 and 2022.^156^ In 2020, the state's dairy producers marketed over 15 billion pounds of milk, produced by about 625,000 dairy cows on roughly 3600 dairy farms.^155^ A 2021 report determined that the value of the state's annual dairy industry production output (direct, indirect, and induced) was $4.9 billion.^157^

As shown in Table 3-3, New York's dairy farms vary tremendously in size. According to the 2017 USDA Census of Agriculture, 18% have fewer than 10 cows, while 3.1% (142 farms) have over 1000 cows. The majority (52% or 2426 farms) milk 20−99 cows.^14^ Farms with a herd size from 50 to 199 cows produce 23% of the total milk statewide, while larger farms with over 1000 cows produce 40% of the milk.

**TABLE 3-3:** Dairy farms, dairy cows, and milk production in New York State in 2017, by farm size.

Herd size	Number of operations	Percent of operations	Number of dairy cows	Percent of dairy cows	Milk production^a^ (millions of pounds)	Percent of milk production
1–9 cows	844	18.16	2290	0.36	55	0.36
10–19 cows	364	7.83	5276	0.84	126	0.84
20–49 cows	1131	24.33	39,904	6.35	955	6.35
50–99 cows	1295	27.86	85,167	13.56	2038	13.56
100–199 cows	453	9.75	60,673	9.66	1452	9.66
200–499 cows	278	5.98	84,429	13.44	2020	13.44
500–999 cows	141	3.03	97,715	15.55	2338	15.55
1000–2499 cows	119	2.56	176,428	28.08	4221	28.08
2500–4999 cows	23	0.49	76,363	12.15	1827	12.15

*Note*: Data from USDA, National Agricultural Statistics Service (2022).^158^

^a^ Milk production data calculated using average annual milk per cow of 23,925 pounds for New York in 2017.

However, due to a variety of social, political, and economic factors in New York and nationally, there is a trend toward consolidation of smaller dairy farms into larger farms, with the number of farms with at least 1000 milk cows doubling from 2007 to 2017 in the eastern dairy states, which includes New York.^159^ In addition, available data from USDA on the number of farms and farm sizes do not necessarily reflect current conditions, as the last published USDA Census of Agriculture data are from 2017, and the 2022 Census of Agriculture data will not be published until 2024.^160^ Over the past 3−4 years, many small dairy farms have sold their cows and quit dairying. At the same time, the largest dairy farms have gotten larger, shifting the demographics of New York's dairy industry to a smaller number of larger farms overall.

New York provides optimal conditions for milk production for several reasons. The state is less hot than many other parts of the country, making it a better region for cow comfort. As a result, the state's cows produce more milk per unit of input than cows in many other regions. Water availability is less of a concern than in other regions of the United States, and high‐quality forages are grown in the state. New York's dairy farmers also have access to large markets across the East Coast. Several large, well‐known companies depend on the milk produced in New York, including Ben and Jerry's, Chobani, Kraft, Upstate, Stewarts, and Cayuga Milk Ingredients. Reduced yield from dairy farms due to climate impacts will affect individual farmers as well as these companies (Dairy Industry Expert [2022, July, Personal communication]). Because greenhouse gas emissions are a global concern and New York represents a good region for dairy cows, wise policies could support synergies between adaptation and mitigation to support the sustainability of the state's dairy farms (Dairy Industry Expert [2022, July, Personal communication]).

In addition to dairy, other livestock and livestock products are important to New York agriculture. A 2021 report from the Cornell Dyson School of Applied Economics and Management calculated the state's annual industry production output for beef, poultry, and other animals at $783 million.^157^ In 2021, New York State farmers produced beef cattle (over 100,000 head), poultry (over 1.7 billion eggs produced), sheep and lambs (∼80,000 head), goats (∼30,000 head), and hogs (∼61,000 head).^158^ Production in the dairy and livestock sectors combined (through direct, indirect, and induced effects) supported over 25,000 jobs.^157^ Additionally, the state's equine industry and related fields (horse training, facilities, veterinary care) account for more than $2 billion of value generated annually, and the industry supports over 30,000 full‐time jobs.^161^

Most dairy and livestock farms in New York still rely on their own ability to grow, manage, harvest, and store forages and some feeds to feed their animals year‐round; only a small percentage of forages/feed is purchased. Nationally, consolidation has led to changes in the relationship between livestock and forages on larger farms; cows are less likely to be grazed, and more likely to be confined and fed purchased feed (O'Neil K, Cornell Cooperative Extension North Country Regional Ag Team [2022, June, Personal communication]). Due to spatial constraints, only the smallest dairy and livestock farms can feasibly graze their animals on the farm. Those that can do so only require stored forages for winter/cold weather portions of the year. Others grow forages and feeds on their land instead of grazing their animals directly. This means that climate impacts to field crops, and adaptations to those impacts, are directly connected and critically important for these farms.

1 A huge challenge is the wet springs that can delay planting of forage crops, followed by very dry summers, where there is insufficient moisture for crop production, like the situation in much of New York State this summer.

—Dairy industry expert (July 2022)

Climate impacts affect all components of the animal agriculture industry, and these impacts will continue in the future. Livestock production faces significant and multifaceted challenges related to a changing climate. Maintaining and increasing current production levels will depend on implementing significant adaption strategies.

##### 3.3.4.1 Observed and projected impacts

A changing climate will challenge livestock producers to adapt while also meeting growing consumer demand for livestock products produced using sustainable practices. While global analyses project that climate impacts on the livestock industry will be most severe in tropical and subtropical areas, research indicates that even episodic heat stress (short‐term, moderate) can have negative impacts; therefore, New York State's animal agriculture sector must prepare for such changes.^162^

- **Heat stress**. Heat stress in livestock occurs when these animals generate and absorb more heat than they can expel through increased respiration and sweating or through supplemental cooling.^163^ It can negatively affect cow comfort, as well as reproduction and health, and is already having an observed impact on livestock.^164^ Heat stress will continue to lead to loss of productivity, including in New York.^165^ Of the many factors that affect livestock production, heat stress is considered a major factor that can lead to reduced productivity as livestock animals expend resources to adapt their physiology to increased temperatures.^166^ Similar trends in reduced productivity are apparent across livestock species, and it is likely that livestock producers will encounter increased economic losses due to animal stress and disease related to their heat tolerance.^167^ Cows respond negatively to both heat and humidity levels as heat increases. This relationship can be expressed in terms of the Temperature–Humidity Index (THI), a combined measure of temperature and relative humidity. Another useful measure is the Black Globe Humidity Index, which combines black globe temperature and humidity (Dairy Industry Expert [2022, July, Personal communication]). These indices focus on environmental conditions and do not consider the animal's own physiological responses to temperature.^168^ Longer‐term conditions can be examined in terms of environmental stress days—a measure like growing degree‐day units for crops, expressed as a running average value calculated using 5 years of data.
  Cattle in New York State have experienced environmental stress for decades. However, the warming climate is increasing the total time cows experience heat stress (Dairy Industry Expert [2022, July, Personal communication]). New York's dairy farming may be affected, as negative impacts of heat stress appear when the THI is as low as 68.^169^ The immediate effects of heat stress in dairy cattle include a reduction in feed intake, increase in water intake, reduction in lying time, and reduction in milk production. Multiday periods of heat stress can be especially detrimental, particularly when combined with warm nights. Specifically, ruminant livestock can reach a heat stress crisis with three consecutive nights above a THI value of 70.
  Longer‐term impacts of climate‐induced heat stress include compromised reproduction, increased lameness, and compromised immune function in dairy and other livestock.^170^, ^171^ For dairy, Cornell University researchers have demonstrated that enhanced gut permeability—which can result from heat stress—can trigger poor immune health and inefficient milk production.^172^ Compromised immune function may lead to reduced migration of lymphocytes to the udder, causing an increase in the number of cases of clinical and subclinical mastitis in lactating cattle. Cattle may also experience more bacterial infections due to lowered immune response associated with heat stress. Hyperthermia may cause an increased prevalence of liver lipidosis and metritis and lead to physiological and behavioral changes that cause reproductive issues with livestock species.^173^, ^174^, ^175^ Reproductive effects include the impairment of oocyte development, early embryonic death, deficient fetal growth, and diminished performance of the female during lactation, which may limit the development and growth potential of offspring.^175^ The impacts of heat stress on dairy cattle, and the corresponding economic losses for the farms, can last for years, with heat stress in the last gestational period having a negative impact on at least the next two generations of animals.^176^ Lethality of heat stress is also a concern.^177^
  Climate change and associated heat stress are also detrimental to the production of other livestock animals, including beef cattle, sheep, goats, poultry, swine, and horses. The health and performance of livestock animals and horses is linked to their comfort. It is broadly accepted that beef cattle are most comfortable with air temperatures between 32°F and 75°F. With the influence of climate change, livestock producers and veterinarians may encounter expansion of existing diseases, the emergence of new diseases, and increased parasitic loads or occurrences of parasitic infections.^178^ The incidence of vector‐transmitted disease (disease transmitted through flies, mites, mosquitoes, etc.) may also show a positive relationship with increased temperatures and precipitation.^179^

- **Feed sources and intake**. Literature indicates that feed sources for livestock production will be affected by increased temperatures, changing weather patterns, and (in other areas of the country) a lack of sufficient water resources to meet irrigation system needs.^180^ These impacts include a decline in pasture lands, deterioration of feed quality, altered patterns of grazing and changes to grazing systems and infrastructure, and overall reduced availability of forages, grains, and other feedstuffs.^180^
  Climate change could particularly affect traditional ensiled feeds and forages, as increased moisture will lead to an increase in molds and associated toxins. These impacts should be of special concern as ensiled feeds constitute a large percentage of feed used in the state's dairy cattle industry. Livestock producers should assume a loss of both feed quality and quantity because of climate change, which will require them to purchase additional feed and raise their costs of production.
  Farmers will likely need to adjust the diets of ruminant livestock (cattle, sheep, and goats) as the climate warms, as ruminant livestock generate body heat through rumination.^181^ This might involve changing the feed composition and the nutrient profile. Producers often find that ruminants increase their feed intake during cold weather, and this increased feed intake affects many productive factors associated with these livestock (e.g., progeny birthweight, average daily gain, and milk production). Thus, a warming climate, combined with a lack of supplemental cooling or other measures, will likely cause ruminant livestock to have reduced and more variable feed intake, resulting in reduced performance and yields.^182^

- **Water availability and quality**. Changing water availability and quality is also expected to affect animal agriculture. Farmers use ponds, streams, and groundwater for livestock production. It is widely anticipated that climate change will affect these water resources.^183^ Rising temperatures will also cause animals (especially production‐oriented ones) to increase water consumption.^180^ However, a lack of water availability is less of a concern in New York and the Northeast than in other regions of the United States.^184^
  Climate change and animal agriculture can also affect water quality due to increases in extreme precipitation events. Agricultural nonpoint source pollution can include runoff contaminated with pollutants from excess fertilizers and with bacteria and nutrients from livestock farms and faulty septic systems. This pollution, when combined with warmer water temperatures, can negatively affect the water quality of lakes and other water bodies,^185^ leading to an increase in HABs.^186^ Refer to the Water Resources chapter for more information on HABs. Nutrient inputs need to be carefully managed to minimize losses, and proper nutrient management should be combined with other practices aimed at reducing excessive runoff and soil erosion, which affect water quality.

##### 3.3.4.2 Adaptation strategies

Over the last few decades, livestock and dairy producers have made significant improvements to production efficiency and animal comfort through genetics and breeding programs, better nutrition, and improved housing facilities. Livestock producers will need to continue to adapt current production practices to provide conditions that alleviate heat stress and address other climate impacts. Some adaptation strategies will apply directly to animal health and productivity, others to production of feeds and forages, and others to farm infrastructure and systems (O'Neil K, Cornell Cooperative Extension North Country Regional Ag Team [2023, July 20, Personal communication]). While New York is largely invested in dairy farming, increased temperatures and other impacts of climate change may force livestock producers to diversify their farming operations. Adaptions include:

- **Adaptation to heat stress and supplemental cooling**. Heat stress adaptations, discussed further below, include providing additional natural or structural shade for animals; increasing ventilation and air speed; providing supplemental cooling through fans, cooled mats, or cold showers; expanding access to water resources; and selecting specific breeds or individuals (through genomic or phenotypic selection) that show tolerance for heat. Livestock and cow comfort is directly related to efficient production and is essential for optimizing an animal's quality of life and welfare. Many livestock producers already use best management practices such as soft bedding, appropriate stocking rates per the facility or pasture space, appropriate lighting, and protection from wind, rain, heat, and excessive sun exposure.^187^ For pastured livestock, increased shade can help to mitigate productivity losses due to a changing climate. However, because dairy cattle and other livestock are often raised in confinement and under highly controlled conditions, increased supplemental cooling will be needed. Generally, facilities for cattle, poultry, and horses will experience an increased need for fans, adaptive barn designs, and insulation due to a changing climate and more severe temperatures and weather patterns.^188^ However, heat abatement with fans and sprinklers will not completely prevent the economic losses linked to a heat stress event in dairy cattle.^165^ These technologies most often require fossil fuel use as well. Producers and consumers will likely feel the impact of these needs as increased production and retail costs, respectively.
- **Forages and feeds**. Because New York dairies and livestock farms are largely integrated with forage and feed production, these farmers will need to adopt adaptation practices to ensure that they have adequate forage and feed for their animals. These practices include improving soil drainage and soil health, using new crop varieties, IPM, and ecological management practices.^189^
- **Shaded grazing practices**. Two types of shaded grazing practices, silvopasture and solar panel grazing, are beneficial adaptation options to address heat stress in livestock.
  Silvopasture is the integration of wooded areas and grazing livestock. With appropriate design and observance of cooperation and competition between animals, woody plants, and forages, silvopasture can provide shade, diverse forages, and increased plant biodiversity.^190^ Silvopasture could become a sustainable agricultural practice in New York over the long term but would be difficult to implement at a scale that would meet the demand for raw products for dairy food products; focusing on silvopasture would require that the industry undergo tremendous changes to shift from the intensive dairy model that has been used in the state for decades (Dairy Industry Expert [2022, July, Personal communication]). Silvopasture could help smaller livestock producers adapt to climate change and could also help them market their products to customers focused on sustainable livestock production practices.
  Solar panel grazing has increased in popularity as nondairy livestock producers seek artificial sources of shade and untapped forage resources. Grazing sheep under solar panels and related infrastructure can reduce heat stress while encouraging the formation of additional energy resources,^191^ providing cobenefits for pollinators and wildlife, and increasing soil health.^192^ A 2020 study showed that, compared with other sources of shade that are generally available to livestock (trees, buildings, and structures), sheep preferred solar panels.^191^ As an alternative to mowing at solar installations, grazing livestock between rows of solar panels provides needed nutrients to the animals and maintenance to the site. Further, grazing at solar sites is expected to lower barriers for new farmers interested in beginning sheep farm enterprises and to help existing farmers expand because of the income generated from vegetation management and decreased need to rent land.^192^ While solar panel grazing is being used in other locations, it is not currently being implemented at most proposed or planned solar installations in New York State (O'Neil K, Cornell Cooperative Extension North Country Regional Ag Team [2023, July 20, Personal communication]).

- **Water quantity and quality**. Some facets of animal agriculture could expand in New York and the Northeast due to an uptick in temperature and water crises in other regions of the United States. Farmers and dairy food companies will look to relocate to regions where there are reliable water resources; New York could prepare itself to be the go‐to state (Dairy Industry Expert [2022, July, Personal communication]). However, even in New York, climate change brings the potential for less‐reliable water availability. Because of this, livestock producers should consider production strategies to make sure they have fresh, quality water available for their animals. Rainwater collection, treatment, and storage is one way that small and mid‐sized livestock producers can safeguard water resources for their livestock. While most rainwater‐harvesting systems are regarded as a backup water source, supplementing water from ponds, wells, and streams, they serve an important purpose.^193^ Components of such a system include catchment (generally a roof), conveyance (gutters or plumbing system), storage (tank or cistern), treatment (hardening/softening/removal of contaminants), and then distribution to the livestock.^193^
  Water resources are of major concern to dairying. The housing and other facilities of a modern dairy require flushing, disinfection, and a constant water supply for lactating cattle; thus, dairies require dramatically different water resources from other animal agriculture enterprises.^194^ Water recycling or “polishing” can lead to a more sustainable future.^195^ Recycling of wastewater, evaluating water runoff from buildings and concrete/cemented areas, and measuring unnecessary water use in the parlor and for irrigation can support future water availability.
  Many agricultural best management practices, if used properly, can help farmers prevent runoff and protect water quality. These practices may include the use of tools that help with the timing of manure spreading based on weather forecasts; use of erosion‐control systems; and planting of riparian buffers or grass filter strips.^196^ The New York State Agricultural Nonpoint Source Abatement and Control Program can provide cost‐share grants to assist farmers in adopting practices that will help protect water quality.^185^, ^197^

- **Manure management**. Animal agriculture in New York typically requires the storage of manure (primarily from dairy cattle, swine, and poultry, and to a much lesser extent from beef cattle). With climate change, livestock producers will be challenged to adapt production practices to better manage nutrient losses and the storage of manure resources. The use of impermeable covers and flares for long‐term manure storage may be important to reduce precipitation impacts and greenhouse gas emissions.^188^ Subsurface injection of manure and digestate could further improve nutrient management, reduce trucking and emissions, and produce even greater benefits if double‐cropped with corn silage and winter rye.^189^
  Adoption of manure‐based anaerobic digesters could help generate cleaner energy for use on the farm and in the surrounding community, while also helping to prevent nutrient runoff.^188^, ^198^ While anaerobic digesters are effective manure management tools to increase sustainability at the farm level, their capital costs remain high, making them an unrealistic option for most farms without significant subsidies or cost‐share programs. Federal and state renewable fuel incentives (e.g., from the U.S. Renewable Fuel Standard Program and the California and Oregon Low‐Carbon Fuel Standard programs) are helping to spark an increase in anaerobic digestion projects throughout the country.^199^ In New York State, there are approximately 30 on‐farm anaerobic digestor systems operating as of 2023,^186^, ^200^, ^201^ with several more under construction, mainly at dairies with more than 1000 cows. Given that dairies of this size account for 40% of the cows and milk production in New York, a substantial impact is possible. These systems, and other manure management practices, can deliver win‐win benefits—increasing resilience to climate impacts while at the same time reducing greenhouse gas emissions.

- **Changes to livestock breeds**. Some livestock producers might choose to adapt to changing climatic conditions by selecting different types of animals to raise. For instance, sheep producers who traditionally have raised fleeced breeds of sheep may choose to change to hair sheep varieties that offer more parasite resistance and are generally less labor‐intensive to raise. The “slick” Holstein cow has a slicker hair coat than traditional Holstein cows, which promotes greater resilience and temperature dissipation during extreme heat.^202^ Some livestock farmers could also use genetic and genomic selection in their herds and flocks to select animals that require less maintenance, feed, and other resources. Expanding these practices could produce more efficient livestock that require fewer resources in a changing climate.

Refer to the Adaptations on Two Dairy Farms case study for more information on dairy farm adaptation strategies.

#### 3.3.5 Agroforestry and maple products

Agroforestry is the intentional integration of agriculture and forestry practices to create more diverse, profitable, healthy, and sustainable systems. In New York, agroforestry practices include sustainable woodlot management and the use of woodlands for growing medicinal plants (e.g., American ginseng, black cohosh, and goldenseal), mushrooms, nuts, fruits, and ramps, and producing maple syrup and wood products.^203^ Agroforestry systems comprise several practices that producers can integrate into existing agricultural and forested lands including alley cropping, windbreaks, riparian forest buffers, silvopasture, and forest farming.^204^ These strategies can enhance farms’ resilience to climate impacts by helping manage the uncertainties and complexities of climate change^205^ through enhanced crop and livestock production, increased wildlife habitat, and diversified income streams.^204^

The remainder of the discussion below focuses on maple syrup because of its particularly notable contribution to New York's agricultural economy. (For a discussion of climate impacts on forestry products such as timber, refer to the Ecosystems chapter.) New York is the second largest maple‐producing state in the United States, behind only Vermont. Syrup is not only a source of income for the more than 2000 maple sugar makers across the state,^206^ but also a family tradition, a food source, a commodity to trade, and a medicine among Indigenous communities.^207^, ^208^ The maple industry is vulnerable to climate change, with potential impacts on forest type, tree health and vigor, and timing of sap flow.^209^, ^210^ Changing climatic conditions could also accelerate the growth of microbes in sap collection tubing.^211^

##### 3.3.5.1 Observed and projected impacts

As climate change brings warmer winter temperatures across much of the Northeast, the future of the sugar maple in northern hardwood forests is uncertain.^212^ Over the long term, temperature and precipitation changes associated with climate change are expected to shift the ranges of many tree species.^213^ In the nearer term, impacts to maple syrup production could relate to sap sugar content and sap flow—two factors sensitive to climate conditions. Sap sugar content is derived from carbohydrate storage; data support that the higher the temperature during the previous growing season (May–October), the lower the carbohydrate storage.^214^ Sap flow requires freeze‐thaw cycles, meaning that winter and early‐spring temperatures determine how well sap flows. Due to warmer winters, fewer freeze‐thaw cycles are occurring overall.^215^ Warmer winters have also shifted the timing of tree tapping to earlier in the year, from mid‐March to the beginning of February, and peak sap collection now occurs between the beginning of February and the end of March. Keeping tap holes open for a longer period makes proper maintenance to keep tap holes sanitary and avoid contamination even more important.^216^

Mean tapping season temperature is the best predictor of total sap volume produced during the season. One recent study projected that the midpoint of the tapping season will be nearly 1 month earlier by 2100 for the entire sugar maple production range, including across New York State, resulting in lower total sap collection.^214^ The same study projected that sap will have lower and more variable sugar content by the end of the century (lower by 0.55–0.65 °Brix, on average), that the optimal production range will shift northward by 400 km by 2100, and that total syrup production will decline in New York and over most of sugar maple's current range (except Canada and northern Maine).^214^ The lower sugar content will require more boiling and more total sap to produce the same amount of maple syrup, thus more time and energy spent to get the same product.^217^

Weather extremes, including excess rainfall and drought, affect tree health. Wind and ice storms not only damage trees, but also damage sap collection systems.^211^ Stressed trees are more susceptible to pests and diseases, which further reduce their vigor.^209^ One study found that sugar maple health declined in Vermont over a 25‐year period (1988–2012) and is expected to further decline (based on an index of forest stress) under both low‐ and high‐emission scenarios until the end of the century.^218^ Similar results can be expected for sugar maples in New York.

The risk of wildfires has also increased in New York, particularly during the summer months, as the climate has become hotter and dry periods have lengthened.^219^ On a landscape level, wildfires could change ecological habitats and the forest structure, including the growth of sugar maples; this makes fire management critically important, particularly in certain areas of New York.^220^ On a farm level, wildfires can be particularly devastating for individual producers.^221^

##### 3.3.5.2 Adaptation strategies

###### 3.3.5.2.1 Agroforestry

By its very nature, agroforestry can be a climate adaptation strategy. It helps reduce threats and enhance resilience on farms in numerous ways, such as by increasing habitat diversity for pollinators and beneficial insects, altering the microclimate to reduce heat stress on small livestock herds and flocks, improving the quantity and quality of forage production through silvopasture systems,^222^ and reducing the impacts of extreme weather (temperature, wind, precipitation, and drought) on crop production.^204^, ^223^ Within the field of agroforestry, climate adaptation strategies that have been proposed include changing the species or variety grown, making technological advancements when possible, altering postharvest practices, and shifting production to more favorable locations. Developing forest management burn plans and using controlled burns are also important strategies to enhance natural habitats and reduce the risk of uncontrolled fires.^220^

###### 3.3.5.2.2 Maple production

According to a 2015 climate change perception survey of maple producers in the Adirondacks of New York and six counties in Vermont,^209^ maple producers are adapting to climate impacts and uncertainties in a number of ways, including:

- Seeking input from experts (e.g., extension agents and foresters) for information about adaptation.
- Relying on family members to help complete tasks, due to shortened production windows.
- Planning ahead to be able to make decisions quickly if extreme weather impacts require it.
- Installing new technologies to help adapt to changing climatic conditions.
- Diversifying their customer base.
- Seeking alternative sources of income if their resource base is damaged from climate impacts (e.g., harvesting timber).

Results from the 2015 survey also indicate that a maple producer's ability to adapt is directly related to the resilience of the resource base, adaptability in technology, knowledge of northeastern forests, and knowledge of climate change impacts on production.^209^

Important adaptation strategies for maple producers are incorporating tap hole sanitation practices, vacuum systems, and energy‐efficient practices. With tap holes being left open for longer periods each season due to increases in temperature variability, it is important that producers use tap hole sanitation practices such as replacing drop lines, sanitizing tubing, or adding check valves to prevent sap from flowing back into the tap hole and leading to potential contamination.^224^ Improved spout sanitation also increases sap collection.^225^ A vacuum system allows the sap to run at colder temperatures and during extended thaws, increasing sap yields.^216^ Practices aimed at reducing energy use include reverse osmosis and more efficient evaporators because they provide more efficient water removal.^217^ These practices have allowed for continued growth in maple syrup production and increased this industry's resilience.

#### 3.3.6 Aquaculture

Aquaculture is the breeding, rearing, and harvesting of fish, shellfish, aquatic plants, and other organisms in water environments, both marine and fresh. It is essentially farming in water. Aquaculture is a growing agriculture sector in New York State, particularly as market demand for high‐quality, locally grown products continues to expand. Revenue from the state's aquaculture industry totaled roughly $8.8 million in 2018.^226^ The industry is diverse across the state, encompassing operations that produce finfish (e.g., trout, bass, salmon), shellfish (e.g., hard clams, bay scallops, and oysters), and seaweed (e.g., sugar kelp and red macroalgae). Aquaculture production in coastal water bodies is more vulnerable to the impacts of climate change than inland production, but efforts are ongoing to increase this industry's resilience to climate change.

##### 3.3.6.1 Observed and projected impacts

A variety of observed and projected climate impacts affect the growth and productivity of marine organisms.

- **Acidification**. Elevated carbon dioxide in the world's oceans is leading to acidification, which is particularly detrimental to calcifying organisms. Acidification is amplified at a local level by upwelling, riverine discharge, and eutrophication and is expected to have a significant long‐term economic impact on the aquaculture industry along the East Coast.^60^, ^227^
- **Changing water temperatures**. Long Island is at the southern end of the range for sugar kelp and some shellfish (e.g., bay scallops and near‐shore lobsters). For sugar kelp, short day lengths and cooler water temperatures trigger reproduction, and temperatures below 60°F are required for reproductive tissue to mature. Higher water temperatures in the summer and later into the fall are affecting the reproductive behaviors of seaweeds and shellfish and leading to a decrease in populations in New York waters.^228^, ^229^ For example, sugar kelp spawning has traditionally occurred between the middle and end of October but is being pushed later into the year because of warmer waters, thus shortening the growing season.
- **Parasites and diseases**. Warmer temperatures could allow new parasites and diseases to affect the growth and survival of shellfish and seaweeds. For example, the die‐off of 90−100% of Peconic Bay scallops in 2019 was linked to the coccidian parasite, a parasite never before found in New York waters.^230^ Physiological stress from higher water temperatures, low dissolved oxygen levels, elevated carbon dioxide, and other environmental factors makes scallops and other shellfish more susceptible to infections.^231^, ^232^, ^233^ Higher water temperatures also require quicker postharvest processing to minimize product degradation and the potential introduction of pathogens that can affect human health, such as *Vibrio* bacteria.^234^, ^235^
- **Heavy rainfall**. Heavy rainfall events that produce a large volume of precipitation in a short period can cause changes in salinity. Some organisms, such as clams, struggle with salinity changes; juveniles are less able to tolerate these changes compared with adults.^236^, ^237^ Closing shellfishing grounds following heavy storms due to potential fecal coliform contamination and HABs means both shellfish and kelp cannot be harvested during these periods, leading to potential crop and revenue losses.
- **Extreme wind**. Storm events with high winds can lead to loss of gear due to lines breaking and equipment becoming unsecured. This contributes to plastics and other garbage entering the environment, which is a hazard for marine life. Wind also increases wave action, and while some tumbling is good for shellfish because it hardens their shells, too much is not good, especially for juveniles (Schott S, Cornell Cooperative Extension of Suffolk County—Marine Program [2022, March, Personal communication]). This can mean crop losses for marine farmers.

These current and projected climate impacts to the aquaculture industry represent a critical factor affecting the viability of this growing sector in New York. Given current projections, the sustainability of the industry may be at stake, unless producers can adapt to the projected changes.^238^

##### 3.3.6.2 Adaptation strategies

A variety of adaptation strategies exist to address climate impacts on aquaculture in New York. Examples include:

- **Selective breeding**. Several ongoing research efforts are focused on selective breeding of various shellfish to identify genetic strains that are more resilient to temperature increases, disease, and salinity variation.^239^, ^240^ This work aims to provide a source of broodstock for various species that can be used to increase the production of seed that can survive harsher environmental and biological conditions, supporting the long‐term sustainability of the aquaculture industry.
- **Sediment amendments**. Other research in New York State has shown that amending muddy sediments with crushed carbonate shells leads to increased porewater pH, thereby making sediments more hospitable for hard clams, particularly juveniles, and likely for other calcifying organisms.^241^ This is a possible adaptation strategy for aquaculture seed clam plantings in bottom sediments, as it can alleviate some of the impacts of acidification.^241^, ^242^
- **Integrated aquaculture**. Another strategy that could help buffer the impacts of ocean acidification on calcifying organisms is growing bivalves near farmed sugar kelp. A recent study on a commercial shellfish farm found that growing oysters near kelp resulted in increased pH and decreased carbon dioxide levels in the surrounding water, leading to faster oyster shell and tissue growth rates.^243^ This result suggests that a more integrated approach to aquaculture production could increase marine farmers’ ability to adapt and build resilience.
- **Diversification**. Diversifying products and business models and improving access to markets are adaptation strategies for marine farmers, just as they are with land‐based farmers. Research is ongoing to assess the viability of various seaweeds, including sugar kelp and red macroalgae, to diversify the aquaculture industry. This diversification could mean integrating seaweed and shellfish production, as discussed above. For example, different seaweeds could be cogrown with oysters at different times of year (with sugar kelp as a winter crop and red macroalgae as a summer crop) (Shuford R, New York Sea Grant [2022, April, Personal communication]). Seaweeds also have added nutrient mitigation (bioextraction) and carbon sequestration benefits.^64^ Processing locally grown seaweeds such as sugar kelp into a fertilizer product for use by land‐based farmers could create a value‐added product and potentially new market opportunities for the industry. Seaweed fertilizers have long been used by farmers, as they are valued for their ability to provide needed micronutrients to crops and act as a biostimulant. Recent work has shed light on the possibility of expanding the use of locally grown sugar kelp as fertilizer on Long Island farms.^244^ However, challenges exist, as the infrastructure needed to accommodate drying, processing, value‐added production, and storage is limited (Shuford R, New York Sea Grant [2022, April, Personal communication]). Seaweeds could also be incorporated into feed for livestock (research is ongoing regarding the reduction of methane from ruminants when macroalgae are included in their diet).^245^
  On Long Island, oysters are the predominant species being raised commercially. The inclusion of other species would increase the sustainability of the aquaculture industry. The blue mussel and the hard clam are traditional commercial species that could be raised, though both have unique challenges and limitations. Blue mussels would likely be raised seasonally, as the species prefers cooler waters. Hard clams are slower growing than oysters and typically require sediment to be buried in during the winter. The ribbed mussel is not considered a commercial species, but it can improve water quality in ways other species cannot (i.e., reduction of fecal coliform bacteria), and it could be used as a bioextraction species. Research is ongoing in New York to determine if mussels can be incorporated into fertilizer. Increasing support (e.g., financial and logistical) for commercial shellfish hatcheries could give them a chance to expand and attempt to incorporate other species. Each year, many shellfish farmers seeking oyster seed from New York hatcheries are placed on waiting lists, while others purchase seed from northern state hatcheries (Udelson B, New York Sea Grant [2022, July, Personal communication]).
- **Shellfish restoration**. The Long Island Shellfish Restoration Project, now completed, was established to spawn and grow native shellfish to restore coastal populations and filter excess nutrients from the water, which could help reduce HABs. The Billion Oyster Project is restoring oyster reefs in New York Harbor to provide species habitat and protect the New York City shoreline from storm damage (flooding and erosion). These shellfish restoration projects are intended to improve water quality, bolster the local economy, and increase community resilience. Indigenous communities have also used shellfish restoration as a climate adaptation (e.g., refer to the Shinnecock Nation Marine and Land Farming Adaptations case study); this approach offers an integrated response to multiple climate change impacts (e.g., rising tides, food security, and ocean acidification) and draws upon traditional knowledge.^31^
- **Controlled‐environment aquaculture**. Land‐based production is another adaptation strategy that could be considered by some in the aquaculture industry. In land‐based production, the growth of fish and shellfish (during the hatchery stage) occurs in tanks under conditions that can be more readily managed (e.g., to achieve optimal temperature and salinity levels). These production systems are not affected by climatic factors such as rainfall variation, salinity fluctuation, ocean acidification, and sea level rise, all of which can have detrimental impacts on aquaculture production in the traditional coastal environment.^246^ Further, land‐based production can enhance sustainability and reduce environmental impacts in the industry. For example, carbon dioxide generated through the production process can be captured and diverted to greenhouses for crop production; farms in New York are implementing this technique.^247^ Fewer than five farms in New York are raising fish commercially in recirculating aquaculture systems. These operations can be built and operated in industrial parks using fresh water or on unused farmlands. Farms can also be designed to use natural streams or freshwater springs. Effluent from these fish farms can be used to grow plants (e.g., produce for consumption, ornamental plants for landscaping), as the water is filled with nutrients. Even effluent that is discarded could be repurposed as a fertilizer, providing an additional product from the farm. Prospective farmers have said they found the permitting process for establishing and building new fish farms cumbersome and confusing, leading some to relocate to other states (Udelson B, New York Sea Grant [2022, July, Personal communication]).
- **Decision‐support tools**. Predictive models and tools such as the new *Vibrio* Harvest Calculator^248^ can assist producers in planning safe harvest and cooling strategies. The user inputs harvest location, time, and date, and the calculator predicts *Vibrio* population doubling time. Such tools can help producers secure a harvest that has minimal potential for negative human health impacts due to warming temperatures.

Continued operational advances, both on land and in the water, will be integral to ensuring that the aquaculture industry is able to adapt and increase resilience to changing climatic conditions.

### 3.4 Cross‐commodity impacts and adaptation strategies

Many of the issues related to climate change do not have isolated impacts, but rather cut across multiple agricultural commodities and systems. Examples include uncertainty, management challenges, risk, and many types of pests (weeds, insects and other invertebrates, and pathogens), as well as impacts to beneficial species such as pollinators.

#### 3.4.1 Uncertainty, management challenges, risk

As discussed throughout this chapter, climate change increases uncertainty, management challenges, and the risks associated with farming. To address uncertainty, farmers may use precision farming technologies,^149^ apps, and climate decision‐support tools to make better‐informed decisions.^32^ To address management challenges, when replacing equipment, farmers may purchase newer equipment, if financially possible, to increase fuel efficiency and decrease labor and time constraints, and they can also develop adaptation plans.^71^ To address risk, farmers may diversify their operation (production practices, products, and/or cropping systems), consider controlled‐environment agriculture (if applicable), renovate or build energy‐efficient buildings to withstand extreme weather, consider purchasing crop insurance (when possible) to reduce economic risks, adjust the timing or location of farming activities, or improve soil health, among many other opportunities.^71^

#### 3.4.2 Pests

Climate disruptions to agricultural production have increased in the past 40 years. As a result, life cycles of different pests have changed substantially (e.g., growth rates, altered mortality) in recent years, and their negative effects are expected to multiply.^249^ Climate change is now a major factor driving the spread and establishment of agricultural pests (insects, mites, ticks, nematodes, plant diseases, weeds, wildlife) across New York State and beyond. At the same time, there is evidence that the efficacy of pesticides is reduced under changing environmental conditions such as elevated temperatures and enriched carbon dioxide levels. Range expansion, life history, population dynamics, and behavior are four areas where emerging pests demonstrate responses to climate change that create significant biological and economic issues^250^ (refer to the Ecosystems chapter for a discussion beyond agriculture). Climate change, for instance, is the primary driver of deer range expansion, and studies have found that higher deer densities are associated with greater damage in agricultural systems.^251^, ^252^, ^253^ It is critical that farmers build resilience by having a sound climate‐smart pest management program.^107^

Pesticides play an important role in pest management when no other tools are effective and when used in combination with other tools. Decisions about their use are based on economic thresholds and a determination of whether conditions are appropriate—both to target the pest and to reduce the impact on nontarget species and people. As climate conditions change, farmers will experience higher pest pressure, likely resulting in an increased use of pesticides,^74^, ^254^ which could lead to pesticide resistance.^255^ Warming can shift phenologies, increase the magnitude of nontarget effects, and disrupt important species interactions (i.e., biological control, competition) instrumental for the sustainable management of pests in agricultural systems. Increased pesticide use will increase the manufacture, transport, and application of pesticides, all of which contribute to greenhouse gas emissions.^256^ Potential health risks to agricultural workers due to more frequent pesticide applications must also be considered.

##### 3.4.2.1 Weeds

Increasing atmospheric carbon dioxide, higher temperatures, and variable precipitation due to climate change impact all aspects of weed biology, including plant growth, reproduction, and distribution.^257^, ^258^ The impacts may be positive or negative and will vary by weed species, especially between those that use the C3 photosynthetic pathway and those that use C4.^75^, ^76^, ^77^, ^259^ C3 weeds respond to rising atmospheric carbon dioxide concentrations by accumulating biomass growth; C4 weeds also respond by accumulating biomass, but less markedly so. As weeds become more competitive with other crops in the field, there will be greater seed production and thus more weeds to deal with over time. Increased soil temperatures and milder winters mean a longer period of active growth for weeds.^260^

Concurrent changes in temperature, especially minimum winter temperatures, can result in significant shifts in weed survival and distribution.^76^, ^106^, ^261^ Although each plant group contains both weeds and cash crops, researchers have found that the weeds typically show a more positive response to rising carbon dioxide than the cash crops they compete with, resulting in increased weed pressure and higher weed management inputs.^75^, ^76^ Weather factors affected by climate change, such as humidity, temperature, and precipitation, also affect the efficacy of herbicides, one of the most important tools for weed control.^75^, ^76^, ^77^ This could result in increased herbicide use and weeds developing resistance.

In fact, several weed species are becoming more problematic for farmers across New York State. For example:

- **Palmer amaranth** exhibits resistance to different herbicides and is a major concern for growers as it has become a dominant species and is adapting well to a wide range of environmental fluctuations.
- **Horseweed (or marestail)** showed no herbicide resistance in New York State as of 2021, but resistance screening efforts are ongoing. These population screenings appear to show resistance to common pesticides including glyphosate and paraquat. As horseweed seed spreads by wind currents, infestations and resistance are likely to occur.^260^
- **Waterhemp** is showing resistance to different herbicides. Its seeds spread easily on farm equipment and by wind.

While research is ongoing to monitor weed distributions across the state and specific species’ resistance to different herbicides, farmers share significant concern about increased competition between crops and weeds. Trials continue to examine the effectiveness of newer technologies, such as electric weed zappers. Farmers will need to implement appropriate weed control methods on their farms and remain aware of how climate change is altering weed populations.

##### 3.4.2.2 Invertebrate pests

Changes in climate will also affect insects and other invertebrates such as ticks and mites. Invertebrates are cold‐blooded organisms, regulating their body temperatures according to ambient temperature conditions. Temperature is the single most important abiotic factor influencing insect reproduction, survival, development, behavior, and distribution.^262^ Insect life cycles are often calculated based on cumulative degree days from a base temperature and biofix point.^263^ Recent research has shown that the effect of temperature largely overwhelms the effect of other environmental factors.^264^, ^265^ A 2°C temperature increase could translate to up to five generations per year for some insect pests.^266^ Furthermore, as the U.S. climate shifts, previously wet or cold regions will begin to resemble warmer and drier neighboring regions, and pest ranges will expand to new areas.

In addition to expanding the range or accelerating the life cycle of established pests, warming temperatures could facilitate the introduction and successful establishment of new invasive species in New York. A county‐level invasive species tracking tool is available online.^267^ The following are examples of new invertebrate pests that threaten agriculture in New York:

- The **spotted lanternfly** is an invasive pest from Asia that quickly spread over multiple states in the Northeast. One study estimated a worst‐case potential economic impact of over $500 million per year in Pennsylvania (New York impacts were not estimated).^268^ The spotted lanternfly was first detected in New York State in August 2020. It poses the greatest impact to grape production, but other crops are at risk too. Ongoing studies indicate that warming temperatures could increase the areas in New York that are suitable for this insect to transition to different life stages.^124^
- The **European cherry fruit fly** is an invasive from Europe that was first detected in New York State in 2017. If not controlled, it threatens to have a huge potential impact on both sweet and tart cherry production, with crop losses of 100%, valued at $873 million.^269^
- The **Asian longhorned tick** is another invasive from Asia that was first detected in New York State in 2017. The economic impact of this pest for the state is unknown at this point. It is not just a pest to crops, but also one that can pose serious risks to human health.
- The **soybean cyst nematode** is an invasive from Japan that was first detected in New York State in 2016; the statewide economic impact of this pest is unknown. However, studies conducted outside the state indicate that this pest could cause economic losses in the United States of nearly $1.5 billion annually.^270^

Ultimately, if some farmers choose to stop growing certain crops because the conditions to grow them profitably no longer exist, some pest populations could decrease in size. Otherwise, increased temperatures are expected to increase pest populations and the corresponding risks.

Beyond the risk to crops, certain invertebrate pests can also harm humans and livestock. For example, increased rodent and deer activity in agricultural areas is contributing to the dispersion of ticks and tick‐borne diseases, which are becoming a problem in areas where ticks have been able to establish themselves and survive as a result of warmer winters.^271^, ^272^ Ticks pose risks of disease for humans (discussed further in the Human Health and Safety chapter) and livestock. For example, ticks can transmit Lyme disease to horses and transmit anaplasmosis to horses and cattle. More than just a nuisance, tick‐borne diseases in cattle can lead to fever, weight loss, anemia, dehydration, weakness, and labored breathing.^273^

##### 3.4.2.3 Crop pathogens

Weather patterns significantly influence plant pathogen distribution and epidemiology.^274^ Based on projections for warmer and wetter seasonal patterns and increases in the number of significant storms (which can move pathogens long distances), an increase in disease incidence and severity in agricultural crops can be expected.^76^, ^275^, ^276^, ^277^ Major precipitation events that cause flooding in fields produce optimal conditions for many soilborne diseases to spread and decimate crops. Many important foliar diseases thrive in high humidity, and high rates of reproduction and spread can result in severe epidemics. These outcomes will translate into an increased reliance on fungicides and other tools for disease management, such as a need to breed for locally adapted varieties with improved disease resistance. As farmers experience higher input costs to produce the crops, the costs are passed on to consumers in the form of higher food prices.^78^ One example of a crop pathogen is the plum pox virus, a major plant disease that was first detected in New York State in 2006. This virus poses a serious threat to the stone fruit industry by reducing the yield and marketability of the fruit. Another pathogen, *Fusarium virguliforme*, leads to soybean sudden death syndrome and can greatly reduce soybean yields. Since it was first confirmed in New York in 2012, the disease has expanded rapidly throughout the state and has become a major concern because it is exacerbated by wet springs and dry growing seasons—exactly the pattern climate change is creating.^278^

#### 3.4.3 Pollinators

Crop pollination by insects is an important ecosystem service. Nearly 35% of global crop production benefits from insect‐mediated pollination.^279^ Climate change is “potentially the most severe threat” to pollinators, especially as there is historical evidence that relatively small changes in environmental factors can result in significant responses by different species of pollinators, including birds, bats, and insects.^280^ Increases in average temperatures and the frequency of extreme weather events are leading to detrimental impacts on insect diversity,^281^ reproduction, survival, and crop production.^282^, ^283^ Several mechanisms are involved, including a reduction in the amount of and access to habitat and food resources that are essential for pollinator survival.^284^

Pollinators, including insects, birds, and bats, contribute substantially to the production of crops in New York, including apples, grapes, and pumpkins. It is estimated that pollinators provide approximately $389 million worth of pollination services to the state each year.^285^ A 3‐year study conducted by the New York State Department of Environmental Conservation (NYSDEC) found that between 38% and 60% of studied pollinators are at risk due to several factors, including climate change.^286^ The potential impact on crop production in the state remains undetermined, but recent studies conducted in similar geographical areas indicate that a warming climate affects the synchrony of plants and pollinators,^287^ which has a detrimental impact on crop yield and quality.

Adaptation strategies for pollinators include reducing or eliminating pesticide use, installing bat boxes, establishing pollinator gardens, and planting a diversity of plants, including native plants.^288^ Pollinator gardens should have native plants, a diversity of plant sizes, shapes, and colors, and continuous blooms throughout spring, summer, and fall.

### 3.5 Economic and labor impacts and adaptation strategies

The impacts of climate change on agricultural production extend beyond the biophysical issues discussed in previous sections. Climate change also has many socioeconomic impacts, affecting almost every part of the agricultural supply chain, from operational costs, worker health and safety, food storage, and transportation to commodity and consumer prices.^20^ Because climate change is often just one of many complex factors affecting the agricultural supply chain, quantifying the effects caused by climate change alone is difficult. Often, these impacts depend in part on the geographic scope of the markets involved. While some agricultural commodities are traded globally, with pricing influenced by international trade, others (e.g., fresh milk) are mostly traded locally, which makes those markets more vulnerable to local climatic shocks. One of the most critical climate impacts that New York farmers talk about is the overall level of uncertainty.

1 The climate is not stable like it was in the past. This year is not like last year, and next won't be the same.

—Field crops farmer, Central New York (2022)

The subsections below discuss observed and projected socioeconomic climate impacts in more detail. They also discuss private and public adaptive responses that can enhance resilience to increasing climatic risk.

#### 3.5.1 Observed and projected impacts

Key socioeconomic impacts of climate change that are associated with agriculture include crop yield, farming profits, TFP, crop insurance payments for losses caused by extreme weather, and heat‐related health impacts to agricultural workers.^16^

- **Crop yield**. Agricultural trade is critical in determining price changes resulting from swings in crop yields.^289^ At the global scale, evidence suggests that while maize and wheat yields have continued to grow over time due to nonclimate factors (e.g., farming practices, industry trends, technologies), historical climate trends have slowed this growth somewhat; the effect on rice and soybeans, however, remains regionally mixed.^290^ Global studies seeking to directly attribute these yield trends to anthropogenic sources also find largely negative effects.^291^, ^292^ Unfortunately, there is a limited number of country‐level studies, including in the United States. There is also a deficit of studies exploring historical impacts on specialty crops and livestock.
- **Total factor productivity**. Some studies have sought to explore the aggregate impacts of historical climate change on the entire agriculture sector. To do this, researchers have relied on sector‐wide measures of TFP, which essentially measures the ratio of all agricultural outputs (crops and livestock) to all agricultural inputs (labor, land, capital, etc.). Climate change has slowed the growth of global agricultural TFP.^293^ The magnitude of this slowdown is substantial, equivalent to losing about 9 years of productivity growth^293^ over the past 60 years. The historical effects of climate change on U.S. agricultural TFP are not yet documented, but evidence suggests an increasing sensitivity of U.S. agricultural TFP to higher temperatures.^294^, ^295^
- **Agricultural product quality**. While impacts on crop yields have received considerable attention, less is known about impacts on agricultural product quality. Quality greatly affects the price farmers receive, so it remains a critical component of production. Moreover, quality is a complex measure because it depends on the intended end use. The relevant quality metrics can vary by product, final intended use, and region. In the case of milk, higher temperatures are linked with lower milk yields,^296^ but such conditions have been shown to also affect milk quality.^297^ Specifically, heat stress is associated with lower protein content in milk.^298^ In the case of corn, quality metrics largely depend on the intended use.^299^ There are few studies exploring the effect of heat or drought on corn grain quality. Scientists have some understanding that extreme weather can affect corn silage quality, although effects are mixed^300^; while drought stress might increase fiber digestibility, heat stress might decrease it.
- **Agricultural prices and food security**. Climate change can influence agricultural prices. Because market prices are determined by an equilibrium between supply (production and inventory) and demand (consumption for a variety of uses), a reduction in production can lead to higher prices. While an isolated shock can be buffered through changes in inventory—at least for commodities that can be stored—consistent reduction in production can lead to higher price levels. An important factor in regulating prices is international trade. Trade allows shocks in one region to be compensated by surpluses in other regions, which reduces price spikes. However, when the production of certain commodities is hyperspecialized in certain regions of the world, a drought in these regions can have a major impact on price levels at a global scale. For example, consider the price spike in wheat that began in 2022 because of the war in Ukraine, a major wheat producer.^301^, ^302^ How climate change will affect future prices depends greatly on how the demand for and supply of agricultural products evolves, as well as on the integration of agricultural markets at a global scale. Expanded investments in research and development will lead to higher productivity, meaning more output per unit of input. This will tend to keep prices lower.^303^ As evidence suggests that climate change is slowing agricultural productivity growth,^293^ the tendency is for climate change to put upward pressure on food prices. However, the exact impact of climate change on prices remains largely uncertain for the aforementioned reasons. Trade, inventories, and the availability of product substitutes are likely to play a critical role in managing and attenuating price risks for certain highly traded commodities. Price levels for food have direct impacts on food security.^304^
- **Crop insurance payments**. Research has shown that climate change is having a substantial impact on rising crop insurance losses. For example, one study estimated that warming trends contributed $27 billion, or 19%, of the crop insurance losses recorded between 1991 and 2017 in the United States.^305^ These costs are expected to keep rising in the coming decades, driven by rising temperatures.^306^ There is also some evidence that the expansion of subsidized crop insurance products is discouraging farmers from adapting more climate‐resilient cultivars and practices.^307^ Higher risks to production will result in tighter profit margins for farmers and an increased reliance on crop insurance, which could translate into higher costs downstream for consumers.^308^
- **Worker health and productivity**. There is evidence that higher temperatures negatively affect workers’ health and productivity, although much of this evidence focuses on workers in manufacturing settings.^309^, ^310^ Some evidence also suggests that higher temperatures have large negative effects on agricultural workers.^311^ (Refer to the Human Health and Safety chapter for a more detailed discussion on the effects of heat exposure on outdoor workers.) In the United States, the number of unsafe days due to extreme heat will double by the middle of the 21st century and could even triple by the end of the century without serious efforts to reduce climate change.^312^

#### 3.5.2 Adaptation strategies

Adapting to climate change requires strategies that help farmers cope with extreme events. Climate change affects crop and livestock production in multiple ways, resulting in a changing risk profile for farmers. Some choices that farmers face may be reasonable under a certain climate scenario but become too risky under a different scenario. Adaptation occurs at multiple levels, so while farmers can carry out adaptive actions at the farm level, the actions of other players in the agri‐food supply chain also affect the resilience of the sector.

- **Self‐insurance approaches**. Farmers can invest in equipment or change their production mix in ways that reduce their sensitivity to climate extremes, but such “self‐insurance” strategies are not a panacea. For example, investing in an irrigation system could help reduce yield losses in a drought year, but the system would provide relatively little value in other years. Similarly, a highly diversified production mix could raise operational complexity and costs, but many cobenefits exist, so the need to diversify will differ depending on the situation. These strategies are essentially trade‐offs, where the farmer accepts lower average returns in exchange for reducing risk. While these strategies may be attractive in many contexts, there may be superior ways of coping with extremes.
- **Commercial insurance approaches**. Some farmers may be able to buy commercial insurance products that provide compensation when extreme weather occurs and negatively affects their financial returns. With these products, farmers pay insurers to take on their risk in exchange for a premium. Such insurance products allow farmers to adopt production systems that are more profitable under the most frequently occurring conditions without worrying excessively about their finances during a bad year. Agricultural insurance policies have various limitations at present. Most of these policies are only available for a handful of major field crops (e.g., corn and soybeans); New York State farmers grow a wide range of specialty crops that are not yet covered by many of these insurance policies. Moreover, current insurance products are highly subsidized to encourage widespread adoption. This suggests that farmers do not yet appreciate the market value of these insurance products. More progress is required regarding the coverage and the design of these products to ensure more widespread adoption across the state.

### 3.6 Cascading and cross‐sector impacts

Climate change can cause cascading impacts across many socioeconomic and environmental systems that are interrelated.^313^ This means that impacts in other sectors can ultimately affect agriculture, just as direct impacts to agriculture can lead to challenges in other sectors, such as human health and water resources. This section explores some of the cascading and cross‐sector impacts associated with the agriculture sector.

#### 3.6.1 Buildings and infrastructure

Buildings are a critical part of a farm's infrastructure. In the case of some urban farms, buildings provide the foundation for their production. Extreme weather events are leading to more frequent damage to farm structures. For example, extreme snowfall in Western New York in 2014 led to greenhouse collapses,^314^ and heavy rain in 2021 led to the flooding of a greenhouse and temporary housing.^315^ The risk of such events requires farmers to find ways to adapt. When replacing equipment or infrastructure, farmers should invest in more energy‐efficient or resilient equipment or infrastructure, if financially possible, to reduce risks and better prepare for the future. Some farmers are constructing new structures to withstand higher windspeeds and greater snow loads. Drainage and irrigation systems for rooftop farms can include stormwater management in the design to manage high water volumes and mitigate flood risk.^316^ Farmers are also renovating greenhouses and other structures to enhance energy efficiency, with better lighting, temperature sensing, and renewable energy sources (e.g., solar and geothermal).

Dairy farmers trying to reduce heat stress to cows are looking to upgrade facilities with better ventilation and cooling mechanisms, including fans and sprinklers.^317^ Additionally, the use of shipping containers to grow crops (mostly leafy greens) hydroponically in urban/suburban areas is also increasing. This approach offers a controlled environment for growing, which reduces insect and disease issues, allows for year‐round harvests, and vertical growing opportunities, so more food can be grown in a smaller area.^318^ For maple sugaring, sugar houses and pump houses that collect the sap are located at the lowest point on the landscape. The risk of flooding to buildings and damage to both equipment and structures is potentially high if a heavy rain event occurs.^216^

Refer to the Buildings chapter for additional information.

#### 3.6.2 Ecosystems

A farm is itself an ecosystem—a complex network of diverse interacting microbial, animal, and plant species and their physical environment. In fact, the field of agroecology (the application of ecological principles to agricultural systems and practices) is becoming increasingly recognized as an important means of adapting to climate change. Agriculture can have many impacts on ecosystem services, depending on farm management practices, and these impacts can be exacerbated in a changing climate. Negative impacts can include contributions to water quality impairments, habitat fragmentation, and sedimentation of waterways.^319^ Positive impacts related to sustainably managed farmland include preserving and restoring critical habitats, protecting watersheds, and improving soil health and water quality.^320^ Moreover, many farmers own and manage land that does, or could, provide a variety of additional ecosystem services (e.g., woodlots, and pollinator and wildlife habitat). However, climate change is straining some ecosystem services that support agriculture, such as healthy freshwater resources.^321^ Maple production and pest management are examples of important connections between agriculture and ecosystems. Afforestation of the 1.7 million acres of idle and underused land in New York that was previously in agriculture^322^ could also help to mitigate greenhouse gases through carbon sequestration and could increase the adaptive capacity of key sectors of the state's economy. Refer to the Ecosystems chapter for additional information.

#### 3.6.3 Energy

As noted earlier in this chapter, adapting to climate change could require New York's growers to use more energy for supplemental cooling, irrigation, controlled‐environment agriculture, and other measures. Agriculture is also inextricably connected to efforts to mitigate climate change by evolving the state's energy sources—a connection that could influence what crops are grown in New York and create new financial opportunities or challenges for farmers. The state's Climate Leadership and Community Protection Act (Climate Act), effective as of January 2020, officially set the goal of achieving a 100% carbon‐neutral economy by 2050.^323^ An updated draft scoping plan released in December 2021 provided more specific details on the strategies needed to meet the goals listed in the Climate Act: 40% reduction in greenhouse gas emissions by 2030, 70% renewable electricity by 2030, and 100% zero‐carbon electricity by 2040. As discussed in the paragraphs below, New York's agriculture sector will probably provide some of this renewable energy in the form of biofuels, and it will also be a likely source of land for the installation of wind and solar power‐generation facilities. These installations will affect land prices and land availability for agricultural production but could also boost farmers’ financial resilience in the face of other threats. Many solar arrays are being proposed on prime farmland, and solar energy generation and farmland preservation are at direct odds, as most of the proposed or planned solar array installations on farmland do not include plans for continued agriculture production. The promotion or requirement of coproduction of solar energy and crops (agrivoltaics) would be beneficial (O'Neil K, Cornell Cooperative Extension North Country Regional Ag Team [2023, July 20, Personal communication]). Refer to the Energy chapter for additional information.

- **Biofuels**. Low‐carbon fuels, or biofuels, are needed to meet the greenhouse gas emission reduction targets set by the Climate Act. Biofuels can be gaseous (e.g., biomethane obtained from anaerobic digestion of organic waste) or liquid (e.g., bioethanol obtained from corn, or biodiesel from vegetable or waste oils). Biomethane comes from capturing the energy in livestock manure and other organic waste (e.g., food waste) that would otherwise emit methane, thus leading to substantial reductions in the life cycle carbon intensity of fuels.^324^ Bioethanol use and production may lower harmful greenhouse gas emissions by 46% compared with pure gasoline.^325^ Western New York Energy (WNYE) is the only commercial liquid biofuels facility currently operating in New York State (as of 2022), producing over 60 million gallons of renewable, high‐octane, low‐carbon bioethanol per year. WNYE's current levels of bioenergy production require roughly 20 million bushels of field corn annually, and most of this corn comes from New York State producers.^326^ However, as the bioenergy sector continues to grow, the amount of corn required will increase. This increase in intensively managed corn acres may have negative environmental and ecological impacts, both on and off farm.^327^, ^328^, ^329^, ^330^ While New York corn farmers produce 72% more bushels of corn per acre than they did 20 years ago (due to improvements in genetics, fertility, soil management, equipment, etc.), there are strict requirements on the quality of corn that goes toward bioenergy production. Climate change is affecting the quality of crops that are needed for biofuels. As described in Section 3.3.1.1, as the climate becomes warmer and more humid, the state's corn production faces increased risks from mycotoxins, the general term for toxic compounds produced by many types of molds or fungi. Deoxynivalenol (DON), commonly referred to as vomitoxin, is just one type of mycotoxin that can be present in corn. WNYE has observed higher DON levels in corn screenings and fines tested from corn grown statewide. In 2021, WNYE had to bring in Midwest corn trains to provide corn with low vomitoxin levels to blend with New York State corn (Winters T, Western New York Energy [2022, February, Personal communication]). The increased frequency of mycotoxin outbreaks has become an impactful economic hardship for New York's grain producers, warranting further research.^331^
- **Wind and solar energy**. Wind is New York's second‐largest source of renewable electricity, after hydroelectric. Power generation from wind accounted for almost 3.3% of all utility‐scale net generation in the state in 2021. In 2022, the state had a total of about 2200 megawatts of wind capacity at the state's 29 utility‐scale wind farms.^332^ Agricultural lands tend to have characteristics needed for the development of wind energy projects (e.g., open space, abundant wind), and New York had 490 farms with wind turbines in 2017, up from 317 farms in 2012.^14^ Wind energy projects have provided much‐needed additional revenue to farmers through leasing and royalty agreements and have helped diversify local economies, particularly in rural areas.^333^ While wind turbines have a small footprint once completed, there is the potential for permanent loss of productive farmland resulting from the installation of access roads, turbine towers, and connection facilities. Loss of crops, displacement of grazing animals, and permanent damage to soil resources are also possible.^333^ However, the New York State Department of Agriculture and Markets (NYSDAM) has established guidelines to minimize the impact of wind energy projects on agricultural lands.^334^

Solar energy and agrivoltaics offer farmers a similar opportunity to supplement farm income, with potential adaptation benefits from growing crops between rows of photovoltaic panels and grazing livestock beneath the panels, as discussed in Section 3.3.

#### 3.6.4 Human health and safety

As described in the Human Health and Safety chapter, the impacts of climate change on human health vary across populations and depend on many factors. Certain occupations, such as farmwork, have a greater risk of exposure to climate impacts.^335^ Climate‐related health risks faced by farmworkers include illnesses caused by pests, heat stress, and mental health issues due to chronic stress associated with fear of crop failure and loss of one's livelihood, as detailed below:

- **Parasites and vector‐borne diseases**. An important cross‐sector impact of climate change is the expanded range of parasites, including ticks and mosquitos, and associated vector‐borne diseases transmitted to humans or other animals.^336^ An example is the prevalence and expanded life cycles of ticks in areas where they previously were unable to survive during the winter and had short generation times.^337^, ^338^ Most ticks are active when temperatures are above 40°F.^339^ Projected temperature increases in New York State will expand the geographic range for ticks and place more farmers and agricultural workers at higher risk of exposure, with some workers being unaware of the risk and the steps needed to limit exposure.^340^ As a result of this increased risk, the 2021 New York State Senate Bill S4089 instructed NYSDAM to implement a public awareness campaign on Lyme disease and other tick‐borne diseases focused on the agricultural community, with an emphasis on helping farmers and farmworkers take preventive measures, recognize symptoms, and determine available treatments.^341^
- **Heat‐related illnesses**. The projected increase in extreme heat puts anyone who works outside, including farmers and farmworkers, at especially high risk of dehydration and other heat‐related illnesses. Extreme heat not only reduces the capacity for physical activities such as planting, weeding, spraying, and scouting, but also negatively affects the socioeconomic well‐being and health and safety of farmworkers.^342^, ^343^, ^344^, ^345^ Heat stress disproportionately impacts undocumented individuals and others who may not have the resources to seek assistance. The *OSHA Technical Manual*, published by the Occupational Safety and Health Administration, provides technical information about workplace hazards, including heat stress, and instructions on protecting employees from heat‐related hazards and resulting injuries and illnesses.^346^ The New York Center for Agricultural Medicine and Health also offers resources and trainings related to heat and cold illnesses and injuries, as well as many other topics.
- **Mental health**. Mental health is a critical, but often overlooked, challenge among farmers nationwide. The Centers for Disease Control and Prevention reports that the suicide rate among farmers in the United States is 1.75 times higher than the rate for the general population.^347^ The 2018 Farm Bill reauthorized the USDA Farm and Ranch Stress Assistance Network to develop a network that assists farmers, ranchers, and other agricultural workers with stress management and provides a pathway for improving mental health awareness and access. NY FarmNet, an organization that directly assists New York State's farmers in times of crisis and change, reports that the number of farms experiencing high personal and family stress increased by 85% between 2018 and 2021.^348^ Many factors contribute to the stress and anxiety associated with farming. While farmers are resilient and adaptable, they are also more vulnerable to drought‐related mental health risks than other parts of the population.^349^ There is constant physical, emotional, and financial stress associated with farming, and climate change exacerbates these existing stressors.

1 I am a member of a support group for farmers that feel like they are suffering from depression. It was started by a farmer in New Hampshire for New England farmers, but 75% of the members are from New York State.

—Farmer, Rensselaer County, New York

(April 2022)

 

- **Food safety**. There is a straightforward connection between climate change and food safety: warmer and wetter conditions create an environment more conducive to the growth of bacteria and fungi. In addition, an increase in extreme weather events that cause power outages can affect food storage and handling, leading to an increase in foodborne illnesses.^350^

#### 3.6.5 Society and economy

The Society and Economy chapter examines the impacts of climate change across many dimensions of human well‐being, including demographics, economics, education, governance, culture, and the social fabric of New York's communities. Agriculture is closely tied to the social and economic well‐being of many communities across the state, and thus there are several ways in which the impacts of climate change on agriculture could contribute to broader social and economic consequences.

There is a direct link between increasing atmospheric carbon dioxide concentrations and the decreasing nutritional quality of food.^351^, ^352^, ^353^ At the same time, the cost of food is increasing. Inconsistent access to healthy nutritious foods disproportionately impacts low‐income and underserved, overburdened populations. Through the Farm‐to‐School Program, NYSDAM offers a way to connect schools and local farms, with the goals of strengthening local agriculture, improving student health, and enhancing awareness of the regional food system.^354^

Demographically, farmers are an aging population in New York. The average age of the state's farmers increased from 54 to 57 between 2007 and 2017.^115^ Farmworkers, particularly undocumented immigrant workers, play a vital role in agricultural production across the United States, and New York is no exception. Half the farmworkers in the state are undocumented.^355^ A 2017 analysis estimated that without undocumented agricultural workers, there would be 1080 fewer farms in the state, resulting in 21,672 fewer on‐farm jobs and a loss of $1.37 billion in direct agricultural production. Off‐farm and nonfarm impacts would be even larger, including 23,490 fewer off‐farm jobs and a $7.2 billion reduction in nonfarm economic activity.^356^ It is important that policies and programs are in place to support farmworkers in the state and beyond because they perform difficult jobs that others often do not want to do, and they contribute to keeping food affordable.^15^

Across the United States, revenue from agritourism—farms that contain a recreational or educational enterprise component—more than tripled between 2002 and 2017, according to data from the Census of Agriculture.^14^ In New York, agritourism is a significant part of the economy, particularly for Long Island and the Hudson Valley, which are “hot spots” for agritourism due to their proximity to New York City.^357^ Agritourism allows farms to diversify, offering an “experience” to consumers and boosting the economy of communities through jobs, retail, and other services. This strengthens and diversifies the income stream for farmers who are facing more uncertainty due to climate change.

#### 3.6.6 Transportation

Transportation is essential to the agriculture industry, linking all aspects of the supply chain from suppliers to farmers to consumers.^358^ Farmers must get their goods to market or suffer economic losses; extreme weather that causes infrastructure to fail disrupts essential transportation both directly and indirectly.^359^ In 2011, Hurricane Irene washed out a road in Schoharie County, preventing a local dairy farm from transporting milk and manure off the farm.^360^ In 2014, an extreme winter storm in Western New York dropped more than 5 feet of snow in a short time period; blocked roads and collapsed buildings prevented milk trucks from reaching dairy farms, causing farmers to dump their milk.^361^ Farmers, collectives, and municipalities need help to prepare for these types of extreme events, which will occur with greater frequency in the future.

Rural roads across the country are increasingly in poor repair, and all roads are compromised by climate extremes such as freezing and thawing, heat, and moisture. The growing need for road maintenance may outstrip government budgets.^362^ A farmer survey from the upper Midwest found that as “farms have grown larger and livestock more concentrated, agricultural machinery and vehicles have become too heavy for rural roads as originally engineered.”^359^ Similar issues are likely in New York State. Further, an efficient and reliable transportation system is critical to move goods and products quickly because, for example, warmer temperatures can increase the risk of heat stress to animals during transportation.^363^

The rise in urban agriculture and a shift to a more local food system in New York could help reduce food transportation needs. For example, New York City already has a robust transportation network and physical infrastructure that supports urban agriculture while reducing the number of miles food needs to travel.^364^

Overall, it is critical that transportation and supply chain infrastructure be upgraded to better withstand the weather extremes that are becoming more frequent and severe.^365^ Refer to the Transportation chapter for additional information.

#### 3.6.7 Water resources

Water for irrigating crops requires withdrawals from surface and groundwater supplies. The NYSDEC regulates water withdrawal for irrigation and requires permits, depending on the amount and rate of water use. The number of irrigated acres in northeastern states has increased over the last 30 years (Figure 3-2), and agricultural water use is expected to continue to increase in the region over time.^45^ Water availability is not expected to be a limiting factor for agriculture in New York, as it is in other agricultural regions.^366^ However, there may be a strain on water resources during critical points of the growing season due to predicted increases in both drought and excess water extremes and the increased frequency of late‐summer and early‐fall dry periods.^3^, ^8^

**FIGURE 3-2:**
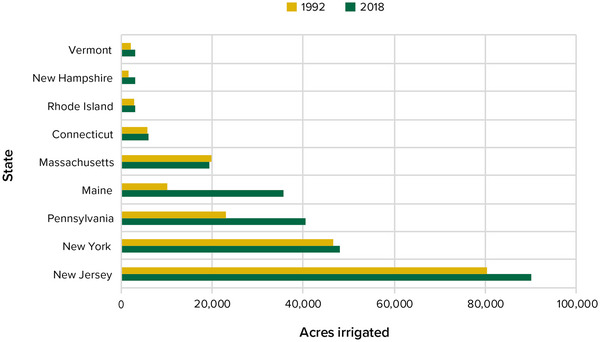
Irrigated agricultural acres in northeastern states in 1992 and 2018. Data from USDA, National Agricultural Statistics Service (1999, 2019).^13^, ^367^

Excess water leaving farms means greater potential for nutrients and pesticides to pollute surface waters through runoff and groundwater via infiltration. As described in the Ecosystems and Water Resources chapters, the combination of increased temperatures and excess nutrients entering water bodies can contribute to water quality issues, including an increase in HABs.

## 4 VULNERABLE POPULATIONS AND SYSTEMS

This section examines agricultural communities and populations at risk from climate impacts. It also looks at equity and justice concerns, broader agricultural systems at risk, and nonclimate stressors that climate change can exacerbate.

### 4.1 Agricultural communities and populations at risk

Farming is an inherently risky livelihood. Many involved in this sector face substantial economic stress; with enough bad harvests, entire livelihoods can be lost. Moreover, a large portion of the people involved in farming in New York face additional challenges that compound the inherent risks of farming. For many, simply running a small operation in a rural community creates added vulnerability because of relatively limited access to resources to cope with climate hazards. Coastal and urban farmers also face added challenges related to their geographic location. Farmworkers in general face the added vulnerability of largely working outdoors, while undocumented workers face numerous challenges that limit their ability to cope with climate hazards. Other demographic groups in New York with unique compounding stressors include farmers of color, Indigenous farmers, veteran farmers, and Amish farmers. The sections below provide an overview of concerns related to each of these vulnerable populations, recognizing that some individuals might belong to more than one of these groups.

- **Rural farmers and communities**. In New York, 48% of counties, cities, town, and villages are classified as small, rural municipalities with a population under 2500.^368^ Most agricultural production occurs in nonurban areas of the state (rural or suburban), and rural areas provide much of the food production and many of the ecosystem services that supply the larger urban areas.^369^ Because of climate change, farmers in these communities face considerable risks to agricultural production, as well as risks to their livelihoods, infrastructure, and quality of life.^3^ The 2020 poverty rates in New York were 12.8% for urban counties and 12.7% for rural counties, although there are differences in the challenges faced by people in these areas.^370^, ^371^ New research demonstrates that the state's rural communities have taken less climate action than their urban counterparts; key barriers to climate action include a lack of human, financial, and technical resource capacity.^368^ Small family farms remain one of the most vulnerable groups to the effects of climate change, with little capital to invest in on‐farm adaptation strategies.
- **Coastal farmers**. According to the blended multiscenario estimates developed for this assessment, sea level along the state's tidal coastline and in the tidal estuary of the Hudson River is projected to rise 2–3 feet by the 2080s, relative to the 1995–2014 average.^33^ The likely range for Montauk extends to 41 inches, and would be even higher in a rapid ice‐melt scenario.^33^ This would lead to loss of farmland, particularly on Long Island, and the potential loss of livelihood for many farmers in this part of the state. Saltwater intrusion is already affecting coastal agricultural wells and will further affect the productivity of coastal farms.^52^ Coastal flooding discourages farming in certain areas, as does recurrent flooding from heavy precipitation events. Many coastal areas also face development pressure, leading to a loss of farmland and livelihoods; increasing the cost of living and labor; and further marginalizing overburdened communities.^372^
  For farmers—and all residents—along the Great Lakes shorelines, flooding has long been a concern. While the latest climate models cannot project with high confidence whether lake levels will increase or decrease overall in the future, there is general agreement across models that annual and multiyear variability in Great Lakes water levels will increase.^33^ Moreover, compounding impacts on water levels caused by lake seiches and high river flow could result in a higher probability of flooding.^33^ Flooding along the Lake Ontario shoreline between 2017 and 2019 led to more than $1 billion in total damages.^373^ Increased flooding in rural areas, where most agricultural land is located, will also result in greater soil erosion and nutrient export off farms.^374^ The Flood Inundation Mapper, an online tool developed by the U.S. Geological Survey, can be useful for evaluating the potential for flooding and can help individuals to make better‐informed decisions.^375^
- **Urban farmers**. Urban areas face the challenges of limited farmland and high land costs. The most effective way to preserve urban farms is to transfer the land to a land trust or to have a public entity purchase the land.^63^ Finding people who are interested in working in agriculture in cities can also be difficult because many people move to cities for better opportunities. For example, people in some immigrant communities left their home countries in search of a better future; for them, agricultural work is not considered a desirable occupation (Schouten J, Brooklyn Grange [2022, July, Personal communication]). However, urban farms need workers, and if labor is short at certain times of the year, it can be difficult to complete all the necessary planting, harvesting, and processing within narrow weather windows. Farmers and farmworkers in urban areas of the state face the added challenges of urban heat islands and poor air quality. Refer to the Urban Rooftop Farm Adaptations case study for more information on urban agriculture.
- **Farmworkers**. Heat stress poses a serious risk to farmworkers in both rural and urban environments. With the observed and projected increases in both the frequency of very hot days and the duration and severity of heat waves,^33^ the risk of illness due to prolonged heat exposure is of growing concern. It is well known that heat exposure reduces the capacity for physical activity,^376^ but it also affects physiological and psychological health, safety, and socioeconomic well‐being.^342^, ^343^, ^344^, ^345^ Farmworkers may need to work fewer hours or shift their hours to limit heat exposure. On very hot days, workers may need to work indoors, which reduces productivity if certain outdoor farm tasks are regularly needed, such as scouting for pests and diseases. The issue of heat stress has direct impacts on productivity at the individual farm level, as well as on overall agricultural outputs and implications for employment.^377^ Shortened planting and harvesting windows for some crops due to current or expected weather conditions means there is pressure to get crops planted or harvested quickly. This pressure may lead to longer shifts for workers under periods of extreme heat, especially if workers do not want to miss work because of concerns over lost income or other factors.^378^ Outdoor workers have adapted various coping measures to deal with heat stress. Some of these strategies include using fans and other ventilation and cooling devices, taking breaks to hydrate and cool down, shifting working hours to avoid heat, and wearing appropriate personal protective equipment such as hats, thin clothing, and sunblock.^345^
- **Undocumented farmworkers**. It is estimated that more than 50% of farmworkers in New York State are undocumented.^355^ While these workers play a critical role in the success of agricultural productivity statewide, their precarious status makes them particularly vulnerable to climate‐related hazards. Within the state's dairy industry, farmworkers are exposed to difficult climatic conditions during the winter months or summer storms when outside work, such as caring for calves, is required.^379^ Many undocumented workers live in substandard housing that may not be able to withstand an extreme weather event. If housing conditions are made worse by a weather event, workers may be afraid to speak up because of fear of deportation and potential lost wages.^380^ The state's Farm Laborers Fair Labor Practices Act, which took effect on January 1, 2020, provides equitable pay, compensation, and other benefits to all farmworkers regardless of legal status.^381^ Despite the protections under the new law, all undocumented farmworkers still work on the “front line” when it comes to dealing with the effects of climate change.
- **Farmers of color**. In the Northeast, white landowners control nearly 100% of farmland and receive a majority of farmland assistance.^382^ Nonwhite producers make up only 1.22% of all farmers in New York State, and just 0.2% of the state's farmland is either owned or operated by Black or African American producers.^14^, ^383^ Since 1920, Black farmland ownership has declined across the United States, along with the possibility for wealth accumulation.^384^ Four major barriers to people of color entering into, expanding their position in, or remaining in agriculture in New York State are access to land; access to infrastructure and resources; access to education and training; and access to capital.^385^ These farmers also face increasing challenges to implementing adaptation strategies such as using soil health practices because their economic and farm viability is threatened by rising land costs; encroaching developments on viable farmland; continued discrimination and structural barriers; and more severe weather and pest pressures.^386^ USDA is administering grants to expand access to conservation assistance for CSA to farmers who are new to farming, low‐income, “socially disadvantaged,” or military veterans. Six projects targeting New York State producers received support in 2022.^387^
- **Indigenous farmers**. Across the United States, Tribal Nations are highly exposed to climate change risks and hazards, specifically extreme heat, decreased precipitation, and increased drought. In addition, historical policies of forced displacement have resulted in the loss of productive lands and forced relocation to lands with lower economic and agronomic value.^29^ For example, in 1846, one‐third of the Cayuga people were forced from New York State to Kansas.^29^ This displacement resulted in near total loss of land. Before colonization, the Oneida Nation exercised complete sovereignty over a territory that stretched across 6 million acres, with thousands of acres planted in corn, beans, squash, cucumbers, watermelons, and orchards.^388^, ^389^ Even after a vast reduction in the lands they hold, the Oneida—and all Indigenous Peoples—remain connected to the land. Tribal Nations across New York are engaged in agriculture today.
  In spring 2023, the Oneida Nation opened Wáhta’ Maple Farm on Oneida lands.^390^ Oneida citizens began producing organic maple syrup and have plans to plant hundreds of new trees. Additionally, the Oneida plans to construct a 50,000‐square‐foot cannabis cultivation and production facility,^391^ which will provide employment opportunities for the community. The revenue from both products will provide additional income streams and help buffer against climate uncertainties. The Seneca Nation has obtained regulatory authority over production of the emerging crop hemp on Seneca territory.^392^ This will allow the Nation to increase production of this crop, both for use within the community (as food, fuel, and fiber) and as an alternative income stream in the face of climate‐related stressors. Further, since the establishment of Gakwi:yo:h Farms by the Seneca, they have emphasized soil health through plantings of the traditional “three sisters” (corn, beans, and squash) and other best management practices,^393^ which increases their farm's resilience to heavy rainfall, drought, and other impacts of climate change. The Tuscarora Nation has followed traditional teachings that warned of unsettling environmental changes and has invested further in seed‐saving programs, food preservation, community‐supported agriculture, land mapping, and restoration efforts to both protect the People's food base and preserve culture in response to climate‐change threats.^394^ The Mohawk Nation (Akwesasne) acknowledges the need to use compost and other amendments to improve soil quality, invest in irrigation and drainage systems to manage soil moisture, plant perennials to combat soil erosion, use seed storage and exchange, and increase education about the importance of maintaining traditional Tribal practices in a changing climate.^208^ The Shinnecock have expanded into kelp farming and are working to expand their community garden (refer to the Shinnecock Nation Marine and Land Farming Adaptations case study for more information). The Onondaga Nation has prioritized the pursuit of Indigenous food sovereignty. On the 163‐acre Onondaga Nation Farm, the focus is on growing the three sisters as well as keeping bees, raising chickens and buffalo, and foraging for nuts, berries, and onions. The Farm hosts a weekly farmers’ market and offers programs to teach traditional ways of cultivation, cooking, preservation, and seed saving.^395^, ^396^
- **Veteran farmers**. In 2017, 9.25% of the 45,777 principal producers in New York State were military veterans.^397^ Veteran farmers are concerned about sustainability and resilience, and most recognize climate change as an important factor to consider for the sustainability of their farms (Cornell Farm Ops Program [2022, October, Personal communication]). However, many veterans entering into agriculture may have physical disabilities and/or may be dealing with post‐traumatic stress. Engaging with veterans as they leave service by exposing them to the various tools, resources, and programs around farming is critical to the success of their farm operations. Providing support is also critical, including training and education on climate change. If not prepared for the effects of climate change, these veterans could struggle more than others to sustain their operations (Cornell Farm Ops Program [2022, October, Personal communication]). Skills learned in the military, such as detailed planning, can help with creating and executing a farm plan and responding with resilience when something goes wrong.^398^ As noted above, USDA is administering grants to help military veterans and other specific population groups access conservation assistance for CSA.^387^
- **Amish and plain community farmers**. New York has the fastest‐growing Amish and plain community farming population in the country.^399^ As of 2017, there were 55 Amish settlements in the state with an estimated population of 20,000.^400^ These farming communities are particularly susceptible to climate impacts because they are generally made up of smaller farms that do not use modern technology, and almost all their income depends on agriculture. If a crop fails, it will cause significant impacts to their operation. Amish and plain community farmers face different risks because they generally do not depend on electricity or gas‐powered equipment that could be affected by extreme weather events; thus, their adaptation strategies could differ somewhat from those of “traditional” farms. Amish and plain community farmers might also have less access to credit and finance investments, and might have less access to information about climate‐smart farming or conservation practices.^401^ Providing education and support, including training and education on climate change, for Amish and plain community farmers will be critical for the sustainability of their operations.

### 4.2 Climate equity and justice considerations

The impacts of climate change on agriculture raise several environmental justice and equity issues regarding vulnerable populations. As described above, different people in New York State will experience climate change differently, depending on factors that make particular regions, communities, groups, and individuals more sensitive to harm from climate change and less able to cope and respond. Climate equity and justice considerations related to agriculture include economic disparities and lack of inclusivity. These factors compound the impacts of climate change and affect farmers’ abilities to adapt.

#### 4.2.1 Economic disparities

Many farming communities in New York State face high rural poverty rates; in 2019, the poverty rate of farming communities was 14.9%.^402^ Extreme weather events, which are becoming more frequent and severe under climate change, can damage infrastructure in rural communities, causing an increased burden on municipal and individual budgets that are already strained. Another pressure facing farmers is the high cost of farmland, which is increasing both nationally and across New York State, with the state's per‐acre values rising by 3.9% from 2020 to 2021.^403^ The high cost of land makes it harder for new and beginning farmers to enter into and remain in agriculture, and those new to the industry may also face concerns about climate change threatening the viability of farming as a livelihood. The 2017 median household income in New York State was $64,894.^404^ The income per farm averaged only $42,875 in New York State as of the 2017 Agricultural Census.^14^ Already, 48.6% of all family farms nationwide have a negative farm income, with 82% of total household income for farms coming from off‐farm jobs, and with smaller farms relying more on off‐farm income than larger farms to remain viable.^405^ As farmers continue to face decisions about making costly investments to adapt their entire operations to climate change, they may look more favorably on opportunities to use the land for an income other than agriculture—for example, by converting it to solar or wind power or development. Further, farmers who rent land rather than own it might not be able or willing to make costly investments in adaptation strategies, because the land is not theirs.

New York's Climate Justice Working Group, under the Climate Act, developed a set of criteria for identifying and mapping “disadvantaged” communities. According to these criteria, a community is considered disadvantaged based on a combination of environmental and climate risk burdens, demographic characteristics associated with climate vulnerability, and health vulnerabilities.^28^ As shown in Figure 3-3, there are many designated disadvantaged communities with high agricultural production, such as the towns of Amenia in the Hudson Valley, Dundee in the Finger Lakes region, and Medina in Western New York.

**FIGURE 3-3:**
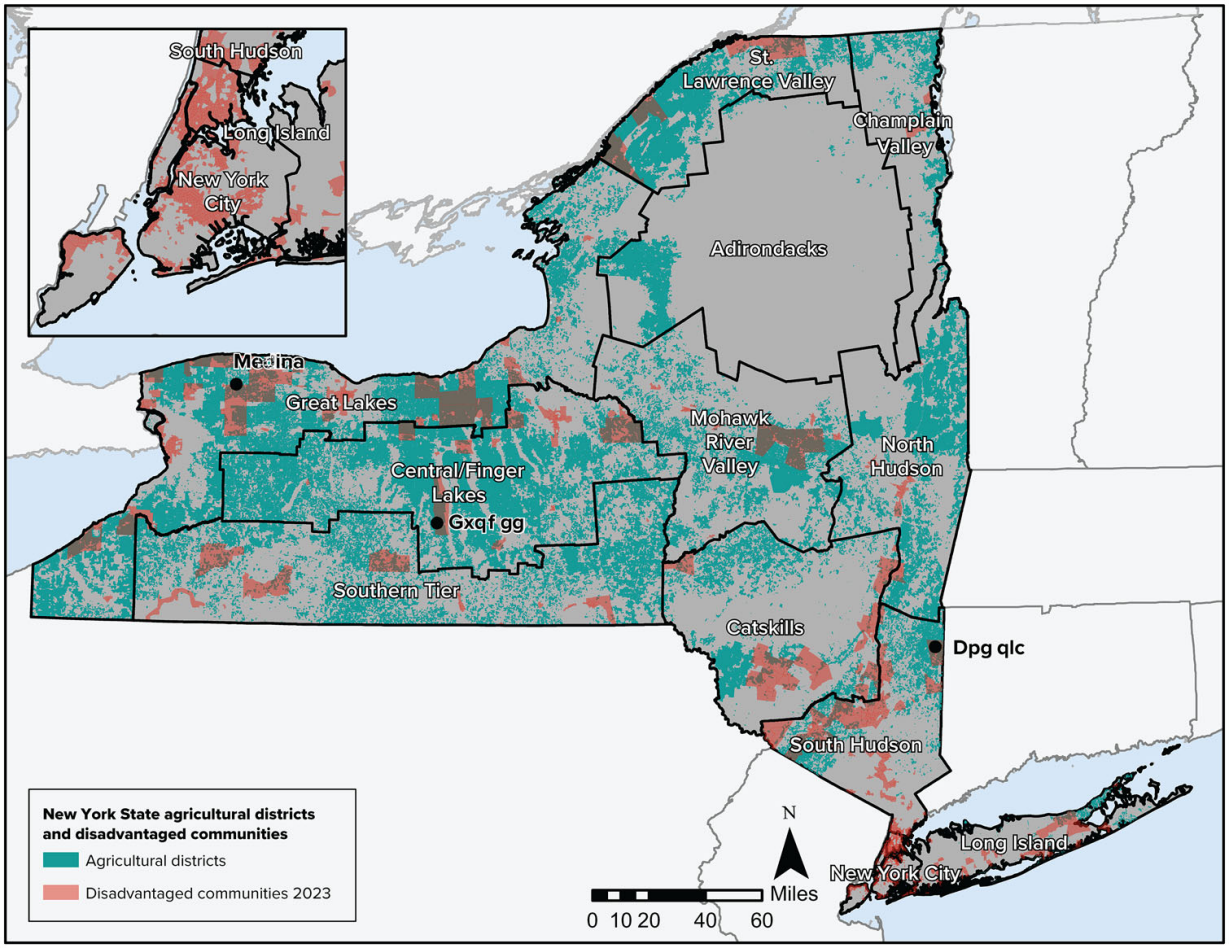
Disadvantaged communities overlaying New York State agricultural districts, 2023. Here, the “disadvantaged” designation is as statutorily prescribed by the Climate Act. Data from Cornell Institute for Resource Information Sciences and NYSDAM (2023)^406^ and New York State Energy Research and Development Authority (2023).^407^

These and other low‐income farm communities need support to allow them to adapt to climate change. New York State's Climate Resilient Farming Program provides competitive grant funds for farm projects that mitigate greenhouse gas emissions and enhance on‐farm adaptation and resilience.^7^ However, this program has not had enough resources to fund all applications for cost‐share grants. There also may be a lack of awareness of this funding opportunity because funds are applied for and awarded to county Soil and Water Conservation Districts on a farmer's behalf. Of the funded applications, there has not yet been an analysis conducted of the proportion of the funding that has gone to low‐income or disadvantaged communities.

#### 4.2.2 Lack of inclusivity

In 2021, NYSDAM issued the *Diversity and Racial Equity Working Group Report*,^385^ which sought to understand the historical circumstances that led to discrimination against farmers of color and to make recommendations for addressing these injustices. The Working Group also sought to increase the numbers of farm owners and farmers of color, along with the support provided to them. While the report did not explicitly address climate change, many of its findings and recommendations relate to the compounding effects of climate change and the need for an equity and justice lens in terms of agricultural climate adaptation.

The demographic composition of New York State's farmers (Table 3-4) is much less diverse than the overall state population. Altogether, American Indian, Alaska Native, Asian, Native Hawaiian and Pacific Islander, Black, and biracial farmers represent only 1.2% of New York State farmers. Military veterans represent 8.3% of New York State farmers, and women represent 37.8% of farmers (as estimated using data from Table 3-4). There are no current data on farmers and farmworkers who identify as LGBTQ+; however, a 2019 report found that 3–5% of rural Americans nationally identify as LGBTQ+,^408^, ^409^, ^410^ as does 6% of the workforce in New York State.^411^, ^412^

**TABLE 3-4:** Farmer characteristics for New York State as of 2017.

Producer characteristics	Number
Race	
American Indian or Alaska Native	125
Asian	166
Black or African American	139
Native Hawaiian or other Pacific Islander	40
White	57,155
More than one race	240
Ethnicity	
Producers of Hispanic, Latino, or Spanish origin	606
Age	
<35 years	6026
35–64 years	34,889
65 years and older	16,950
Sex	
Male	35,985
Female	21,880
New and beginning farmers	15,602
Military service (veteran or active duty)	4806
LGBTQ+ producers	No data
Total producers	57,865
**Income**	**Amount**
Average net cash income per farm	$42,875
Median household income in New York in 2017	$64,894

*Note*: Data for all characteristics other than median household income from USDA, National Agricultural Statistics Service (2019)^14^; median household income from Guzman (2018).^404^

Recent research has found that farmers of color, immigrant farmers, and female farmers typically have smaller farms and grow higher‐value, more labor‐intensive agricultural products, but have received less government support.^413^ The term BIPOC refers to Black, Indigenous, and People of Color. BIPOC farmers face additional challenges, including a lack of capital to make improvements to their farm, a lack of land security, and a lack of access to family land or wealth.^413^ Climate change can exacerbate these existing challenges because BIPOC farmers have less capacity to adapt following an extreme weather event.

The *Diversity and Racial Equity Working Group Report* included recommendations to help bring inclusivity to New York State agriculture by providing greater access for BIPOC farmers in four key areas: infrastructure and resources; education and training; land; and capital. A fifth key area focused on NYSDAM departmental reforms. Of the 21 recommendations in the report, many have a connection to climate change, as shown in Table 3-5.

**TABLE 3-5:** Summary of recommendations from the Diversity and Racial Equity Working Group Report and the potential connections to climate change and agriculture.

Summary of recommendations by key area	Potential connections to climate change and agriculture (authors’ recommendations)
**Key area: Access to infrastructure and resources**	
Provide funding to support innovative, technological, and regenerative agriculture practices.	Innovative, sustainable agriculture practices can help increase resilience to climate change.
Identify funds available for infrastructure and improving operations.	Farm infrastructure projects that increase efficiency or use modern technologies can improve the resilience of farms.
**Key area: Access to education and training**	
Create an agricultural training program, and networks for employment opportunities in agriculture.	Agricultural training programs and networks could also include a focus on CSA and employment opportunities.
Review agricultural education curriculum to ensure that there is BIPOC representation and a racial and income equity framework.	Ensure that agricultural education curriculum has a focus on BIPOC representation and a racial and income equity framework as it relates to climate justice.
Encourage pathways to recruit more BIPOC students in agriculture.	Include innovative CSA career options (e.g., precision agriculture or traditional farming methods) that can entice BIPOC and young people to farming.
**Key area: Access to capital**	
Develop partnerships and structured conversations with lenders to better serve BIPOC farmers.	Provide greater outreach about available lending and grants for CSA to BIPOC, low‐income, and young farmers.
Provide funding for grant programming that covers operational costs.	Provide funding for CSA practices and operational costs of infrastructure upgrades.
Expand state procurement to ensure BIPOC farmers can sell to institutions like schools, hospitals, and so on through value chain coordination.	Prioritize selling sustainably produced products to institutions.
**Key area: Access to land**	
Encourage Empire State Development to administer the Regional Revolving Loan Trust Fund to better serve BIPOC producers and business owners.	Ensure loans are available for high‐quality farmland that is more resilient to climate change.
Encourage the development of and support existing urban land trusts to protect land that can be used to produce food in historically under‐resourced communities.	Provide support for sustainable, climate‐resilient agricultural production that is grown in urban centers, closer to population centers.
Provide funding to support the direct purchase of land by BIPOC farmers.	Ensure new land purchases and new farm businesses are supported to use CSA practices.
Develop an Access to Land Toolkit that includes guidance on purchasing and protecting land.	Include criteria for consideration of factors that make farmland more resilient (floodplains, access to water, etc.).
**Key area: NYSDAM reforms**	
Establish dedicated staff and communication channels to address BIPOC‐specific issues and needs to ensure BIPOC farmers and leaders feel safe, comfortable, and welcomed in agricultural spaces.	Include a specific focus on climate change, climate justice, and equity in agricultural spaces.

*Note*: Table adapted from NYSDAM (2021).^385^

Abbreviations: BIPOC, Black, Indigenous, and People of Color; CSA, Climate‐Smart Agriculture; NYSDAM, New York State Department of Agriculture and Markets.

The Diversity and Racial Equity Working Group proposed a $10 million investment to implement the recommendations in their report.^385^ The Working Group specifically encouraged investment in BIPOC‐led farms and organizations. These investments could focus on agricultural mitigation and adaptation projects in overburdened, underserved communities. By making resources available to implement these recommendations, New York would make progress toward the state's commitments to address the climate inequities and injustices in agriculture statewide and would ensure that all farmers engaged in agriculture production in New York State have the opportunity to adapt and become more resilient in the face of climate change.

New York State's Climate Act requires that disadvantaged communities, as defined by statute, receive at least 35% of the benefits of state investments, with a goal of 40%.^414^ Policymakers and agricultural organizations can help ensure that state spending for agricultural adaptation and mitigation is happening in these communities and will help increase diversity in farm ownership. Policymakers and agricultural organizations can also help ensure that BIPOC farmers have access to the infrastructure and resources, capital, and education and training needed to adapt to climate change, and that they have access to high‐quality land that is less vulnerable to the impacts of climate change.^385^

### 4.3 Agricultural systems at risk

According to the International Food Policy Research Institute, “food systems are the sum of actors and interactions along the food value chain—from input supply and production of crops, livestock, fish, and other agricultural commodities to transportation, processing, retailing, wholesaling, and preparation of foods to consumption and disposal.”^415^ The COVID‐19 pandemic brought the frailty of the nation's food system and supply chain to the forefront of everyday American lives. For example, minor disruptions in the workforce led to chokepoints in meat and dairy processing, where short‐run disruptions caused ripple effects throughout the supply chain. Climate change can be regarded as a compounding factor^22^ that can have cascading impacts on food systems, ecological systems, and landscapes.

There are many recent examples of climate impacts on the food supply chain in New York State. Recent extreme weather events, such as Superstorm Sandy in 2012, created huge disruptions in the food system and the agricultural supply chain statewide because of damage occurring at multiple points in the chain, from production (e.g., crop loss at the farm level) through the transportation chain (e.g., supply of fuel) to critical distribution points (e.g., retail outlets).^416^ Disruptions like these disproportionately affect low‐income and other vulnerable populations because of increases in food prices and the need to travel greater distances to get food.^417^ The Hunts Point neighborhood in the South Bronx is one of the largest food distribution areas for New York City, distributing 4.5 billion pounds of food each year. Hunts Point is located on a peninsula and, therefore, is susceptible to flooding and major disruptions, as occurred during Superstorm Sandy.^417^, ^418^ The storm affected power, transportation, fuel, and telecommunication networks, all of which are needed for the food supply chain.^417^ Hunts Point is also a majority non‐white neighborhood, with high levels of food insecurity and a poverty rate of 39.6%.^419^ During and after the storm, residents were unable to access markets elsewhere due to flooded roads and subway stations.^417^ This case serves as an example of how extreme weather events can affect vulnerable systems (i.e., a centralized food supply system) and vulnerable populations to a greater extent.

The dairy supply chain is another example of a system particularly vulnerable to climate change.^420^ Cows produce milk every day, often in rural areas connected by small roads. When an extreme rainfall or snowfall event renders roads impassable, milk trucks often cannot get to farms. This forces farmers to dump milk. In November 2014, a “snowmageddon” event hit the Buffalo area; the region received 5–8 feet of snowfall, causing farmers to dump milk that trucks could not pick up.^361^ During the storm, a single farm dumped about 6000 pounds of milk, or 750 gallons, costing $1500 in lost revenue.^421^ In fact, many farms have dumped far greater amounts of milk during similar extreme weather events. Refer to the Transportation chapter for more information on how climate hazards can affect the movement of agriculture products and other goods on roads and highways.

New York State's agricultural systems are also connected to regional and national food systems; therefore, climate disruptions to agricultural commodities in other regions will affect costs and production in the state. Such disruptions can sometimes create the need—or opportunities—for increased production in New York State and other states in the Northeast.^71^ For example, California is the largest producer of agricultural goods in the country but has been affected by sustained severe drought. In 2021, as a response to the ongoing drought, California farmers planted fewer tomatoes and grapes than usual, affecting supplies and prices for these important commodities.^422^ Even though California remains the nation's top dairy‐producing state, its dairy farmers are facing increasing challenges from heat stress, drought, and water access. These factors have contributed to rising costs that are shuttering some California dairy farms,^423^ but may create market opportunities for farmers in the Northeast.^71^

Agriculture is also part of a larger and complex ecological system, and climate impacts to the ecological system or landscape can directly or indirectly affect agricultural production. For example, due to climate change, the life cycles of native or managed pollinators that are critical for both agriculture and broader environmental services are becoming out of sync with the flowering of important food crops.^287^, ^424^ Bees can emerge at different times depending on air temperature, and crops flower at different times due to changes in snow melt or temperature.^424^, ^425^ In response, researchers suggest taking an integrated landscape approach that combines policy and practical strategies for multiple land systems (such as agricultural systems, ecological systems, and forest systems) within an area to ensure sustainable and equitable land use that is resilient to climate change.^426^ Refer to the Ecosystems chapter for more information on asynchrony in plant–pollinator mutualism.

### 4.4 Multihazard and nonclimate stressors

New York State's farmers face many challenges and stressors that are not directly related to climate change, such as tight profit margins and labor shortages. Climate change impacts can exacerbate existing climate hazards and nonclimate stressors, resulting in consequences that are often more severe than those resulting from a single hazard.^427^ For example, extreme heat and drought can compromise water supplies, in addition to directly affecting crops and livestock. Below is an overview of several key nonclimate factors that could potentially be compounded by climate impacts.

- **Labor costs and supply**. Labor represented the highest production cost (20%) for New York State's farms in 2017.^428^ Within dairy, the state's largest commodity sector, labor represents about 17% of total costs.^429^ New York's farms are relatively small and more diversified than farms in other parts of the country, such as the Midwest.^115^ The specialization in dairy and horticulture crop production means that New York State farmers also hire relatively more labor, which renders the state's agriculture system vulnerable to shortages in the labor supply. The higher productivity of workers in nonfarm activities means that farmers face stiff competition and must offer relatively high wages.^430^, ^431^ Additional labor requirements on hired farm labor, such as paid family leave and removal of overtime pay exemptions, also contribute to increasing labor costs.^432^ Importantly, agriculture is a seasonal activity in New York State, with peak periods of necessary labor (e.g., planting and harvesting), which complicates hiring. In addition, changes in immigration policies and enforcement influence the labor supply of foreign (often seasonal) workers.^433^ Immigration challenges drive up labor costs, which are particularly challenging for smaller farms with smaller operational margins. Climate change can exacerbate labor issues. For example, the increase in extreme rainfall events has shifted windows when it is possible to get onto fields for planting or harvesting.^434^ Farmers may have to pay higher labor costs, including overtime, or may not have access to enough labor at critical times of the growing season. The need for climate adaptations, such as using more precision seasonal forecasting tools, could require a more highly trained workforce. Farmers might have trouble finding highly trained workers or might struggle to pay the higher wages these workers could demand.
- **Land acquisition**. Land acquisition is an ongoing challenge for New York State farmers, particularly BIPOC farmers and those who are new to farming. The average per‐acre cost of land in New York is high. In 2021, the average cost was $3270 per acre, a 3.8% increase from 2020.^158^ Land cost is a prohibitive factor for new farmers looking to join the profession, and available land is often of lower quality, with poor soil health, creating challenges for farmers.^435^, ^436^
- **Other competing land uses**. Agriculture competes with other land uses, including urban and suburban development and, increasingly, renewable energy development. While current landowners selling their properties can benefit from potential windfall gains, these competing nonfarm land uses make land less affordable to farmers seeking to acquire new land and may affect decisions, like whether to buy or rent.
- **Broadband access**. Many agricultural producers in rural areas lack access to broadband (which includes cable/fiber/DSL internet access)^437^ and face limited cell phone service. This limits access to many digital and precision agricultural technologies, as well as to an economy that is ever more dependent upon online transactions and marketing, hindering economic growth. However, new technologies such as low‐power wide‐area networks, which do not require broadband and are cheaper than traditional mobile networks, might provide rural farmers with more technological opportunities in the future.^438^ New technologies are under evaluation across the state.^439^
- **Supply chain disruptions**. Several factors affect the overall agricultural supply chain in the state and beyond. Most recently, the COVID‐19 pandemic, the war in Ukraine, and other global disruptions have exacerbated existing agricultural supply chain challenges. For instance, these shocks have put a spotlight on the potential overdependence on more consolidated processing and distribution centers, highlighting the need for more robust local and regional food supply chains. The COVID‐19 pandemic had a large effect on farm operations across the country, including those in New York State. For example, 65% of the state's farmers reported that the pandemic negatively affected their business.^440^ The pandemic disrupted the milk supply chain and transportation, which led to milk dumping, affecting farmers’ finances and mental health.^441^, ^442^, ^443^
- **Market structural changes**. Important market structural changes are occurring in the production and marketing of agricultural products and in the overall food supply chain. There is a nationwide trend toward fewer and larger farms.^444^ While this consolidation trend varies considerably by commodity and region, and is more pronounced in the Midwest, it is still evident in the Northeast and New York State. (For instance, refer to the discussion about dairy industry consolidation in Section 3.3.4.) Diversifying into new and value‐added crops and products (e.g., kelp, alcoholic beverages, and yogurt) can help some farmers increase operational resilience and, as a result, better withstand weather and market variability.
- **Food retailer consolidation**. Another important trend is the consolidation of food retailers and the rise of food services.^445^, ^446^, ^447^, ^448^, ^449^ A parallel trend is the rise of local food markets and agricultural and food cooperatives,^450^ which could be a response to the consolidation of food retailing and changing consumer demands. Agricultural cooperatives are also consolidating, thus declining in number while increasing in size.^447^ These cooperatives allow farmers to pool resources and gain more market power to secure more stable and higher prices for their products. Taken together, these trends reflect changing market structures in agricultural and food markets that could either exacerbate or mitigate the influence of extreme weather arising from a changing climate.
- **State and federal regulations**. Finally, New York farmers often mention compliance with the plethora of state and federal regulations regarding agricultural production as one of the biggest challenges they face as business owners.^434^ These include laws and regulations regarding labor (e.g., overtime hour restrictions), chemical handling, air emissions, farm safety, pesticide use, and water use.^451^ While these regulations aim to protect farmworkers, who can be a vulnerable population, complying with the regulations often requires a full‐time employee at larger farms and can be difficult for smaller operations to navigate. Climate change can make compliance even more challenging. For example, extreme weather events can affect water and waste handling or shorten windows for operations, requiring farms to pay overtime hours to complete farm work when necessary. The New York State Department of Labor recently approved reducing the state's overtime threshold to 40 h per week by 2032, which agricultural organizations say could severely affect farms that are already economically stressed.^452^

These factors are interconnected issues that affect agricultural production in New York State, with climate change acting as a compounding factor.^22^

## 5 BROADER ADAPTATION CONSIDERATIONS

As discussed in Section 3, the climate change impacts on New York agriculture vary substantially depending on factors such as farm size and type, commodities being produced, and geography (region, specific landscapes, microclimates). As a result, farmers must tailor the adaptation practices and strategies implemented on their farm to match farm‐specific production systems.^71^, ^72^, ^73^ Section 3 introduced several examples of adaptation measures that the state's farmers can take to respond to specific impacts. This section expands on that earlier discussion by presenting additional adaptation measures, discussing resources and models available to support adaptation and resilience in New York's agriculture sector, and assessing what is known about the adoption and efficacy of these practices and tools to date.

There is a great deal of complexity associated with farmers’ decisions about whether to undertake an adaptation measure or whether to instead assume the risks of climate change. It is not always clear what the concept of climate change adaptation means to farmers.^72^ Some factors that contribute to a farmer's decisions about adaptation include the farmer's biophysical situation (e.g., healthy soil, access to adequate water)^453^; sociocultural identity (e.g., place identity, group identity, and social constructs)^454^, ^455^; values and beliefs (e.g., political beliefs)^456^; number of years in farming (e.g., age of farmer, generational influence)^457^, ^458^; and general perception of the risks associated with climate change.^459^ Indigenous communities may prioritize adaptation strategies that best align with their sense of place and longstanding cultural traditions.^455^

1 We got to keep working at it, we can't give up. This is the most important thing facing us today.

—Ted Furber, apple grower, Wayne County,

New York (July 2022)

Multiple surveys of farmers from New York State to the Midwest indicate that how farmers perceive anthropogenic climate change greatly influences whether they choose to implement mitigation and adaptation strategies on their farms.^72^, ^434^, ^460^, ^461^, ^462^ The evidence shows that most New York State farmers understand that the climate is changing, and many have started making changes to their practices and operations. For example, a 2019 statewide survey of 524 farmers found that 94% understand that the climate is changing, and 83% had experienced extreme weather events that affected their own farm in the previous 5 years.^463^ The survey also found widespread misunderstanding about the causes of climate change,^9^ which is consistent with the results of a study conducted across 12 midwestern states that found that farmers retain a high degree of uncertainty about the causes of climate change or the need for action.^464^

Although most New York farmers acknowledge and understand climate impacts to their farm operations, they need support to adapt to the impacts. In a 2019 New York survey, 57% of respondents said they do not have the financial capacity to deal with climate‐related risks.^9^ A farmer's decision to implement an adaptation action must be farm‐specific or made at the field level.^72^, ^434^ Farmers need specific information on how climate change affects their farms, as well as training on how to adapt.^9^ They need to understand site‐specific adaptation strategies, and they need access to technical assistance and financial resources to implement the strategies.^465^ This access to information and resources is a matter of equity (refer to Section 4). The remainder of this section describes resources that could help the state's farmers adapt.

### 5.1 The Climate‐Smart Agriculture framework

CSA is an integrated approach to transforming agricultural systems to increase sustainability and ensure food security under a changing climate. The United Nations Food and Agriculture Organization champions CSA, with many organizations and farmers adopting the approach around the world.^6^ CSA provides a framework for understanding how to integrate climate change considerations into the planning and implementation of sustainable agriculture strategies to ensure nutrition, food security, and resilience, while also mitigating the impacts and drivers of climate change. The three pillars of CSA (Figure 3-4) focus on: (1) sustainably and equitably increasing agricultural productivity and incomes; (2) adapting and building resilience to climate change; and (3) reducing and/or removing greenhouse gas emissions, where possible.^6^ As noted in Section 2, there are often important synergies between agricultural adaptation and mitigation practices; farmers should not consider these responses in isolation and should prioritize practices that can contribute to both goals when possible.^466^ CSA has also become a top priority for the USDA, highlighted in the 2021 *Climate‐Smart Agriculture and Forestry Strategy: 90‐Day Progress Report* ^467^ and in the 2021 USDA Partnerships for Climate‐Smart Commodities Program.^468^ Adoption of CSA practices among New York farmers has been limited to date. There are several reasons for these low rates of adoption, including uncertainty, lack of knowledge or skills, and lack of finances to make changes. Many New York State farmers are struggling financially with high production costs and low returns.

**FIGURE 3-4:**
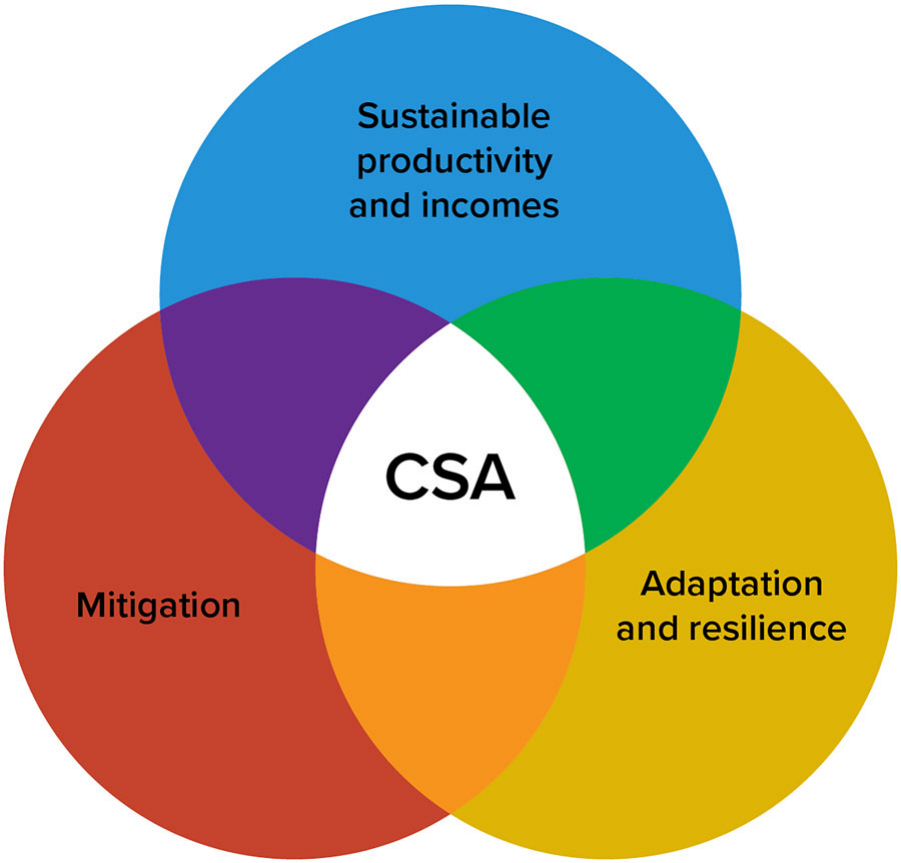
The three pillars of CSA. Adapted from Food and Agriculture Organization of the United Nations (2013).^6^ Abbreviation: CSA, Climate‐Smart Agriculture.

### 5.2 Information and technology

Farmers make decisions based on their experience and the information they have on hand; they often obtain this information through trusted relationships with their peers, extension specialists, or consultants. Ensuring that farmers have access to research‐based information on current and future farm‐specific climate impacts and site‐specific adaptive measures is key to enhancing climate adaptation and farm resilience. Farmers need information about how different varieties or breeds fare under various climatic conditions, and detailed localized information about likely climatic distributions during the life of their investment. For instance, planting tree crops is a long‐term investment that may be critically affected by a changing climate. The combination of these two types of data provides the necessary insights for farmers to make sound long‐term decisions.

The rise of precision agriculture is typically perceived as a way to improve farm management and increase the efficiency of inputs, such as optimizing fertilizer applications through the use of variable rate management.^469^, ^470^ Over the last 10−20 years, farmers across the United States have adopted precision agriculture technologies to varying degrees to boost productivity and profitability. Because New York State's field crop farms tend to be smaller in acreage than their midwestern counterparts, adoption of such cutting‐edge technology lags slightly behind on the vast majority of field crop acreage in the state. However, New York has many innovative farmers who have adopted, and will continue to adopt, technologies proven to be cost‐effective.^471^ Such technologies may include precision planters that can prescriptively determine seeding rates and fertility needs as a planter moves across thousands of acres, as well as targeted pesticide applications based on satellite or drone imagery and field history. These tools and adaptations help farmers make prescriptive decisions that reduce inputs and minimize losses, thereby increasing efficiency, profits, and sustainability.

Online decision‐support tools that provide accurate farm‐level weather and climate data related to agricultural decisions can also aid farmers in making more informed decisions and provide economic benefits.^32^, ^124^ Such tools include the Network for Environment and Weather Applications (NEWA) IPM tools and the Cornell Climate Smart Farming online toolkit. NEWA users reported saving on average $19,500 per year in pesticide use and preventing up to $264,000 per year in crop loss as a direct result of using pest forecast systems.^472^

The usefulness of these technologies will depend on whether farmers can adjust their practices based on the information provided. For instance, a farmer growing a rainfed cereal crop may have limited options to adapt to a mid‐season drought, even if they have access to data about the potential impacts of that drought. However, a dairy farmer may be able to change feed composition and heat management strategies in response to an incoming heat wave. The rise of precision agriculture and other technological tools to enhance farmer decisions will require sustained efforts between farmers and specialists to codevelop new tools that will be most helpful to farmers, and to train them in their use.

### 5.3 Soil health

Soil health is “the continued capacity of the soil to function as a vital living ecosystem that sustains plants, animals, and humans.”^473^ Building and maintaining healthy soil has become a key adaptation strategy for farmers across New York State and beyond. Intensive agricultural practices (such as excessive tillage) and poor soil management degrade soil quality, resulting in decreased potential farm profitability and sustainability. Fortunately, interest in soil health has expanded greatly over the last decade as techniques to measure, monitor, and communicate its benefits have improved.^474^

1 The number one adaptation tool for farmers at the present time is cover crops—it makes the most difference right away quickly. I only wish we could get more crop farmers to grow cover crops.

—Dale Stein, dairy farmer, Monroe County,

New York (August 2022)

Soil health takes an ecological‐systems approach to soil management. It integrates the biological, physical, and chemical components of the soil and encourages the implementation of many different practices. While the individual practices implemented on any given farm will differ, some soil health practices include cover cropping; soil amendments; diversified crop rotations; integration of livestock on the land; rotational grazing; conservation cover; forage planting; buffer strips; double cropping; and no/reduced tillage. Proper management plans for nutrients (including manure) and pests are also critical when it comes to improving soil health and providing other natural resource benefits. A key component of healthy soils is organic matter.^100^ Adding organic matter to soil increases a farm's resilience to both drought and flooding by providing greater water‐holding capacity and better drainage, while also supplying energy for robust microbial communities that improve the efficiency of nutrient availability to crops. Healthy soils are less susceptible to erosion, runoff, and compaction, particularly when the soil is covered year‐round (e.g., cover crops are grown when no cash crop is growing). Cover crops improve soil properties such as aggregate stability, which is the ability of a soil to resist falling apart when wetted and hit by raindrops. This means sediments and nutrients remain on farms during extreme weather events instead of entering water bodies and causing water quality impairments and potentially HABs.

Farmers are increasingly using commercially available soil health tests, such as the Comprehensive Assessment of Soil Health test, to quantify the impacts of management practices on their farms and to visualize which soil parameters are constraining crop productivity.^475^ Adoption of practices that build soil health has increased in New York State. In 2017, farmers planted 295,433 acres in cover crops, excluding Conservation Reserve Program acres, and implemented no‐till practices on 337,968 acres, an increase of 37% and 21%, respectively, since 2012.^14^ In addition, farmers also participate in Natural Resource Conservation Service (NRCS) financial assistance programs that promote soil improvements and other conservation practices, such as the Environmental Quality Incentives Program.^476^ Communicating the economic, environmental, climate, and productivity benefits of implementing soil health practices on New York State farms is critical for promoting greater adoption of these practices statewide.^477^ Furthermore, research is ongoing to provide the state's farmers with target soil health levels that are realistic benchmarks to achieve within the context of their unique production environment (i.e., soil type, cropping system, and geographic region).^478^

Soils are the largest terrestrial sink of carbon; therefore, maintaining healthy soils and restoring degraded soils in ways that increase soil organic carbon and enhance carbon sequestration is also a key climate mitigation strategy.^479^ Biochar, a carbon‐rich, charcoal‐like material made through pyrolysis of organic waste materials and intended for use in agricultural and environmental applications, is a carbon‐negative technology.^480^ It has potential benefits for crop yields, water‐use efficiency, nutrient retention, and microbial activity, among many other benefits.^481^

### 5.4 Public policies

Public policies play a critical role in influencing adaptive capacity. Adaptation by small farmers will likely require active support in the form of grants, financing, and other financial incentives. There are also potential synergies in coupling carbon sequestration efforts and payments with adaptation strategies. At the same time, policymakers should avoid developing policies that create future obstacles to adaptation. For instance, policies that create exclusive markets for certain farm products can lead to the emergence of vested interests, which can become an obstacle to reforms and policy changes that promote more climate‐resilient farming. One study found that increasing regional specialization in certain commodities in U.S. agriculture, particularly in the Midwest, has led to greater dependence on field crops, which increases the sensitivity of the region's agriculture to climatic extremes (i.e., monoculture vs. diversified crop production).^295^ Public policies also play a central role in making certain types of agricultural activities more profitable, which in turn influences the degree of specialization farmers undertake.

#### 5.4.1 State policies

New York State has two main mechanisms to protect and promote the availability of land for farming: the Agricultural Districts Law and the Farmland Protection Program. The Agricultural Districts Law not only protects farmland in agricultural districts but also allows for reduced property tax bills for land in agricultural production by limiting the property tax assessment of that land. To qualify for the reduced assessment, a parcel must consist of (generally) seven or more acres that have been used in the preceding 2 years for the production and sale of crops, livestock, or livestock products. Further, the annual gross sales of agricultural products must average $10,000 or more for the preceding 2 years. However, if an agricultural enterprise is less than seven acres, it may qualify if the average annual gross sales are at least $50,000. The Agricultural Districts Law can be thought of as a resilience program for communities to preserve working lands and maintain local food production. New York's Farmland Protection Program provides farmland planning grants, farmland protection implementation grants, and land trust grants. These grant programs provide local governments and land trusts with funding opportunities and resources to help plan and preserve farmland in their communities.

Many other state programs and initiatives support farmers’ adoption of conservation and climate adaptation and mitigation practices. These include the New York State Climate Resilient Farming Program, the Climate Smart Farming initiative at Cornell University, the passage of the New York State Soil Health and Climate Resiliency Act, and several other conservation and renewable energy programs in the state's Environmental Protection Fund and elsewhere. Funding to farmers from NYSDAM, distributed through the Climate Resilient Farming Program, more than tripled in 2022 to $16.75 million. This funding helps provide farmers with the technical and financial assistance needed to implement projects that reduce greenhouse gas emissions, build soil health, and improve water quality.^7^ Passage of the New York State Soil Health and Climate Resiliency Act in 2021 was momentous for stakeholders across the state as it established programs to assist all farmers in improving the health of their soil. It also supports climate‐resilient farming efforts to assist farmers in mitigating and adapting to the impacts of climate change.

#### 5.4.2 Federal policies

The Federal Farm Bill contains most agricultural programs, from traditional commodity‐support programs to disaster programs to what are referred to as conservation programs—which include the Environmental Quality Incentives Program, the Conservation Stewardship Program, and others that provide financial and technical assistance through the NRCS. Traditional commodity programs are not specifically geared toward providing coverage due to climate impacts, but they can help farmers whose yields have been affected by climate variability. The Noninsured Crop Disaster Assistance Program and Livestock Indemnity programs, also included in the Farm Bill, provide disaster assistance for losses, including those due to extreme weather events or conditions.

Federal conservation programs under the Farm Bill aim to help farmers make and maintain improvements to their lands to address water and air quality concerns, conserve groundwater and surface water, increase soil health, reduce erosion and sedimentation, improve and create wildlife habitat, and build resilience to increasing weather volatility. The Congressional Budget Office has assessed the programs in the Farm Bill with mandatory spending (which include nutrition programs as well as commodity‐support programs, conservation programs, and crop insurance) to cost $867 billion over federal fiscal years 2019–2028. Building on conservation programs contained in the Farm Bill, USDA announced a plan in October 2021 to integrate climate adaptation into its mission and programs in response to President Biden's Executive Order 14008, Tackling the Climate Crisis at Home and Abroad. A similar emphasis also occurred in 2014 in response to President Obama's Executive Order 13653, Preparing the United States for the Impacts of Climate Change.

#### 5.4.3 Research and development policies

Public policies can also help to promote beneficial actions by other organizations in the agri‐food supply chain. For instance, the research and development (R&D) underlying the breeding of climate‐resilient varieties or breeds is carried out “upstream” by researchers in private and public institutions. While these R&D activities implicitly consider changing climatic conditions over time, the process is slow and may not fully harness the available information regarding future climatic conditions. Researchers are often limited by the lack of support for long‐term research or funding for basic applied research that would provide data on best practices for climate resilience in agriculture. That is, R&D activities may not be forward‐looking enough to compensate for the rapid pace of climate change. Actions that reduce the cost of innovation or commercialization and that speed up R&D might be fruitful. Recent developments in genetic engineering (e.g., CRISPR) present new opportunities, though these also face limitations when seeking to enhance complex attributes involving numerous genes. The public sector plays a critical role in conducting R&D that may not be otherwise carried out by private actors given marketing or market volume considerations.

### 5.5 Additional financial opportunities from greenhouse gas mitigation

While not the primary focus of this assessment, efforts to mitigate greenhouse gas emissions could present financial opportunities for farmers. Income from solar power production and carbon markets could help to supplement farm income and offset some of the cost of coping with and adapting to a changing climate.

#### 5.5.1 Solar/agrivoltaics

The number of large‐scale solar projects in the United States has expanded rapidly and will continue to expand due to falling costs, federal and state incentives for solar energy, and state‐level renewable energy requirements. For example, the USDA's Rural Energy for America Program (REAP) offers $50 million annually to help farmers incorporate renewable energy and energy efficiency on their farms. A majority of REAP funding has gone to farmers looking to incorporate energy efficiency in operations. States also continue to increase their renewable energy portfolios by requiring that energy production comes from a mix of renewable energy sources^482^; solar will play a key role in New York State to meet the ambitious clean energy targets listed in the state's Climate Act.

There were 2493 solar installations on New York State farms in 2017, up from 815 installations in 2012,^14^ and it is likely that the number of installations has substantially increased since 2017. The Climate Act and related climate legislation, along with dramatically reduced costs for solar panels, has led to rapid growth of proposed utility‐scale solar projects in New York. Some of the earliest projects have now been permitted and are under construction, with the leading edge just beginning to come online. Solar currently provides around 3% of the state's energy, with two‐thirds of that coming from systems with capacities of less than 1 megawatt.^483^ The New York State Energy Plan suggests that by 2030, solar could provide up to 29% of the state's electricity, although estimates vary.^484^ Rural western, central, and northern counties of the state have the most potential for large‐scale solar facilities. Although many factors influence the siting of solar facilities, agricultural land is frequently involved because it offers many of the characteristics needed for solar installations (e.g., cleared land, relatively flat areas, access to power lines).^485^, ^486^ Although agricultural land is desirable for utility‐scale solar facilities, it is an increasingly controversial location for them as well.^487^

With large‐scale solar installations becoming increasingly feasible, farmers and landowners will have more opportunities to convert their land to solar energy production. However, solar energy production will have significant impacts on rural agricultural operations and land availability for agricultural production. While the footprint of solar installations continues to shrink with advances in technology and efficiency, solar energy production remains comparatively land‐intensive. Newer facilities require about 5 or more acres of developed land per megawatt of capacity, and multiple facilities of 350 megawatts or more are in the planning stages. Affordable agricultural land near three‐phase power lines tends to be desirable for solar facilities.^485^ Agrivoltaics, or colocation of agricultural activities with solar facilities, holds promise as a way for energy and food production (e.g., sheep grazing) to be integrated,^192^ but more research is needed to fully understand the extent to which agrivoltaics will be technically, agronomically, economically, and socially scalable (Kay D, Senior Extension Associate, Cornell University [2022, August, Personal communication]). Landowners will need to make smart land‐use decisions that consider tradeoffs. As with previous situations where nonagronomic energy technologies (e.g., oil, gas, and wind) modified farmland use to generate new income streams, the outcomes for rural land use, agronomic practices, and the farm economy will depend on numerous factors.^488^, ^489^

While solar power is needed to meet the renewable energy targets outlined in the Climate Act, the siting of solar facilities must be done thoughtfully and must consider the potential impacts to agricultural lands, as well as to other sensitive lands (refer to the Ecosystems chapter for more details). American Farmland Trust (AFT) released a smart solar siting report that provides a pathway toward expanding the use of solar to achieve New York's ambitious climate goals while minimizing solar development on prime agricultural lands.^490^ The New York State Energy Research and Development Authority is piloting a Smart Solar Siting Scorecard that can be used to rank solar proposals from developers so projects will score higher if they “consider strategies that avoid sensitive or protected land, minimize impacts to agricultural and environmental resources, and provide community benefits and collaboration.”^491^ The role solar plays in the overall mix of renewable energy technologies implemented in New York State will depend on numerous factors, including economics, community reaction to proposed site locations, the speed with which transportation and heating are electrified, policy direction at all levels of government, and the relative rate of technological advances in solar compared to advances in other energy technologies.

#### 5.5.2 Carbon markets

A carbon market is “an economic framework that supports the buying and selling of environmental commodities that signify greenhouse gas emission reductions or sequestration.”^492^ The number of carbon market opportunities that pay farmers and other participants to generate carbon offsets for use in carbon markets has increased.^493^ Carbon farming can be defined as “the implementation of a land management strategy for the purposes of reducing, sequestering, and mitigating greenhouse gas emissions on land used in support of a farm operation and quantifying those greenhouse gas benefits.”^322^ A carbon farm plan combines whole‐farm planning and resource assessment in a comprehensive planning framework to reduce greenhouse gas emissions and sequester carbon.

Carbon market programs are set up to provide financial compensation for participants implementing climate‐smart practices that reduce greenhouse gas emissions associated with agriculture or that sequester carbon in soil and plants.^492^ Soils have different carbon and nutrient storage capacities; therefore, an important consideration for setting up carbon markets and farm plans is the need to establish a baseline soil organic carbon content to determine changes in individual fields, so credible carbon credits that are tradable can be recorded.^457^, ^494^ Protocols, accounting, and verification processes that are equivalent also need to be established so soil organic carbon and greenhouse gas removal credits are comparable.^495^ For New York State farmers, the conversation around carbon markets, credits, and farm planning is in its infancy but increasing.^496^ This will be a topic of discussion for years to come, especially considering the aggressive targets outlined in the Climate Act.

It is important to acknowledge that there are significant justice and equity considerations around carbon markets. Research shows that carbon pricing and trading exacerbates existing inequalities within communities often dominated by people of color and low‐income households.^495^, ^497^ As carbon markets in agriculture continue to grow in New York State (and beyond), considerations about how they are structured and who they affect must be at the forefront of the discussion.

### 5.6 Efficacy of adaptation strategies

The rate and intensity of climate change calls for farmers to adopt new adaptation practices on their farms.^3^ As farmers implement these strategies, it is essential to evaluate whether the strategies are effectively helping farmers increase their resilience to climate impacts.

Many farmers can offer anecdotal evidence about the perceived benefits of an adaptation strategy. For example, farmers feel that installing tile drainage is costly, but during years when the tile drainage avoids crop loss due to excessive moisture, farmers feel the investment in this practice is “money in the bank.”^434^ Farmers have also commented that by investing in larger equipment they are able to cover more acreage quickly and capitalize on the short planting and harvesting windows that are becoming more common. More than 70% of New York State's farmers surveyed in 2017 felt that implementing practices such as planting cover crops, reducing tillage, using soil amendments, leaving crop residues on the field, and/or shifting crop rotations lessened the impact of heavy rainfall events during that year's wet growing season.^498^

Conversely, adaptation strategies sometimes cannot completely keep up with severe weather. Investment in irrigation systems is analogous to drainage but for dry conditions. A severe drought across New York State in 2016 left farmers reporting significant crop losses even for irrigated fruit and vegetable crops. Due to the length and severity of the drought, irrigation equipment was unable to keep up with crop water demand, although the severity of crop losses was diminished with irrigation, relative to the losses in unirrigated fields.^46^

Quantifying the effectiveness of a practice is difficult, and few studies to date have calculated the success of on‐farm climate adaptation practices. Through farmer interviews, AFT captured the win‐win benefits of improving soil health on three integrated crop–livestock operations in Western New York.^477^ AFT's case studies profiled the economic, soil health, water quality, and climate benefits associated with cover cropping, reduced or no tillage, and improving nutrient management. Results showed the benefit in net income, reduced greenhouse gas emissions, and decreased soil loss from implementing these practices. Also, the USDA has created an Adaptation Workbook that includes a five‐step process to assist farmers in identifying management goals and objectives and recording the feasibility and effectiveness of adaptation measures.^71^

Some adaptation strategies could have unintended negative consequences. For example, increased pest and disease pressures resulting from climate change will lead farmers to increase their use of pesticides. This will have both economic implications for the farmer and potential environmental implications for the public and surrounding ecosystems.

## 6 LOOKING AHEAD

### 6.1 Opportunities for positive change

When considering the impacts of climate change on New York State's agriculture sector, it is important to note that there may be some positive outcomes or opportunities for farmers. For example:

- Projections show that the state will continue to have adequate access to water when many other agricultural areas of the country will not.
- Increased egg production during warmer winters could help offset impacts on egg production from heat stress and extreme precipitation.^1^
- Warmer temperatures, longer growing seasons, and increased atmospheric carbon dioxide could increase yields for some crops^36^ and offer opportunities for double‐cropping.^8^

Several agricultural practices, most notably improvements to soil health, can provide “win‐win” benefits or synergies for farmers by simultaneously contributing to both climate change resilience and mitigation. For example, increasing soil organic matter in fields by using cover crops or reduced tillage can lead to improved erosion control, nutrient management, and water‐use efficiency.^499^ Likewise, reducing the number of tractor passes over fields by switching to no‐till practices can improve soil health, reduce compaction, increase soil carbon sequestration, and reduce greenhouse gas emissions, ultimately saving farmers time and money.^500^

Farmers who are innovative and use sustainable practices will have increasing opportunities as farm organizations and government agencies in the United States and globally enact policies in support of climate change action. Funding and extension support for “climate‐smart” practices has grown, and farmers who embrace these and other sustainable practices will increasingly be able to capitalize on market demand from socially conscious consumers. In 2022, the USDA announced the new Partnerships for Climate‐Smart Commodities funding opportunity, which provides funding for “pilot projects that create market opportunities for commodities produced using climate‐smart practices.”^468^ New York State's Climate Resilient Farming Program provides cost share for farmers to make mitigation and adaptation changes on the farm.^7^ Companies such as Ben and Jerry's and Chobani have worked with their suppliers to enact and track sustainability metrics such as providing fair wages for workers, using climate‐smart practices such as no‐till and cover crops, and reducing greenhouse gas emissions^501^, ^502^; the companies then can showcase their commitment to addressing climate change. In this landscape, progressive farmers and companies will be able to increasingly market their products as climate‐friendly or carbon‐neutral.^503^

### 6.2 Emerging topics and research needs

Researchers are studying the specific ways in which climate change has already affected and will continue to affect agriculture across New York State. Farmers and all stakeholders working in the sector need to work together to devise, test, and improve new technologies and techniques to adapt to a changing climate. This assessment revealed several gaps in knowledge that would be valuable to fill with new research in the years ahead. Such gaps include answering the following questions:

- What are the impacts of incentive/financial assistance programs and carbon markets on the adoption of climate adaptation practices by farmers and the effectiveness of the practices implemented?
- How does climate change specifically affect crop yields and agricultural product quality (including nutritional value) in New York?
- How often do mycotoxin outbreaks occur and what is their impact on grain production?
- What opportunities exist to collect experimental data on both current and new pests and invasive species found in different commodities across the state? These data can assist in the validation of prediction models.
- Can food production be a carbon sink? How can food be produced so it is more resilient to climate change and better support the farmers who grow it?
- What climate‐smart practices are farmers adopting? What are the barriers to and opportunities for farmers’ adoption of new practices?
- What are the long‐term measured benefits of climate‐smart practices on farms’ resilience and sustainability?
- Can agricultural policies be synergistic with funding opportunities? What are the impacts of new climate policies on the New York State agriculture industry?

### 6.3 Conclusions

There is no doubt that climate change is affecting virtually all aspects of the agriculture sector across New York State. Over the past decade, climate impacts have intensified, exacerbating the existing stressors that farmers face. Most challenging is the increased variability and uncertainty of weather events; farmers rely on the weather, and this heightened uncertainty makes it difficult for them to plan appropriately. Farmers increasingly recognize that climate change is occurring, and they are experiencing the impacts. However, farmers are innovative and resilient, implementing adaptation practices where possible, considering cropping systems, management practices, financial constraints, and other factors. Farmers have a diverse skill set, ingenuity, and generational knowledge that allows them to adapt to ongoing challenges so their businesses can remain profitable and sustainable. With adequate technical and financial resources, farmers will have an even greater chance to respond to the myriad challenges posed by climate change and provide the food, feed, fiber, and fuel that New Yorkers rely on every day.

## 7 TRACEABLE ACCOUNTS

Traceable accounts examine each key finding in depth. They provide citations that support each assertion and present the authors’ assessment of confidence in each finding.

### 7.1 Key Finding 1

**The most severe impacts of climate change to the agriculture sector are associated with extreme precipitation, short‐term drought, heat stress, warmer winters, late spring freezes, increased pest pressures, and increased production costs**. Extreme precipitation damages crops, fields, and farm infrastructure; short‐term drought reduces crop yields and causes water shortages; heat stress affects livestock, crops, farmers, and farmworkers; spring freezes cause losses in perennial fruit crops; and increased weed, disease, and insect pressures cause crop damage. Projected increases in temperature and precipitation extremes will cause these impacts to become more severe over time.

#### 7.1.1 Description of evidence base

The key finding and supporting text summarize the extensive evidence documented in the peer‐reviewed literature, including in other climate reports providing comprehensive assessments of the wide range of impacts on agriculture.^3^, ^16^, ^36^, ^39^, ^71^, ^504^, ^505^ The case studies, quotes from farmers, and cited peer‐reviewed articles and popular media^45^, ^314^, ^315^ throughout the chapter provide direct evidence regarding the impacts already being experienced.

The evidence base for heat stress to animals,^34^, ^164^, ^167^, ^188^ crops,^90^, ^142^, ^143^ and people^345^, ^349^, ^377^ is very strong.

Evidence of how climate change affects weeds is extensive, as is the evidence supporting predicted changes.^75^, ^76^, ^77^, ^257^, ^258^, ^259^, ^260^, ^275^, ^276^ How climate change affects pest emergence, populations, distributions, and diseases is well reported in the literature.^18^, ^106^, ^249^, ^274^, ^336^, ^338^

#### 7.1.2 New information and remaining uncertainties

The projected severity and frequency of extreme temperature and precipitation events at any given location cannot be fully determined due to uncertainties in model projections. Whether a particular pest, weed, or disease outbreak will occur and become severe depends on a myriad of factors, including plant and insect biology, weather uncertainty and variability, and the degree of preparedness by farmers, researchers, extension agents, and other agricultural stakeholders. The New York State IPM Program monitors new pests.^506^

#### 7.1.3 Assessment of confidence based on evidence

Given the evidence base, the observed effects, and the impacts projected by state, regional, national, and international models, there is **very high confidence** that temperature, precipitation changes, and pest pressures are key drivers of current impacts and of increased severity of future impacts.

### 7.2 Key Finding 2

**Climate change is a threat multiplier for agriculture in New York State. Farmers already face many stressors such as tight profit margins and labor shortages**. Climate change exacerbates these stressors by producing more weather extremes, causing damage that requires unanticipated expenditures, and shortening operational windows. These stressors are further compounded in economically stressed, often rural communities and among historically underserved and vulnerable populations. Opportunities exist to address the negative effects, by both adapting to the direct climate impacts and managing the existing nonclimate stressors.

#### 7.2.1 Description of evidence base

Evidence of multiple and cascading climate impacts on the state's agricultural operations and systems is found throughout the literature, in reports, and in empirical evidence.^22^, ^313^, ^427^, ^507^ For example, an extreme winter storm in 2014 caused 5 feet of snow to fall in a short period of time in Western New York; this prevented milk trucks from reaching dairy farms, which caused farmers to dispose of milk and lose income.^361^ The state's recent announcement that the overtime threshold for farmworkers will be changed to 40 h per week by 2032 could have severe impacts on farms that are already economically stressed.^452^ This change means lost income for farmers and financial stress, which may lead to greater stress and anxiety and other mental health issues. In fact, reports of mental health concerns have increased among the state's farmers.^348^

Farmers and farmworkers are not only physically stressed during heat waves; they are also affected physiologically and socioeconomically.^342^, ^343^, ^344^, ^345^ Tribal communities that have lost productive agricultural lands and have been forced to move to lands that have lower economic value also experience greater exposure to climate‐related risks.^29^

#### 7.2.2 New information and remaining uncertainties

The exact climate impacts affecting a farmer will be farm‐specific. Quantifying the extent to which climate impacts exacerbate multiple stressors can be difficult. The magnitude of the consequences from compounding and cascading impacts is variable and uncertain.

#### 7.2.3 Assessment of confidence based on evidence

Given the evidence and remaining uncertainties, there is **very high confidence** that climate change has cascading impacts and compounding effects on New York State agriculture.

### 7.3 Key Finding 3

**Farmers and other agricultural stakeholders show awareness and acknowledgment of climate change impacts on agriculture**. Farmers and other agricultural stakeholders (e.g., extension agents, technical service providers, consultants) in New York are reporting increases in extreme weather events, variability, and uncertainty, which have disrupted common operations. Providing more information on anticipated changes and impacts will help farmers plan and remain profitable.

#### 7.3.1 Description of evidence base

Individuals in the agriculture sector acknowledge that the climate is changing—although not necessarily due to human causes—according to published survey data from New York State farmers;^21^, ^209^, ^434^, ^460^, ^498^ unpublished survey data^9^; numerous conversations with farmers (empirical evidence); and reports.^508^ For example, farmers in New York have noted increases in extreme weather, particularly heavy precipitation events. Survey data from 2009 indicate that the majority of crop producers did not accept the concept of climate change.^457^ For comparison, survey data from 2020 indicate that almost 80% of farmers believe climate change is occurring.^509^ A quote from one farmer focus‐group participant summarizes this well: “My personal feeling is that our climate is changing. I'm less alarmist than many but understanding more why and how may help us make better decisions for the future.”^434^

#### 7.3.2 New information and remaining uncertainties

While farmer awareness may be increasing, decisions about whether to adopt mitigation and adaptation practices vary widely, and personal experience with extreme weather, changes in the timing of seasonal events, and other climate‐related impacts affects decision‐making.^434^ Skepticism around anthropogenic climate change still exists within the agricultural community, and this has also been reported in the literature, predominately among midwestern farmers.^72^, ^453^, ^461^, ^462^ Research, extension, and policy efforts are ongoing to develop resources and tools to assist farmers in decision‐making.

#### 7.3.3 Assessment of confidence based on evidence

Given the evidence and remaining uncertainties, there is **high confidence** that farmers and agricultural stakeholders are becoming more aware that the climate is changing and becoming less predictable and more variable. However, beliefs about climate change continue to influence farmers’ implementation of mitigation and adaptation strategies.

### 7.4 Key Finding 4

**Farmers are starting to implement and invest in practices that make their farm businesses more resilient to climate extremes**. Adaptation strategies depend on farm location and size, observed climate impacts, commodities produced, farm size, and costs. Many of these strategies, such as improving soil health, are beneficial for farms to adopt regardless of climate change and can also provide the cobenefits of reducing greenhouse gas emissions. While these adaptations are unlikely to fully alleviate the future climate impacts projected for New York, they are key to making the state's farms more resilient.

#### 7.4.1 Description of evidence base

Empirical evidence from conversations with farmers and farm visits; fact sheets; survey data; and the peer‐reviewed literature supports that farmers are using a variety of practices to adapt to climate change and increase farm resilience.^32^, ^434^, ^477^ Many resources are available that describe the types of adaptation practices available to farmers across a range of farm sizes and commodities grown in the state.^1^, ^71^, ^510^ Farmers’ personal experience with extreme weather events impacts adaptation strategy implementation.^434^, ^459^

#### 7.4.2 New information and remaining uncertainties

Numerous agricultural adaptation strategies exist, but the extent to which individual farmers will adopt these strategies varies. Identifying and implementing strategies is a continuous process, undertaken as a part of farm planning. Belief in and perceptions of climate change influence a farmer's willingness to implement adaption strategies.^434^, ^459^ Researchers, industry professionals, agricultural organizations, extension agents, and others in this sector are developing new techniques, practices, and resources to help the agriculture industry adapt to climate change. However, the effectiveness of a given adaptation strategy on all farms is not known.

#### 7.4.3 Assessment of confidence based on evidence

Given the evidence base and remaining uncertainties, there is **high confidence** that, when possible, farmers are implementing and investing in practices that make their farm businesses more resilient to climate extremes.

### 7.5 Key Finding 5

**Enhanced technical support, financial assistance, and research are crucial to increase the adaptive capacity of farms across New York State**. Farms will face greater risk of physical, social, and economic losses due to climate change without more support to implement adaptation measures. Active engagement between policymakers, farmers, and other agriculture stakeholders can help shape climate and agricultural policies and programs that are realistic for farm businesses.

#### 7.5.1 Description of evidence base

Through the peer‐reviewed literature and discussions with farmers, policymakers, and other agricultural stakeholders, it is clear that there is a need for greater technical support, funding, incentives, and research to help farmers adapt to increasing climate change impacts.^21^, ^434^, ^511^, ^512^, ^513^, ^514^ Most New York farmers feel they do not have the financial capacity to deal with climate‐related risks.^9^ Adaptation on small farms in particular will thus require active support in the form of grants, financing, and other financial incentives. Farmers also need specific information on how climate change affects their farms, as well as training on farm‐specific adaptation strategies and access to technical assistance to implement the strategies.^9^, ^465^

Public policies play a critical role in influencing adaptive capacity. Recent policies and programs at the state and federal levels emphasize the need for stronger actions around climate change, but the funding accompanying these policies is often inadequate to assist farmers with adaptation or to provide technical assistance. While funding for farmers from the state's Climate Resilient Farming Program more than tripled in 2022, funds can only be applied to three specific types of practices, and cost‐share funds have only been available to assist 200 farms as of 2022.^515^ New York State passed the Soil Health and Climate Resiliency Act in 2021 to assist farmers in improving their soil health, reduce the effects of farming on climate change, and adapt to and mitigate climate impacts. The New York Soil Health Initiative managed by Cornell University, with support from NYSDAM, is an essential source of research, networking, education, and outreach for farmers across the state. Also, there are possible synergies in coupling greenhouse gas emissions reduction and carbon sequestration efforts and payments for adaptation strategies, especially given the ambitious greenhouse gas reduction targets set by the state under the 2019 Climate Act. These synergies could help to support the sustainability of agriculture in the state, especially for the state's dairy farms (Dairy Industry Expert [2022, July, Personal communication]). In 2022, the USDA rolled out a competitive grants program, Partnerships for Climate‐Smart Commodities, with projects awarded up to $100 million. Several projects have been funded in New York State. The program has the potential to spur tremendous progress on the adoption of CSA in New York, but it will be imperative to provide adequate training and technical assistance for farmers, to support coordination between stakeholders, and to support research on the long‐term benefits of climate‐smart practices.

#### 7.5.2 New information and remaining uncertainties

While significant action has been taken in recent years, there is a need for expanded research and a greater understanding of the efficacy of new practices (e.g., electrification, batteries, soil health, carbon markets, manure management practices that reduce methane). Both basic and applied research is needed around the state on the permanency of carbon sequestration, considering different soil types, crops, climate conditions, and management practices. Full life cycle assessments of new practices are also needed, and these must align with policies and funding support available to farmers. Lastly, social science research is critical to understanding the barriers to the adoption of new technologies and practices as well as farmer perceptions of climate change.

#### 7.5.3 Assessment of confidence based on evidence

Given the evidence and remaining uncertainties, there is **moderate confidence** that additional technical and financial support, as well as focused research, will help increase the adoption of adaptation measures by farmers. There is **high confidence** that a need exists for policymakers to pass science‐based agricultural climate change policies.

## AUTHOR CONTRIBUTIONS

D.A.: Drafting, revising, and editing the manuscript; interviews; manuscript compilation and review; general supervision. A.M.C.: Drafting, revising, and editing the manuscript; interviews; drafting dairy case study; manuscript review. A.C.: Drafting, revising, and editing sections related to pests, weeds, pathogens, and pollinators. J.C.: Drafting, revising, and editing sections related to field crops; drafting field crop case study. A.O‐B.: Drafting, revising, and editing sections related to economics and labor. G.P.: Drafting, revising, and editing sections related to perennial fruit crops; drafting tree fruit case study. J.S.: Reviewing sections related to urban agriculture. B.W.: Drafting, revising, and editing sections related to dairy, livestock, and livestock products. E.W.: Drafting, revising, and editing sections related to policies; general manuscript review. The manuscript underwent an independent peer review, and the final submitted version was approved by all authors.

## COMPETING INTERESTS

The authors declare no competing interests.

### PEER REVIEW

The peer review history for this article is available at: https://publons.com/publon/10.1111/nyas.15192
